# Sitting less and moving more for improved metabolic and brain health in type 2 diabetes: ‘OPTIMISE your health’ trial protocol

**DOI:** 10.1186/s12889-022-13123-x

**Published:** 2022-05-10

**Authors:** Christian J. Brakenridge, Paul A. Gardiner, Ruth V. Grigg, Elisabeth A. H. Winkler, Brianna S. Fjeldsoe, Mia A. Schaumberg, Neville Owen, Elizabeth G. Eakin, Stuart J. H. Biddle, Marjory Moodie, Robin M. Daly, Daniel J. Green, Neale Cohen, Len Gray, Tracy Comans, Matthew P. Buman, Ana D. Goode, Phuong Nguyen, Lan Gao, Genevieve N. Healy, David W. Dunstan

**Affiliations:** 1 Baker Heart & Diabetes Institute, 99 Commercial Rd, Melbourne, VIC 3004 Australia; 2 Australian Catholic University, Mary Mackillop Institute of Health Research, Melbourne, Australia; 3 University of Southern Queensland, School of Health and Medical Sciences, Ipswich, Australia; 4 University of Southern Queensland, Centre for Health Research, Springfield, Australia; 5 The University of Queensland, Centre for Health Services Research, Brisbane, Australia; 6 The University of Queensland, School of Human Movement and Nutrition Sciences, Brisbane, Australia; 7 The University of Queensland, School of Public Health, Brisbane, Australia; 8 University of Sunshine Coast, School of Health and Behavioural Sciences, Sunshine Coast, Australia; 9 Sunshine Coast Health Institute, Sunshine Coast Hospital and Health Service, Birtinya, Australia; 10 Swinburne University, School of Health Sciences, Melbourne, Australia; 11 Deakin University, School of Health and Social Development, Melbourne, Australia; 12 Deakin University, Institute for Physical Activity and Nutrition, School of Exercise and Nutrition Sciences, Melbourne, Australia; 13 University of Western Australia, School of Sport Science, Exercise & Health, Perth, Australia; 14 Arizona State University, College of Health Solutions, Tempe, USA; 15 School of Public Health & Preventive Medicine, Monash University, Melbourne, Australia

**Keywords:** Type 2 diabetes, Sedentary behaviour, Glycaemic control, Cognitive function

## Abstract

**Background:**

Clinical practice guidelines recommend that adults with type 2 diabetes (T2D) sit less and move more throughout the day. The 18-month OPTIMISE Your Health Clinical Trial was developed to support desk-based workers with T2D achieve these recommendations. The two-arm protocol consists of an intervention and control arms. The intervention arm receives 6 months health coaching, a sit-stand desktop workstation and an activity tracker, followed by 6 months of text message support, then 6 months maintenance. The control arm receives a delayed modified intervention after 12 months of usual care. This paper describes the methods of a randomised controlled trial (RCT) evaluating the effectiveness and cost-effectiveness of the intervention, compared to a delayed intervention control.

**Methods:**

This is a two-arm RCT being conducted in Melbourne, Australia. Desk-based workers (≥0.8 full-time equivalent) aged 35–65 years, ambulatory, and with T2D and managed glycaemic control (6.5–10.0% HbA1c), are randomised to the multicomponent intervention (target *n* = 125) or delayed-intervention control (target *n* = 125) conditions. All intervention participants receive 6 months of tailored health coaching assisting them to “sit less” and “move more” at work and throughout the day, supported by a sit-stand desktop workstation and an activity tracker (Fitbit). Participants receive text message-based extended care for a further 6-months (6–12 months) followed by 6-months of non-contact (12–18 months: maintenance). Delayed intervention occurs at 12–18 months for the control arm. Assessments are undertaken at baseline, 3, 6, 12, 15 and 18-months. Primary outcomes are activPAL-measured sitting time (h/16 h day), glycosylated haemoglobin (HbA1c; %, mmol/mol) and, cognitive function measures (visual learning and new memory; Paired Associates Learning Total Errors [adjusted]). Secondary, exploratory, and process outcomes will also be collected throughout the trial.

**Discussion:**

The OPTIMISE Your Health trial will provide unique insights into the benefits of an intervention aimed at sitting less and moving more in desk-bound office workers with T2D, with outcomes relevant to glycaemic control, and to cardiometabolic and brain health. Findings will contribute new insights to add to the evidence base on initiating and maintaining behaviour change with clinical populations and inform practice in diabetes management.

**Trial registration:**

ANZCTRN12618001159246.

**Supplementary Information:**

The online version contains supplementary material available at 10.1186/s12889-022-13123-x.

## Background

Type 2 diabetes (T2D) is a major cause of premature mortality and morbidity due to cardiovascular, renal, ophthalmic and neurological diseases [1]. Adults with T2D have a heightened risk of absenteeism and inability to work, which increases with diabetes duration [2]. For those with T2D, the clustering of chronic conditions, such as cardiovascular disease and dementia, can substantially impact quality of life [3]. Significantly, those with T2D have a 73% increased risk of developing dementia [4]. Diagnosis of T2D is associated with an earlier onset of dementia by an average of 2.5 years, and as such has been identified as a key modifiable risk factor for dementia in later life [5]. Within this context, greater emphasis has been directed to mid-life prevention initiatives in T2D [6]. Optimising glycaemic control is a primary management consideration to reduce and prevent the impact of multiple morbidities [7], including dementia [8].

Participation in regular physical activity is considered a core element for glycaemic control and diabetes management [9]. However, estimates indicate that only one in four adults with T2D achieve the minimum physical activity levels recommended, while one in three with T2D report doing no moderate or vigorous intensity physical activity at all [10]. Furthermore, recent evidence shows that those with T2D can spend some 10 h of their waking hours in sedentary behaviours (sitting, lying or reclining) with low energy expenditure, an amount significantly greater than those without T2D [11]. The detrimental impacts of sedentary behaviour on health are broad [12], including an increased risk of premature mortality [13]. Sedentary time is also associated with poorer glycaemic control in people with T2D [14].

Epidemiological evidence demonstrates that replacing periods of sitting, particularly prolonged sitting time, with stepping is associated with improved glucose and insulin metabolism [15, 16], and lower occurrence of metabolic syndrome [17]. Recent experimental studies in adults with T2D have also shown that interrupting prolonged sitting with frequent short bouts of light-intensity physical activity (e.g., walking, simple resistance or muscle strengthening activities) can lead to substantial reductions in acute post-meal glycaemic responses [18]. In the free-living setting, interrupting sitting time with regular standing or light-intensity activities has resulted in improved 24-h glucose levels compared to engaging in structured exercise alone [19].

To date, controlled intervention trials designed to support adults to reduce their sedentary time have typically targeted the environment (such as through installing sit-stand workstations), the individual (such as through education and prompts), or a combination of both [20]. A recent meta-analysis found that both multicomponent and single-component interventions typically reduced sitting time by 30–60 min per day, predominantly achieved by replacing sitting with standing [21]. Importantly, a dose-response effect has been observed in workplace setting-based interventions, with the higher the exposure to the intervention, the greater the reduction in sitting time, with minimal evidence of either compensation or generalisation outside of the intervention setting [22]. Therefore, initiatives aimed at further reducing time spent sitting and increasing overall physical activity should target behaviours across the whole day, together with consideration of the contexts where the bulk of sitting time occurs (e.g. workplace, domestic).

In addition to understanding such behaviour changes, it is important for intervention trials to evaluate effects of the behaviour change on biological attributes associated with the risks and complications of T2D. The findings of a meta-analysis of interventions targeting sedentary behaviour reductions alone or in combination with increases in physical activity has shown promising, albeit modest, beneficial effects on weight, waist circumference, percent body fat, systolic blood pressure, insulin and HDL levels [23]. However, to date, studies of working age adults have had limited representation of those with clinical conditions such as T2D [23]. Furthermore, there is a paucity of studies intervening for 12 months or more and few have included maintenance evaluations from which to consider sustainability and longer-term effectiveness. As T2D contributes to higher risk for dementia over the lifespan, evaluating maintenance and the potential for long term sustainability of behaviour change is a key consideration.

The OPTIMISE Your Health trial (trial duration – 18 months) is examining the effectiveness of a series of interventions targeting sitting less and moving more across multiple contexts (see footnote). The interventions target behavioural, metabolic and brain health outcomes, and each phase evaluated for cost-effectiveness. The respective interventions are: a 6-month multicomponent intervention; a 6 month extended care intervention; and an abbreviated intervention delivered to delayed intervention controls. This manuscript provides an overview of the OPTIMISE Your Health trial, including the aims, intervention methods and evaluation protocols and contingency with the COVID-19 pandemic.

FOOTNOTE: The OPTIMISE Your Health trial was originally conceived as a 6-month trial inclusive of an intensive sedentary behaviour reduction intervention. By leveraging the merits of the original grant, and seeking to investigate the longer term implications for dementia risk in people with T2D, a second research grant was awarded to the team which allowed for an expanded protocol with extended care to 12 months as well as assessment of maintenaince until 18 months post baseline. Finally, a third research grant was secured to explore an abbreviated intervention: OPTIMISED, to be delivered to delayed intervention control participants at 12–18 months with the aim of informing rapid scale-up. More information is available in electronic supplementary material (Additional file 1).

### Aims and hypotheses

The trial aims to determine the effectiveness, for office workers aged 35–65 years with T2D, of a multi-component intervention with extended care (compared to a control condition, on primary and secondary outcomes.

Secondary aims are to:

- assess maintenance of outcomes after cessation of study contact (with participants retaining the activity tracker and workstation components only)
- conduct an economic evaluation to determine the cost effectiveness of the intervention and to estimate the broader social and economic benefits of delaying dementia onset; and,
- develop and test (in the delayed intervention control arm) a modified version of the intervention suitable for wider-scale implementation (OPTIMISED; the abbreviated form of the intervention).

It is hypothesised (two-tailed) that:

1. The intervention and control group participants will differ in changes in the primary and secondary outcomes (0–6 months)
2. The intervention and control group participants will differ in changes in the primary and secondary outcomes (0–12 months)
3. Changes in primary and secondary outcomes achieved by completion of the intervention will be maintained at 18 months (i.e., no significant 12–18 month change)
4. The OPTIMISE intervention will be cost-effective (measured against the commonly used Australian benchmark of less than $50,000 per quality-adjusted life year gained)
5. The OPTIMISED intervention will be feasible and acceptable (primary) and result in pre-post change (12–18 months) in behaviour for delayed intervention participants.

## Methods/design

The Optimise Your Health trial has been iteratively developed over time following the securement of three successive research grants (see supplementary material: Additional file 1). Each grant relates to separate trial phases and interventions with distinct aims, hypotheses, and outcome measures. Recruitment for the original OPTIMISE protocol commenced in June 2019 with 27 participants recruited and completing the 6 month original iteration. In June 2020, recruitment commenced for the extended 18 month trial, which was inclusive of all phases described above. A SPIRIT checklist for standardized protocol items was followed in writing this manuscript (Additional file 11).

### Study design and randomisation

The OPTIMISE Your Health trial is a two-arm individually randomised controlled trial. Randomisation to either the intervention or delayed intervention arm is undertaken in random blocks of sizes 4–8, stratified according to whether participants are taking either 0–1 or 2+ hypoglycaemic medications. Randomisation is automated via REDCap software at the end of the baseline assessment. Due to the nature of the intervention, it is not possible to blind participants or assessors to group assignment.

**Fig. 1:**
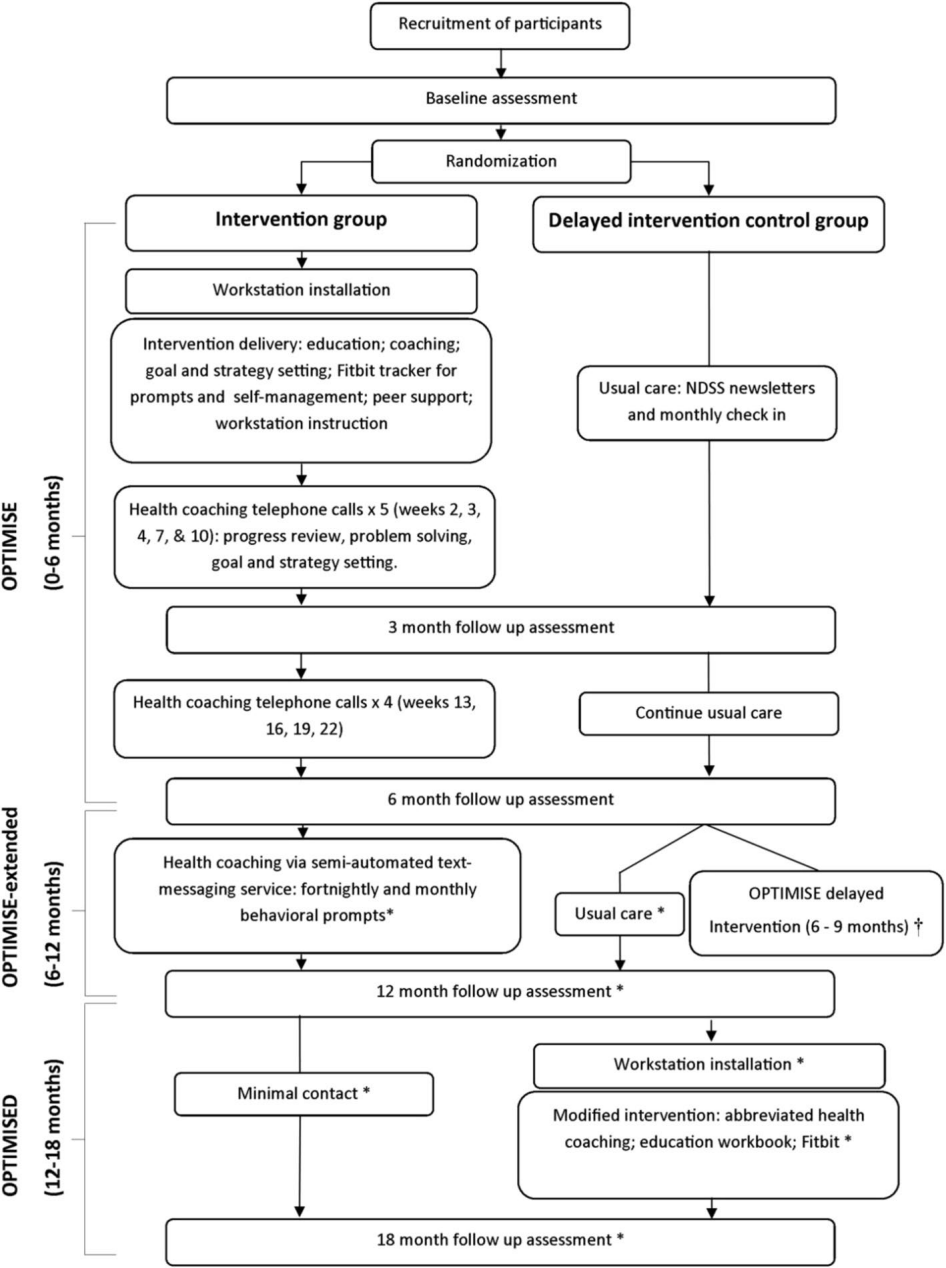
OPTIMISE 18-month trial protocol design depicting the OPTIMISE phases, assessment timeframe, intervention and delayed intervention control group receiving an abbreviated intervention (OPTIMISED). Figure depicts the full protocol as intended, not including any adaptations due to COVID-19 pandemic. NDSS = National Diabetes Services Scheme; *Expanded protocol only; † Discontinued in expanded protocol (*n* = 27 completed), participants provided with a workstation and education only

The trial protocol includes five assessment time-points: baseline, 3- months, 6- months, 12-months, 15- months, and 18- months. Database(s) facilitating intervention management and data collection are undertaken through REDCap — an approved data management platform (version 11.1.25) [24]— with further data stored securely but accessibly to the multidisciplinary multinational research team through The University of Queensland’s Research Data Management System. Ethics approval for both the original and expanded protocols were granted by Alfred Health Human Ethics Committee (Melbourne, Australia), The University of Queensland Institutional Human Research Ethics Committee (Brisbane, Australia), and the University of the Sunshine Coast Human Research Ethics Committee (Sunshine Coast, Australia). The OPTIMISE Your Health trial has been registered with the Australian New Zealand Clinical Trials Registry (ANZCTRN12618001159246; date of registration: 07/03/2018 URL: https://www.anzctr.org.au/Trial/Registration/TrialReview.aspx?id=375487). The trial is undertaken in accordance with CONSORT guidelines (http://www.consort-statement.org/), with Fig. 1 showing the overall study design.

### Study setting / population

Participants are adults aged 35–65 years with clinically diagnosed and managed T2D (recent HbA1c test between 6.5 and 10.0%) who are working ≥0.8 full time equivalent (FTE) in predominantly (≥75%) desk-based work in the local Melbourne area. Participants are excluded based on the following criteria: not English speaking, pregnant, using insulin to treat their diabetes, or have high physical activity (maintained for at least 3 months: ≥30 min of moderate-to-vigorous intensity physical activity [MVPA] per day and/or ≥ 30 min of strength training on at least two separate days of the week). Complete details of the eligibility criteria are provided in the electronic supplementary material (Additional file 3). The trial aims to recruit 125 participants per arm to satisfy sample size requirements. In-person clinical assessments are conducted at the Baker Heart & Diabetes Institute in Melbourne, Australia.

### Recruitment sources

Recruitment commenced in June 2019 and is ongoing through to June 2024 using several different recruitment options, including but not limited to:

- Disseminating study promotional material into clinics and hospitals within the local community;
- Online social media advertising performed both independently and by an external third party recruitment company
- Television and newspaper feature articles
- Mailing people registered on the National Diabetes Services Scheme (NDSS) database; and,
- Contacting patients attending the adjacent diabetes clinic (in person or by telephone).

### Screening and consent

Screening for eligibility and gaining consent involves a multistage process. Initially, potential participants are emailed a link to a brief survey containing questions concerning eligibility. Those whose surveys indicate they may be eligible receive a follow up telephone call from research personnel to confirm. Once confirmed, participants are sent further participation information (Additional file 2), and an employer permission form via email to forward to their employer, which is completed online. This employer permission form explains the relevant study requirements and informed consent for the study and permission for installation and use of a sit-stand workstation in the workplace. Participants are requested to send a photo of their current workstation setup to assist in desk installation. If a participant is self-employed or working from home they can sign the form themselves. Thereafter, participants are asked for results of any HbA1c blood test they have had in the last 4 weeks, or are provided with a pathology request slip and asked to arrange a non-fasting blood test (glycosylated haemoglobin; HbA1c%) at their nearest Melbourne Pathology centre (at no cost to the participant). Those within the eligible HbA1c% range (6.5–10.0%) are then requested to give final informed consent (written or electronic; Additional file 2) and have their baseline visit scheduled.

### Impact of the COVID-19 pandemic on the trial

Intensive lockdown restrictions occurred in March 2020 leading to the suspension of recruitment. All participants enrolled in the trial during these periods were assessed according to remote COVID-safe practices. In October 2020 the lockdown restrictions ended and resumption of recruitment and the expanded protocol began.

### Intervention

#### Background

The OPTIMISE intervention is based on the Stand Up Australia workplace intervention [25, 26]. However, it has been extended from a primarily workplace focus to include messaging targeting sitting less and moving more across the whole day. The intervention is grounded in social cognitive theory, with the key constructs targeted being: self-efficacy; socio-structural factors (barriers and facilitators); and outcome expectancies (physical, social and self-evaluative) [27]. Consistent with the socio-ecological model of sedentary behaviour [28], which highlights the multiple inter-related influences on this behaviour; the intervention targets multiple levels of influence on behaviour (environment, intra-personal, inter-personal). In contrast to the Stand Up Australia intervention [25], the OPTIMISE intervention does not specifically include workplace organisational strategies. The intervention components are informed by the Behaviour Change Wheel (BCW) and the associated COM-B system, within which capability, opportunity and motivation are postulated as interacting to stimulate behaviour change [29]. Previous feedback collected in the Stand Up Australia intervention [30] has demonstrated appropriate participant acceptance and feasibility of the key components including adaptations to the physical work environment, and the support of individual behaviour change.

#### Intervention targets

The key intervention messages are to “Sit Less” and “Move More”. The aim of the intervention is to support participants to achieve (by 6 months), and then to either progressively improve or maintain (at 12 and 18 months), their personalised goals for sitting less, actively breaking up sitting time, and moving more. Participants receive coaching to set incremental goals that gradually progress from their pre-existing levels at baseline towards the study targets. Key intervention targets are: at least half of daily waking time in upright postures (50% standing/stepping); at least one ‘active’ break from sitting per hour; and, at least 10,000 steps per day. An ‘active’ break is considered to be at least three minutes of walking (approximately 250 steps) or completion of simple body weight resistance exercise activities that have been adapted from previous experimental trials [18]: calf raises, squats, and single leg kickbacks, done in three sets of three 20-s bouts totaling approximately 3 minutes. Since excessive sitting and excessive standing may both be harmful [31, 32], 50% was chosen as a level that is a simple, heuristic approach that has been safely achieved by participants in our previous trials [33] and is 2 h/day lower than the average sedentary time reached by adults with type 2 diabetes [11], assuming a 16 h waking day. ‘Active’ breaks to interrupt sitting were promoted in recognition that not all sitting replacement activities have equal benefit [34, 35] with ambulation and resistance exercise showing particular benefit [36]. The 10,000 steps message is widely used in public health [37], noting benefits (including glycaemic control) for increased steps can occur below this threshold [38].

#### Intervention protocol and core components

The OPTIMISE intervention consists of health coaching (including education), a sit-stand workstation and an activity tracker (both provided by the trial) to encourage participants to sit less, as well as to move more and engage in active breaks. The intervention commences with workstation installation by third party installers approximately 2 weeks following baseline assessment. Immediately after installation, participants meet with their assigned health coach, via zoom, to discuss the intervention, demonstrate correct desk usage, and set up and demonstrate the workings of the Fitbit. The health coaching continues in person and via telephone throughout the first 6 months intensive phase (10 total contacts). They then receive tailored coaching via text message between 6 and 12 months (1–2 messages per week). Participants may seek other concomitant lifestyle interventions to manage their diabetes. After completing the trial, participants retain the sit-stand workstation and activity tracker.

#### Sit-stand workstation

Consistent with the Behaviour Change Wheel intervention functions of enablement and environmental restructuring [29], all participants are provided with an Ergotron^tm^ WorkFit-T/−TL Sit-Stand Desktop Workstation. The workstation weighs 22.5 kg and is placed on top of the existing work surface. It allows participants to easily and quietly alternate their working posture between sitting and standing whilst still interfacing with their computer. Participants are provided with either a single or dual arm monitor kit that allows their computer monitors to be directly affixed to the monitor platform. Both written and verbal ergonomic instructions are provided by the health coaches to aid in correct workstation usage [39]. Under work-at-home restrictions, the alternative protocol is for participants to take their workstations home when permitted by the employer.

#### Activity tracker (Fitbit)

In line with the Behaviour Change Wheel intervention functions of education and training, participants are provided with a Fitbit activity tracker and Fitbit smartphone application (app) for Android or Apple. The Fitbit’s main purposes are to encourage participants to move more, and to engage in active breaks from sitting. Participants are given a username and password to be used for the duration of the trial and provide permission for their data to be accessed by the project team. Upon completing the study, the account will be deactivated and participants will be instructed on how to create their own personal account. Each Fitbit is synchronized with Fitabase (Small Steps Labs, LLC; San Diego, CA, USA; http://fitabase.com), a third-party data management platform that supports top down supervision of study participants’ device usage and activity. The model of Fitbit used is the Fitbit Inspire HR, a wireless wrist-worn device that records and displays a range of outputs in real time (including daily step count, approximate resting heart rate, hourly activity / active breaks) — captured through proprietary algorithms applied to tri-axial accelerometery and heart rate (photoplethysmography) inputs — and provide vibration feedback based on the recorded data. The lithium polymer battery powers the device for approximately five days (depending on usage) before requiring recharging and collects data for up to 30 days (at which point it must be synced with the participant’s smartphone).

Using the device and app real-time monitoring, participants self-monitor attainment of their self-selected stepping and active breaks goals throughout the intervention and specifically during health coaching sessions. Hourly active break reminders are sent according to the participant’s preference (up to 14 per day). These reminders are produced by the Fitbit ten minutes before each hour finishes. Participants receive a vibration alert if they have not achieved at least 250 steps in that hour (their active break, if they have chosen walking rather than resistance exercises). Other active break strategies, such as simple resistance activities may be manually recorded using the Fitbit, however these are not automatically monitored by the Fitbit itself. Health coaches remotely monitor participant adherence to steps and active break goals with Fitabase, which supports tailored feedback during coaching sessions.

#### Face-to-face and telephone health coaching (0–6 months)

The individual health coaching component targets the Behaviour Change Wheel intervention functions of persuasion, education and training. Health coaches, all of whom have at least a bachelor level of training in exercise physiology, nutrition, or nursing, are trained in the delivery of the intervention, including motivational interviewing techniques [40]. A written script is maintained for intervention fidelity and consistency of delivery. Key aims of the health coaching are to build rapport, provide education about the importance of reducing sedentary behaviour and increasing movement throughout the day, encourage sustained self-management, and goal setting. In conjunction with the first health coaching session, participants are provided with an intervention handbook, containing written educational information and instructions (Additional file 4), an email containing basic tips to reduce sitting, a 2-min educational video describing the link between diabetes and sitting and activity levels, a video demonstrating how to perform the simple resistance activity breaks, and an activity feedback report (Additional file 5) generated from their most recent ten days of activity monitor (activPAL) wear. The health coaching consists of ten sessions (two in-person and eight via telephone; approximately 15–30 min in duration) over 6 months. Health coaches follow standardised outlines for each session set out in REDCap forms containing both the coaching script and embedded data-collection fields to further enhance intervention fidelity and collect data for process evaluation. The data collection is also used to guide subsequent health coaching sessions (e.g., goals, goal attainment). All health coaching sessions are delivered by the same health coach wherever possible and conducted at a time selected by the participant.

#### Coaching session 1 (face-to-face)

The first session, scheduled to take 50–60 min, occurs via video teleconferencing immediately after installation of the workstation. The session includes a brief discussion of the workstation relative to the expectations and needs of the participant, whereby the coach ensures the desk is installed to allow for correct usage habits and ergonomic positioning; demonstration of how to perform the simple resistance activities at the desk; provision, setup and demonstration of the activity tracker (see ‘Activity tracker (Fitbit)’); and, the behavior change motivational interviewing. A script of this interview is available in Additional file 7.

For the motivational interviewing, the health coach revises the participant’s personalised feedback report (Additional file 5) covering pre-existing sedentary behaviour and physical activity. Their behaviour at work, over the whole waking day, plotted by date and time is used to help set realistic goals. It is also used to identify and reflect on their “danger zones” (periods throughout the day of prolonged, unbroken sitting time), and choose relevant behavioural strategies targeting those danger zones. Participants are guided to form an action plan consisting of two goals (number of active breaks at work; total step count per day) and at least one strategy each to: sit less at work; sit less outside of work; move more during work; and, achieve the daily step count goal. Participants choose from a recommended list of strategies, or choose their own (Additional file 6). The coach supports the participant to make selections that conform to the SMART framework (Specific, Measurable, Achievable, Relevant, and Time-based), consider barriers and solutions, and for which the participant has adequate readiness and confidence to achieve (i.e., 8+ on a scale from a 1 (not ready / not confident) to 10 (ready / confident)). The health coach reviews the final action plan and any anticipated barriers with the participant. Following the session, a summary of the discussion is provided to the participant in a personalised email from their coach.

#### Sessions 2–6, 8–10 (telephone)

**Fig. 2:**
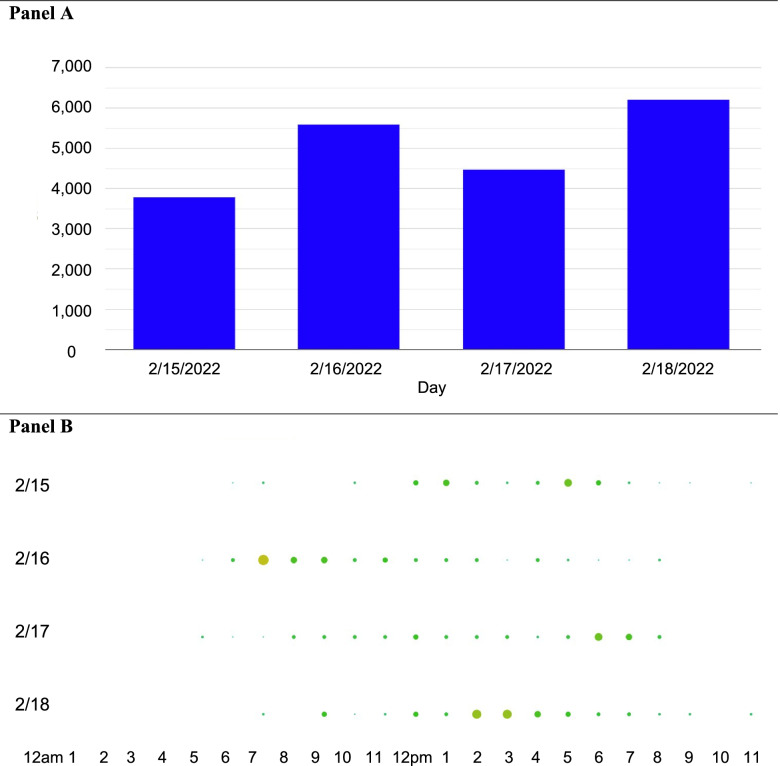
Monitoring participant’s Fitbit adherence and goal and strategy attainment in real-time with the Fitabase data management platform. Example participant Fitbit data depicted. Panel **A** depicts typical daily step count data across selected dates. Panel **B** depicts hourly break down of step accruement across selected dates representative of 24 h (0:00–23:59 pm). Traffic light system is depicted whereby large red dots denote high levels of steps, and small green dots indicate low level of steps in given hour

The seven telephone-delivered sessions occur on a tapered schedule: one call per week for the first three weeks reducing to one call every three weeks for the remaining 23 weeks (approximate timeframe). Each session lasts for a duration of 15 to 30 min. The sessions are designed to check on the participant’s progress (both subjectively and objectively with their Fitbit data as in Fig. 2), address problems with adherence, revise goals as needed, and reinforce goal attainment. Adoption of new strategies, and the progression of goals is encouraged by health coaches when participants report high engagement and success with their current plan.

#### Session 7 (face-to-face)

Coaches schedule an in-person session to coincide with their attendance at the Baker Institute, Melbourne for their 3-month clinical assessment. In a 30 min session, coaches enquire regarding participants’ overall experiences and their readiness to continue with the intervention, and provide feedback on how their their daily steps and active breaks have tracked over time for each week since the first health coaching session according to their Fitbit. Similar to the telephone sessions, participants’ strategies and goals to sit less and move more are revisited and revised as required. This session is offered via telephone when in-person visits are not possible (e.g., due to the COVID pandemic) or declined.

#### Extended care (tailored text messaging; 6–12 months; expanded protocol) – OPTIMISE

The 6–12 month intervention phase is designed to support maintained behaviour change (or continued improvement) after the 10-session intensive phase has been completed. This program, which is administered remotely, transitions participants from the face-to-face and telephone delivered health coaching to a tailored text message service using the web-based semi-automated platform, propelo™ (www.propelo.com.au). Coaching involves 24 weeks of text message contact and one telephone coaching session (midway) with their health coach.

The text message content has been informed from formative discussion with research staff, health coaches, and completed OPTIMISE intervention participants who were recruited before the expanded protocol was initiated. This formative research involved qualitative interviews and user-testing of draft text content. Key findings from this formative work highlighted the need for the text message program to: use supportive, non-judgemental language; continue to leverage off the rapport established between the health coach and participant during the intensive phase; and, ensure the texts are tailored to the participant’s Fitbit data. The texts target three key behaviour change strategies in three different types of text messages (see Table 1): prompt actions in real-time (via ‘Sit less prompts’ and ‘Move More promps’); promote ongoing self-monitoring of behaviour (via ‘Check-ins’); and, monitor and reward goal attainment (via ‘Check-ins’).

**Table 1:**
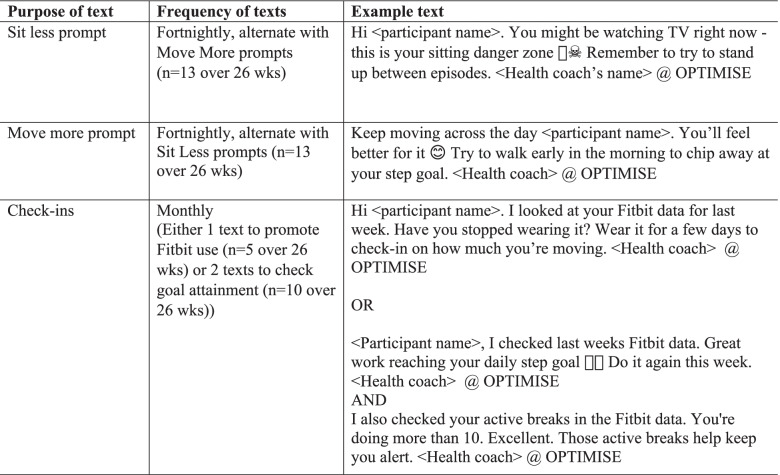
Overview of text messages for the OPTIMISE-extended phase of intervention (6–12 months)

During the final OPTIMISE telephone coaching sessions (#8), the health coach notifies participants that they will be receiving text messages over the next six months. Following the session, the coach completes a tailored survey translating the participant’s coaching goals into messaging-friendly “danger zones” (periods of the day or activities when prolonged sitting commonly occurs), strategies to sit less and move more, and goals for steps per day and active breaks per day. The appropriate days and times to send each type of message is also captured. Text messages are sent at a minimum frequency of once per week (see Table 1). The text message content is kept under 160 characters and is initially based on information from the tailoring survey, and subsequently, throughout the intervention, based on data from the participant’s Fitbit data accessed weekly through Fitabase. Either one or two check-in texts are sent monthly (on Mondays), based on whether the participant has met (yes/no/almost) their step goal and their active break goal in the previous week (Monday-Sunday) or their goal attainment could not be verified because the participant did not wear the Fitbit in the week prior. The telephone session with the coach midway through the text message program, involves a review of progress, and retailoring of the goals, danger zones, strategies, message days and times as needed.

### Control – delayed intervention

To minimise attrition prior to receiving their delayed intervention (at 12 months), the control participants receive contact in the form of a thank-you letter commencing two weeks after the baseline assessment then, monthly emails containing diabetes fact sheets, along with a follow-up phone call (verifying they received the fact sheet and enquiring whether they have any questions). The fact sheets are published by the NDSS, and provide a non-tailored diabetes-management advice. The fact sheets do not cover sedentary behaviour. Participants may seek other lifestyle interventions to manage their diabetes during this period. Following the completion of the 12 month assessment, control participants are provided with a modified version of the intervention (12–18 months). In the original protocol, delayed intervention (6–9 months) participants were provided with a sit-stand workstation and written education materials only. In the expanded protocol this is referred to as OPTIMISED (12–18 months). As with OPTIMISE, OPTIMISED includes environmental support via a sit-stand workstation, provision of a Fitbit, and health behaviour change coaching (one delivered by online video teleconferencing and two delivered by telephone). However, the behaviour change health coaching and educational materials are modified to be more suitable for scale-up and external delivery, based on consultation and collaboration with end users and stakeholders. The intervention adaptation process, and the resultant OPTIMISED intervention protocol, will be reported elsewhere.

### Primary outcomes

The primary outcomes are overall daily sitting time, and glycosylated haemoglobin (HbA1c) at 6 months, which were originally chosen in order to evaluate the intensive phase of the intervention (OPTIMISE). With the addition of the expanded protocol, cognitive function measures: visual learning and new memory (Paired Associates Learning Total Errors [adjusted]) were added as a primary outcome at 12 months.

### Secondary and exploratory outcomes

Secondary outcomes include: sitting time accumulation patterns (sitting time in prolonged bouts [≥30 min bouts], prolonged bouts at work, usual bout duration, alpha [unitless measure characterising the frequency distribution of sedentary bout durations]); sitting time at work; physical activity (active breaks from sitting, time spent standing, stepping, light-intensity physical activity, moderate-to-vigorous intensity physical activity) overall and during work; sleep (total sleep time, sleep efficiency, sleep onset latency, wakefulness after sleep onset); anthropometry (weight, waist, and hip circumference); body composition (fat mass, visceral fat mass, lean mass); fasting and postprandial glucose metabolism assessed as incremental area under the curve (iAUC) for glucose and insulin during a 2 h 75 g oral glucose tolerance test (OGTT); fasting lipid levels (LDL, HDL, total cholesterol, cholesterol ratio, triglycerides); blood pressure (systolic and diastolic pressure); vascular function (flow-mediated dilation and shear rate); inflammatory markers (high-sensitivity c-reactive protein [hs-CRP], interleukin [IL]-6, IL-1β, tumour necrosis factor – alpha [TNF-α], adiponectin, and leptin); neurotrophic factors (brain-derived neurotrophic factor [BDNF], insulin-like growth factor 1 [IGF-1]); and, additional cognitive function domains including reaction time, visual matching ability and visual recognition memory, reaction time, and working memory. Some of the survey measures are considered as outcomes, including the measures of anxiety and depression, musculoskeletal health, fatigue, workplace performance and satisfaction, and motivation. Outcomes regarding longer term trends in physical activity and sleeping are extracted from the Fitbit worn by intervention participants only.

### Data collection

Data collection occurs at baseline, 3, 6, 12, 15 and 18 months via: in-person clinical assessments; cognitive testing; device-based monitoring; and online questionnaires administered through REDCap; and, process data collected in REDCap and by the health coach in REDCap health-coaching forms. Table 2 shows the timepoints at which each component of the data-collection occurs under the original, expanded, and COVID-adapted protocols. Table 3 and Additional file 9 provide extensive details of the objective and questionnaire measures, as well as their intended purpose in the trial (e.g., primary outcome, secondary outcome, process outcome). The data collection procedures used for each of these components are indicated below.

**Table 2:** Data collection components and timepoint of collection

Data collection component		Timepoint					
		0 M	3 M	6 M	12 M*	15 M*	18 M*
Clinical assessment (in person)	Fasting venous blood draw ^a^	✓	✓	✓	✓		✓
	OGTT ^b^	✓	✓	✓	✓		✓
	Anthropometric ^b^	✓	✓	✓	✓		✓
	DXA ^b^	✓	✓	✓	✓		✓
	Blood Pressure ^b^	✓	✓	✓	✓		✓
	FMD ^b^	✓	✓	✓	✓		✓
	Cognitive Testing ^b^	✓		✓	✓		✓
Device-based monitoring	activPAL	✓	✓	✓	✓	✓	✓
	GT3X+	✓	✓	✓	✓		✓
	Activity Monitor Diary	✓	✓	✓	✓	✓	✓
Surveys (administered during clinic visit)	Health History Survey	✓					
	Medication Log ^a^	✓	✓	✓	✓		✓
	Allied Health Log ^a c^	✓	✓	✓	✓		✓
	OPTIMISE Survey ^a^	✓	✓	✓	✓	✓	✓
	Personal wearables questionnaire ^b c^	✓	✓	✓	✓		✓
	‘Typical 24 h’ Survey ^d^	✓	✓				
Surveys (self-completion)	Dietary intake questionnaire	✓	✓	✓	✓		✓
	Quality of Life Survey	✓	✓	✓	✓		✓
	Menstrual Status Questionnaire ^c^ (women only)	✓		✓	✓		✓
	Motivation for Physical Activity Survey ^c^	✓	✓	✓	✓		✓
	Motivation to Break Up Sitting survey ^c^	✓	✓	✓	✓		✓
	OPTIMISED Survey (delayed intervention) *					✓	✓
	COVID-19 impact questionnaire ^e^	✓		✓	✓	✓	✓
Implementation / intervention fidelity data	REDCap Metadata: instrument status, dates & times	✓	✓	✓	✓	✓	✓
	Fitbit usage		✓	✓	✓	✓	✓
	propelo™ message log: full record of all messages sent/received			✓	✓	✓	
	Health coaching forms		✓	✓			
	Tailoring forms			✓			
	Withdrawal and adverse events log ^f^	✓	✓	✓	✓	✓	✓

Abbreviations: *OGTT* oral glucose tolerance test, *DXA* dual energy x-ray absorptiometry; *FMD* flow-mediated dilation

* Expanded protocol only

^a^ Replaced with COVID-safe alternative during restrictions (self-completion questionnaire; self-report; no clinical visit, pathology visit HbA1c test only)

^b^ Dropped during COVID restrictions

^c^ Added part way through study

^d^ Subsample only - recruited first 30 participants, completed before expanded protocol began

^e^ Not timed with the assessments but triggered by events: soon after the Australian government implemented restrictions March 2020 (COVID-19 pandemic restrictions) or upon reporting of a withdrawal or adverse event

^f^ When reported

**Table 3:** Clinical and device-based measures collected in the in-person visit clinical assessments

Component	Key constructs	Measures	
		Primary / secondary outcomes	Other measures
Fasting blood draws	Glycaemic control	Glycosylated haemoglobin (HbA1c %, HbA1c mmol/mol)*	
	Fasting glucose metabolism	Glucose and Insulin	
	Fasting lipid metabolism	LDL, HDL, Total cholesterol, Triglycerides	
	Inflammatory markers	Hs-CRP, TNF-α, IL-6, IL-1B, Adiponectin, Leptin	IFNy, IL-10, IL-12, IL-8, IL-4
	Neurotrophic factors	BDNF, IGF-1	NGF, VEGF-A
	Genetic risk factors		ApoE4
2 h Oral Glucose Tolerance Test	Postprandial metabolism of glucose & insulin	iAUC glucose, iAUC insulin, 2 h post-prandial glucose, 2 h post-prandial insulin	
Anthropometric	Body weight & body composition	Weight, waist circumference, DXA assessed total and regional fat mass, lean mass, total body fat percentage, DXA software estimated visceral adipose tissue mass	Height, BMI
Vascular	Blood pressure & heart rate	Resting systolic blood pressure, diastolic blood pressure, mean arterial pressure	Resting heart rate
	Flow Mediated Dilation	Post-deflation peak vessel diameter compared to resting diameter (FMD%)	Shear rate (cm/s), Shear rate AUC
Cambridge Neuropsychological Test Automated Battery (CANTAB)	Visual learning and new memory	Paired Associates Learning total errors (adjusted)^b^ (PAL TEA), PAL total errors 12 shapes (adjusted)	PAL total errors, PAL total errors 12 patterns
	Reaction Time	Reaction Time (RTI) simple median reaction time, RTI simple median movement time & RTI median 5-choice reaction time, RTI median 5-choice movement time	RTI simple error score (all), RTI total error score (5-choice)
	Visual matching ability and visual recognition memory	Delayed Matching to Sample (DMS) percent correct, DMS median correct latency	DMS total errors, DMS total correct (12 s delay)
	Working memory	Spatial Working Memory (SWM) between errors, SWM between errors (12 boxes), SWM strategy (6–12 boxes)	SWM total errors, SWM total errors (12 boxes)
Cognitive function	Global cognition		Addenbrooke Cognitive Examination-Revised (ACE-R) Mini Mental State Examination
Device-based monitoring (thigh-worn activPAL)	Sedentary behaviour	Overall sitting time^a^, Overall prolonged sitting time (≥30 min), Work sitting time, Work prolonged sitting time	
	Sedentary time accumulation	Usual bout duration, Alpha (overall and work)	
	Active breaks	Active breaks (n per day overall and work)	
	Physical activity	Standing, Stepping, LPA, MVPA (all overall and during work)	
	Sleep	Total sleep time, Sleep efficiency	Sleep onset latency, Wakefulness after sleep onset
Device-based monitoring (wrist-worn Actigraph GT3X+)	Sleep	Total sleep time, Sleep efficiency	Sleep onset latency, Wakefulness after sleep onset

Abbreviations: *ApoE4* Apolipoprotein E4, *BDNF* brain-derived neurotrophic factor, *BMI* body mass index, *CANTAB* Cambridge Neuropsychological Test Automated Battery, *DXA* Dual X-ray absorptiometry, *FMD* flow-mediated dilation, *HbA1c* Glycosylated Haemoglobin, *HDL* High density lipoprotein cholesterol, *Hs-CRP* High-specificity C-Reactive protein, *iAUC* incremental area under the curve, *IGF-1* insulin like growth factor 1, *IL-1B* interleukin-1 beta, *IL-6* interleukin-6, *LDL* Low Density Lipoprotein Cholesterol, *LPA* light intensity physical activity, *MVPA* moderate-vigorous intensity physical activity, *TNF-α* Tumour necrosis factor alpha

^a^Primary outcomes for original OPTIMISE protocol concerning diabetes (primary endpoint = 6 months)

^b^Primary outcome for expanded protocol concerning dementia (primary endpoint = 12 months)

#### Device-based measurement of physical activity, sedentary behavior and sleep

Device-based measures of physical activity, sedentary behavior, sedentary behavior accumulation and sleep are collected using the activPAL4 activity monitor (PAL Technologies Limited, Glasgow, UK; default settings) worn on the thigh and the Actigraph GT3X+ (Actigraph, Pensacola, FL, USA) worn on the wrist. Both devices are intended to be worn 24 h per day for 10 days. Eleven days before their clinic visit, the activPAL4 — previously initialised and waterproofed with a nitrile sleeve — and the GT3X+ monitor and wrist band are sent to participants along with instructions and materials for affixing and wearing both devices (transparent polyurethane acrylate adhesive patches and wrist band, respectively). It has been previously shown that participants attaching their activPAL devices is feasible, acceptable, and results in adequate placement on the anterior midline of the thigh [41]. A sleep diary is provided to participants for 10 consecutive days to define sleeping and waking periods. Incomplete records for sleep and wake times are inferred from using one or more published methods (visual estimation; automated estimation; average values, for example with, Edwardson et al. [42]; Winkler et al. [43]; LaCroix et al. [44] respectively) and checked against movement visually.

The primary outcome of total daily sitting time (sitting or lying while awake and wearing the device, h/16 h day) will be derived from the activPAL4 data, as will secondary measures of physical activity, standing time, and sedentary behaviour accumulation. The activPAL4 uses triaxial accelerometry (30 Hz) and activPAL proprietary algorithms (here, VANE) to measure sitting/lying, standing, and stepping, stepping cadence, as well as transitions between sitting/lying and upright posture with high accuracy, reliability and excellent responsiveness to change for sedentary reduction interventions [45–48]. Further measures, with less published data on validity, are available using the CREA algorithm [49]. Relative energy expenditure will be estimated as metabolic equivalents (MET) from cadence [50] or acceleration [51] with a similar level of accuracy to most accelerometers. Consistent with standard procedures for the field [42], sleep time and device non-wear time (based on the daily log and device non-movement) will be excluded from measures of physical activity and sedentary behavior, as will days with insufficient wear. Sufficient-wear criteria for waking days will be 10h hours wear while awake, with evidence of movement (≥500 steps/day and not ≥95% of the day in any one activity). The activPAL data (activPAL VANE algorithm primarily) are processed in SAS (9.4 or higher) using a bespoke program adapted from previous studies [52]. Measures are extracted overall and for specific timeframes of interest identified from the log — work and non-work days, work and non-work times, and time at the workplace — and as detailed time-series data (daily; hourly).

The wrist-worn Actigraph GT3X+ activity monitor is used to estimate sleep duration (i.e., total sleep time) and sleep quality metrics (i..e, sleep efficiency, wakefulness after sleep onset, sleep onset latency) and also enables harmonisation of data to other studies that have used wrist-worn devices [53, 54]. Actigraph GT3X+ data are sampled at 30 hz. Behaviour classification from the raw wrist-worn accelerometer data is performed in GGIR, an open source R package [55]. This package implements methods providing valid and reliable estimates sleep duration and quality [56] as well as physical activity [57, 58] from wrist-worn devices. The 24-h wrist monitor data will be separated into sleep periods (for measuring sleep quality and duration) versus waking periods (for other measures) based on sleep and wake times collected in the online-administered daily logs. Sleep quality and duration measures exclude sleep periods during which the device is not worn, which will be identified via the Choi algorithm [59].

#### Clinical assessment

Clinic visits occur at every data collection timepoint, wth the exception being at 15 months. Prior to each visit, participants are reminded to abstain from engaging in any moderate- to vigorous-intensity physical activity, from consuming caffeine and alcohol in the 24 h prior to the assessment, to fast for at least 8 h, and to take their medications for the day of the visit at lunchtime during the visit rather than in the morning. For each clinic visit, participants report to the Baker Institute Clinic at 8 am. Over a 5-h period, participants have their body weight, body composition, blood pressure and vascular function assessed, a fasting blood collection, and undergo a 2 h oral glucose tolerance test (OGTT) during which they complete their questionnaires*.* Upon completion of the OGTT, participants receive lunch (e.g., sandwich/coffee) followed by cognitive assessments. Those participants whose assessment occurs during COVID-19 related social isolation mandates are emailed the questionnaires and referred to off-site pathology for glycosylated haemoglobin (HbA1c) testing, all other measures are omitted.

Clinic measures and their procedures are described below.

#### Cardiometabolic outcomes

A peripheral intravenous catheter is inserted near the antecubital fossa and a fasting blood sample is taken to measure glucose, serum insulin, HbA1c, high-sensitivity C-reactive protein (hs-CRP), full blood examination (FBE), cholesterol, and triglycerides. Thereafter, the participant is guided through a standard 75-g 2 h OGTT [60], with plasma glucose and serum insulin collected at half-hourly intervals. An additional volume of blood is collected for verification of results if required. Further methodological information is available in Table 4.

**Table 4:** Brain health, cardiometabolic biomarkers and more comprehensive methodology descriptions

Cardiometabolic biomarker	Measurement methodology
Glucose	Spectrophotometric-hexokinase method using the Abbott Alinity Analyser
Insulin	Chemiluminescent Microparticle Immunoassay using the Abbott Alinity Analyser
Total cholesterol	Standard enzymatic methods using the Abbott Alinity Analyser
HDL	Accelerator Selective Detergent using the Abbott Alinity Analyser
LDL	LDL will be calculated using the Friedewald formula using the Abbott Alinity Analyser
Hs-CRP	Immunioturbidimetric method using the Abbott Alinity Analyser
HbA1c	Boranate Affinity HPLC method using the Trinity Premier Hb9210.
Full blood examination (FBE)	Beckman Coulter DXH800 instrument
TNF-α, IL-6, IL-1B, adiponectin, and leptin, BDNF,	MAGPIX multiplex microsphere-based immunoassay (Millipore, Billerica, MA)
IGF-1	Human IGF-I/IGF-1 Quantikine ELISA (R&D Systems)
Apolipoprotein E 4 (ApoE-4)	Stored in PAXgene DNA tube, PAXgene Blood DNA kits (QIAGEN) used for DNA isolation, PCR will be performed using custom design primers for the amplification of a segment of the ApoE gene and the 96-well Taqman Gene Expression qPCR Assay (Thermofisher Scientific). ApoE Sequencing (of the PCR product will occur using two single nucleotide polymorphisms (SNPs). Validation of sequencing results will be completed by targeted genotyping via differential amplification of ε2, ε3, or ε3 alleles with custom designed primer pairs.
Serum and plasma analyses	In preparation for serum analyses, samples will be collected in SST tubes and, for plasma analysis, in EDTA tubes. Both samples are rested for thirty minutes in the fridge, prior to centrifuging at 2000 RPM for 15 min at 4 degrees, with the separated plasma and serum aliquoted into 400ul and 750ul volumes and stored at −80 degrees celcius.

#### Inflammatory and neurotrophic factors

Markers of inflammation (e.g. hs-CRP, TNF-α, IL-6, IL-1β, adiponectin, and leptin) and neurotrophic factors (BDNF, IGF-1) will be ascertained from serum/plasma samples collected at all clinical assessments except the 3 month assessment. Samples will be analysed using multiplex microsphere-based immunoassays (MAGPIX, Merck Millipore) and traditional ELISA methods (R&D Quantikine) at the Sunshine Coast Health Institute. Apolipoprotein E 4 (ApoE-4), a commonly assessed genetic risk marker for cognitive decline is assessed at baseline only from DNA isolated from blood collected into PAXgene® tubes using QIAGEN DNA extraction kits. Custom-designed primers will be used for PCR amplification of the ApoE gene (Taqman qPCR,Thermofisher Scientific). Refer to Table 4 for further detail on inflammatory and neurotrophic factor methodologies.

#### Cognitive function

Global cognitive function is first screened with the Addenbrooke Cognitive Examination-Revised (ACE-R) and then assessed using the Cambridge Neuropsychological Test Automated Battery (CANTAB) [61]. The ACE-R is administered by the research staff at baseline only, as it provides a global measure of cognition. ACE-R evaluates six cognitive domains (orientation, attention, memory, verbal fluency, language, and visuospatial ability) and includes an inbuilt Mini Mental State Exam [62].

**Fig. 3:**
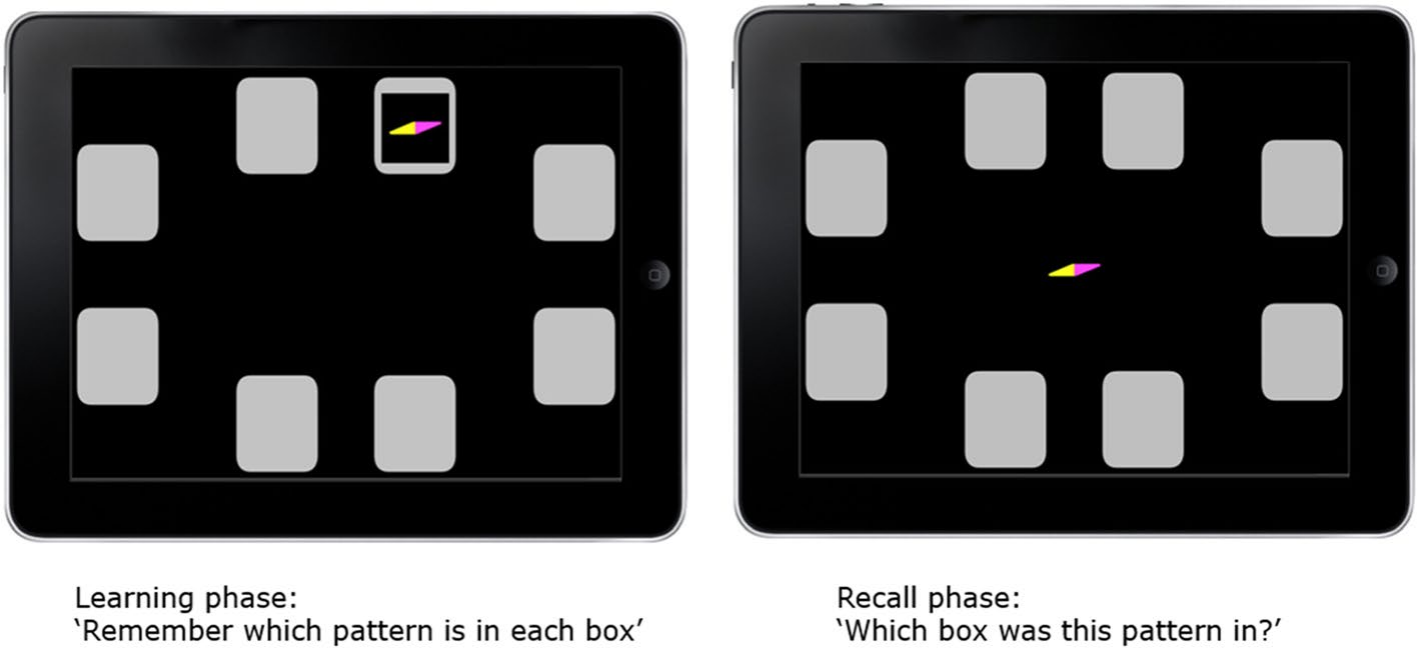
Paired Associates Learning task in CANTAB [63]. Participants are shown boxes on the screen containing specific patterns which are “opened” in a randomised order. The patterns are then displayed in the middle of the screen (as pictured) one at a time and the participant must locate which box the designated pattern is in. If the participant makes an error, the boxes are revealed in sequence again and the participant retries the task [64]. Paired Associates Learning task adjusted (PAL TEA) is a primary outcome

Following the ACE-R test, CANTAB is completed by the participant at baseline, and then tested additionally at the 6, 12 and 18 month assessments. Specific CANTAB tests (and the domain they measure) are: Paired Associates Learning (visual memory and new learning); Reaction Time (motor and mental response speeds, as well as measures of movement time, reaction time, response accuracy and impulsivity); Delayed Matching to Sample (visual matching ability and short-term visual recognition memory); and, Spatial Working Memory (strategy and working memory). The Motor Screening Task is included as an introduction to the CANTAB assessments. The key primary outcome is the total number of errors on the Paired Associates Learning task adjusted (PAL TEA) shown in Fig. 3. The outcome is automatically calculated by CANTAB. The PAL TEA score represents the number of times a participant chose the incorrect option during the task. Participants that terminate the task early have their scores adjusted; accounting for the estimated number of errors they would have made on all the problems. This allows for comparison with those who completed the final stage of the task. The CANTAB is an iPad administered cognitive battery and results are downloaded from the official Cambridge Cognition platform.

#### Anthropometry: height, weight, waist and hip circumferences and body composition

Height is obtained using a stadiometer (Seca, Germany) at the baseline clinic visit only, measured in duplicate, with a third measurement taken if the difference between the first two measurements is ≥0.5 cm. Body mass is measured using a platform scale (JAC-929; Nuweigh, Australia) taking measures in duplicate, with the average of two reading recorded at each assessment. BMI is calculated using height and weight measurements (weight / height^2^). Waist circumference is obtained using a non-elastic tape measure around the midpoint between the iliac crest and the lowest rib. Hip circumference is measured at the maximum circumference in the horizontal plane, over the buttocks. Both measurements are recorded according to the nearest 0.1 cm, and completed twice unless the first two differ by ≥1 cm, in which case a third measure is obtained. A whole-body dual energy x-ray absorptiometry (DXA) scan (Lunar iDXA; GE Healthcare, Australia) is used to assess body composition variables, including total and regional fat mass, and lean mass, and total body fat percentage. Visceral adipose tissue (volume and mass) is estimated by the DXA software using the android region of the body. Where participants’ body size exceeds the DXA scan area, a half body scan is performed, which has previously been shown to provide valid estimate of body composition [65]. Where possible, the same research staff member will perform each participant’s follow-up scan.

#### Blood pressure

Blood pressure is measured via a digital blood pressure monitor (OMRON HEM-907; Omron Healthcare, Japan) placed on the non-dominant arm. Participants are requested to lie down in a dimly lit, temperature controlled (approximately 22–24 C) room. After 10 min of rest, the monitor takes three measurements with an interval of 2 minutes between each measurement. The monitor measures systolic pressure, diastolic pressure, and heart rate and determines the average of the three measurements separately. The three measurements, as well as the average values, are recorded.

#### Arterial function

Arterial function is assessed by flow-mediated dilation (FMD). Participants lie in a supine position in a dimly lit temperature controlled room for at least 20 min before the baseline ‘steady state’ recording of FMD is obtained. Recording is made on the brachial artery of the dominant arm (opposite to blood pressure) using a high-resolution ultrasound machine (Terason t3200, Teratech, Burlington, MA). A rapid inflatable cuff (SC-12-D, D.E. Hokanson Inc., Bellevue, WA) is placed distally to the antecubital fossa and the ultrasound probe applied to the brachial artery. After an optimal image of the brachial artery has been established, a 1 min recording of continuous resting vessel diameter and blood velocity (shear rate) is collected. The cuff is then inflated to > 200 mmHg for 5 min. Thereafter, the cuff is released to induce reactive hyperemia. An additional 3 min of ultrasound recording is completed to determine the post-deflation peak vessel diameter, which is compared to resting vessel diameter (FMD%).

#### Questionnaire measures

Surveys are administered either during the clinic visit, or emailed to the participants for self-completion. All surveys, unless specified otherwise, are combined together into one survey, referred to hereon as the OPTIMISE survey. Contingent on COVID-19 and associated social distancing mandates, all surveys are administered electronically. Information pertaining to questionnaire source, rationale for inclusion, reliability and validity is described below and summarised in Additional file 9.

#### Socio-demographic characteristics

Age and gender are ascertained in the eligibility survey. Sociodemographic characteristics (ethnicity, household type, education, occupation) are obtained at the baseline visit, with question formats based upon previous studies such as the Australian Diabetes, Obesity and Lifestyle (AusDiab) study [66]. Changes in occupation are recorded in follow-up assessments.

#### Health history checklist

A health history checklist recording pre-existing health conditions is recorded by research staff with participants at baseline, and updated throughout the trial in the instance it changes. The checklist includes questions pertaining to any history of angina, heart attack, heart bypass operation, stroke, angioplasty for peripheral vascular disease, kidney damage from diabetes, eye or retinal damage from diabetes, nerve damage from diabetes, numbness burning or tingling in feet, foot ulcer, and lower limb amputation.

#### Self-reported physical activity, sitting time, and sleep

The Active Australia questionnaire is used to measure the number of minutes per week engaged in walking, vigorous gardening, moderate activity, vigorous activity (time multiplied by two), and strength training, with amounts totalled to determine MVPA [67]. The IPAQ questionnaire (two-items) is used to measure overall sitting time obtained in hours and minutes across week days and weekends. Sitting time is contextualised as part of work, travel, recreation, on a screen device, online chores (e.g. emails), or ‘other’ sitting that occurs during waking hours [68]. Sitting time and activity are also assessed using the Sedentary, Transport and Activity Questionnaire (STAQ) [69], which assesses time spent in transport, whether driving, on public transport, walking, and cycling. STAQ has acceptable reliability and validity [69]. Sitting and activity are further quantified using the Occupational Sitting and Physical Activity Questionnaire (OSPAQ) [70]. Here, participants are asked to divide their work day into percentages spent sitting, standing, stepping, and performing heavy labour tasks. OSPAQ has demonstrated acceptable validity and reliability [70, 71]. Additional questions asked include the percentage of the work day that is occupied by prolonged sitting (sitting bouts equal to or greater than 30 min) is also obtained [72], as well as the proportion of the workday spent sitting in common occupational tasks [73]. Participants are asked to identify their desired levels of sitting, standing, and stepping during work and home hours [74]. A 15-item checklist is used to assess participants’ current use of sit less and move more strategies (at home and at work) and a 6-item questionnaire assesses barriers to sitting less and moving more [73]. Knowledge (5-items) and perceived organisational norms (4-items) about sitting, activity, and health are assessed using a questionnaire adapted from a previous trial [75]. Participant’s self-regulation strategies for sitting less and moving more are both assessed with 9-item questionnaires, which are adapted from previous trials [76]. Participants are asked to evaluate how supportive their peers are of reducing sedentary behaviour, as well as engaging in physical activity [77, 78]. Finally, participants are assessed for their perceived changes across 16 domains of sitting in the last 6 months (assessed at 6, 12 and 18 months only). This measure has been adapted from previous research for this trial [79].

#### Sleep diary

Each morning over a 10-day monitoring period (coinciding with the wear of the activity monitor devices), participants are emailed links to a REDCap administered online daily log. Participants can also request to use a handwritten copy of the diary. Using a modified version of the Consensus Sleep Diary [80], the log enquires about their wake (and out of bed) times that morning, their sleep times and sleep quality the night before (into bed, lights out, time to fall asleep, number and duration of awakenings), and over the previous day, their work hours and work location (workplace / home / other), whether each device was worn that day (yes/no) and the start and end times of any removal greater than 10 min. The consensus sleep diary is a validated measure of sleep [81].

#### Anxiety and depression

The Hospital Anxiety and Depression Scale (HADS) [82] is used to measure anxiety and depression. It is a valid and widely accepted measure for determining level of anxiety and depression and can reliably differentiate between the two [83]. The questionnaire consists of 14 items: seven for anxiety (e.g. “I feel tense or wound up”), and seven for depression (e.g. “I feel cheerful”).

#### Managing diabetes

The Problem Areas in Diabetes (PAID) [84] scale assesses perceptions of the intervention and its influence on subjective diabetes management. The measure has demonstrated high reliability [85] and sensitivity to change over time [84], and has demonstrated use for screening depression and emotional problems in people with diabetes [86]. The 5-point scale asks participants to rank aspects of their diabetes management from “Not a problem” to “Serious problem”. Questions pertain to feelings about diabetes treatment and treatment goals, diabetes and social situations, diabetes and adverse events, burden and acceptance of diabetes, and coping with diabetes complications.

#### Fatigue

Fatigue is evaluated with a Fatigue Symptom Inventory (FSI) [87]. The inventory questions the participant on their current and previous level of fatigue in the last week. It examines how their fatigue interferes with activities of daily living, cognition, relationships, and mood. Participants are asked to rate the level of fatigue on a 0 (no fatigue) to 10 (extreme fatigue) scale. The measure has demonstrated validity with other fatigue symptom measures [87, 88].

#### Musculoskeletal pain and health

Musculoskeletal pain and health is measured using the 27-item modified Nordic Musculoskeletal Questionnaire, which surveys both the last 3 months and the last 7 days [89]. Participants indicate the body locations they have experienced ‘trouble’ in muscles or joints. Pain is ranked with an adapted 0–10 scale [90], where 0 indicates ‘no pain’, and 10 indicates the ‘worst pain imaginable’. This measure has been demonstrated to be repeatable and sensitive to change [91].

#### Work

Participants are asked to estimate their hours and days working, and how many leave days they have had in the preceding 3 months due to illness. Perceived workload and caring responsibility are assessed with a valid [92] two-item questionnaire based on the Borg workload scale [93], and NASA Task Load Index [94]. Self-reported work satisfaction [95] and work performance [96] are also assessed via 7-point scales. Participants are surveyed for how they feel during work with the ultra-short measure for work engagement (UWES-3) [97] featuring 3-items with a 7 point scale, including energy levels, enthusiasm, and being immersed in their work. The Work Limitations Questionnaire (WLQ) [98] is used to examine the frequency of difficulty that the participant has with performing specific work-related tasks. The WLQ has demonstated validity and reliability and is correlated with arthritis pain, as well as functional limitations and work productivity [98].

#### Workplace environment

An audit of the participant’s work environment is conducted at baseline only and made according to the Checklist of Health Promotion and Environments at Worksites (CHEW) [99] which was successfully modified for sedentary behaviour interventions in BeUpstanding [100]. Information is captured on layout, the nearby physical environment (e.g. stairs, centrally located printers and amenities), and workplace policy (such as flexible work hours). The 26-item questionnaire also includes questions pertaining to whether the workplace provides sit-stand desks, and whether a wearable tracker has been previously provided by employers.

#### Medication and allied health appointments

A record of the participant’s medication, including dosage, frequency is created at the baseline clinical assessment. Any changes to their medication is noted at each subsequent assessment. Similarly, any appointments with an allied health professional in the preceding months between clinical visits are recorded and updated at each assessment. Both records inform potential confounding effects of medication and allied health treatment on the primary and secondary analyses.

#### Personal wearables questionnaire

Participants use of wearable activity trackers and/or apps in the preceding months is recorded at each visit. Questions about type of wearable and app have been adapted from previous research [101], with the inclusion of additional questions pertaining to how long they have been using the device for, what feature they use (e.g., step counts, exercise intensity), how often they follow associated prompts, and whether these are followed at work, home, or both. Similar questions have been used in previous workplace sedentary behaviour interventions [101] and are important for evaluating prior exposure to health-behaviour trackers.

#### Dietary intake

Dietary intake is measured using the University of Newcastle’s Australian Eating Survey (AES), a Food Frequency Questionnaire which examines eating habits over the previous 3–6 months [102]. It is a semi-quantitative questionnaire of 120 items. The questionnaire has comprehensive validaty and reliability [103], requires less time to complete compared to a 24-h dietary recall, and is representative of longer term changes in dietary intake. Completion of the questionnaire results in a report detailing total daily energy intake, the contribution of healthy nutrient-rich and unhealthy nutrient-poor food choice to diet, the Australian Recommended Food Score (ARFS) a diet quality score indicative of the participant’s alignment to Australian dietary recommendations, macronutrient intake, micronutrient intake and fibre intake. The results are considered as confounding variables on primary and secondary analyses.

#### Quality of life

Quality of life is measured using the Australian Quality of Life Survey (AQoL-8D) consisting of eight dimensions (Independent Living, Happiness, Mental Health, Coping, Relationships, Self Worth, Pain, Senses) totaling 35 items [104] . The questionnaire is validated and has high test-retest reliability [105].

#### Motivation for physical activity and motivation to break up sedentary behaviour

Motivation for physical activity and motivation to break up sedentary behaviour are assessed using two separate modified versions of the validated Behavioural Regulation Exercise Questionnaire (BREQ-3) [106] based on self-determination theory [107]. The original prompt of “Why do you engage in exercise?” has been modified for the two questionnaires separately as “Why do you engage or not engage in physical activity?” and “Why do you break up sitting with standing up and/or moving more versus continued sitting?” respectively. These two questionnaires contain 24 items each with a five-point Likert scale. There are six subscales that represent the average scores for 4 items: amotivation (lack of intention), four subscales reflecting degrees of extrinsic motivation - external regulation, introjected regulation, identified regulation, integrated regulation - and intrinsic regulation. This is the first known study to measure motivation to break up sitting time using a modified version of the BREQ-3 questionnaire.

#### Menopause status questionnaire

The menopause status questionnaire asks questions to assist in classifying menopause status according to the Stages of Reproductive Aging Workshop (STRAW+ 10) criteria [108]. It is administered only to those participants identifying as female. Participants answer questions pertaining to criteria that fall outside of the STRAW+ 10 including polycystic ovarian syndrome, premature ovarian failure, hypothalamic amenorrhea, oestrogenic malignancy, or endrometriosis. To determine menopause status, participants are asked when (if at all) they had their first menstrual period, and whether they have either temporarily stopped, or finished having their period. Menstrual cycle history, use of hormonal contraceptive or hormonal therapy, and associated symptoms (according to Greene Clinimetric scale) [109] are recorded.

#### Experience in the OPTIMISE your health intervention

At the 6, and 12 month assessments for the intervention group, and at the 18 month assessment for the delayed intervention group, questions are included pertaining to the participants’ experience in the intervention. These questions are primarily adapted from previous research [110] and related to overall experience, as well as experience with the specific components of the intervention. Participants are asked about how the intervention has led to changes in their activities of daily living, and whether the trial changed these activities on a scale of a lot, a little, or no change. The components of the trial are ranked from most important to least important according to their perceived utility to assist with making changes in the domains of sitting less and moving more. Finally, participants are asked about their goal setting, and whether it was realistic and achievable and supported by the research team, as well as the applicability of the intervention to people without diabetes in order to inform the generalisability of the intervention messaging.

#### COVID-19 impact questionnaires

A questionnaire was originally added to assess the immediate impact of the pandemic and restrictions (Additional file 8). The questionnaire records any changes that the participant incurred with respect to workload, work environment, caring responsibilities, sitting and standing at their workstation, joint and muscle discomfort, motivations to sit less and move more, and physical activity participation changes due to the pandemic and restrictions. Following the temporary ending of restrictions and return of the trial in October 2020, this questionnaire was replaced with a five-item questionnaire. The new questionnaire asks on a ten point scale (not at all – very much) how the pandemic impacts self-management of diabetes, sitting at desired levels, moving at desired levels, and participation in the trial. Finally, participants are asked how the pandemic has changed their work commute.

### Adverse events

Any adverse events encountered during the trial are recorded by the research staff when they arise. Adverse health events may lead to a pause in participation or withdrawal from the study. Information collected includes the type of adverse event, the date of onset and resolution (if applicable), the maximum intensity of the adverse event (mild, moderate, severe), action taken, the adjusted medication due to event, the likelihood of relationship to the trial (scale of 1–4; 1 indicating unrelated; 4 indicating definitely related), and whether the event has resolved. Adverse events are reported to Baker Governance and Alfred Ethics Committee (Additional file 10). The participant that reports the adverse event is followed up with a phone call in order to monitor their health and participation in the trial.

### Process evaluation

A process evaluation is undertaken for the intervention and its components by assessing direct implementation indicators and surveying participants at the end of the intensive (6 months) and extended phase (12 months) timepoints for the intervention group and at 18 months for the delayed intervention group. Elements of context (e.g. workplace support), implementation (e.g., number and duration of health coaching sessions), and mechanisms of impact (including potential mediators) are explored in accordance with the Medical Research Council process evaluation framework [111]. Process evaluation for the intervention components is summarised in Table 5.

**Table 5:** Data collected in OPTIMISE Your Health intervention participants for process evaluation

Component	Data collected	Collected
Health coaching: participation and selections	Danger zones for sitting	Sessions 1 & 7
	Strategies to sit less at work selected by participant (select any number of 12 listed + 2 ‘other’)	Sessions 1–10
	Strategies to sit move more at work selected by participant (select any number 17 listed + 2 ‘other’)	Sessions 1–10
	Strategies to sit less across the day selected by participant (select any number 12 listed + 2 ‘other’	Sessions 1–10
	Strategies to move across the day selected / continued (select any number 8 listed + 2 ‘other’)	Sessions 1–10
	Goal setting - smart goals (sit less across the day, sit less at work, move more at work, move more across the day)	Sessions 1–10
	Stand more at desk goal	Sessions 1–10
	Active breaks goal (encouraged 1 break per hour)	Sessions 1–10
	Daily steps goal	Sessions 1–10
	Readiness level (1–10) to change sitting and moving habits	Sessions 1 & 7
	Anticipated barriers to goal achievement	Sessions 1–10
	General review of strategies / goals (open text) including barriers, ease/difficulty & modifications	Sessions 2–10
	Number of coaching sessions completed (0–2 face to face; 0–8 telephone)	Sessions 1–10
OPTIMISE-extended health coaching: participation and selections	Two danger zones for sitting (1 & 2) selected by coach from 3 listed + other^a^	Tailoring session 1 & 2
	One strategy to sit less per danger zone selected by coach from 32 listed + other^a^	Tailoring session 1 & 2
	Two strategies to move more (1 & 2) selected by coach from 17 listed and other^a^	Tailoring session 1 & 2
	Active breaks goal set by participant (n active breaks/ day, 1–24)	Session 10 & Tailoring 2
	Daily steps goal set by participant (n steps / day, 5000–20,000)	Session 10 & Tailoring 2
	Number of texts sent (total, by week, by type), number of text messages replied to by participants (total, by week), content of participant replies.	propelo
Fitbit (Fitabase)	Usage (wear days, non-wear days)	Continuous from Session 1 to end of study
	Physical activity / sedentary behaviour (step counts, estimated energy expenditure, time spent in LPA, MPA, VPA, inctive behaviour), physical activity events autodetected & logged (e.g., ‘weights’, ‘walk’)	
	Heart rate	
	Sleep & sleep quality (Total sleep time duration, duration of each sleep stage, sleep onset latency, sleep efficiency, number and duration of wakes after sleep onset, restlessness count and durations)	
Intervention fidelity	Health coaching participation (n sessions completed)	Sessions 1–10
	Extended care participation (n text messages received)	Propelo
	Fitbit usage (% of days in intervention wore Fitbit)	Fitbit
	Sit-stand workstation in standing postion (not used / some days / most days / every day)	OPTIMISE survey 6 M & 12 M
COVID-19	Impact of COVID-19 pandemic on abilty to participate in OPTIMISE program (0–10); 1 item	OPTIMISE survey 6 M, 12 M & 18 M
Intervention satisfaction	Overall satisfaction (1–10) with the OPTIMISE intervention / the text message health coaching: satisfaction level, whether would recommend, usefulness (3 items)	OPTIMISE survey 6 M & 12 M
	Rating of each intervention element (1–10) over last 6 months: coaching (4 items; 6 M); text message coaching (4 items; 12 M); feedback (7 items); sit-stand workstation (10 items); Fitbit (10 items); goal setting (4 items).	OPTIMISE survey 6 M & 12 M
	Appropriateness of text message frequency, from 1 (nowhere near enough) to 10 (far too many) with 5 = the right amount (1 item)	OPTIMISE survey 12 M
	Likes & suggestions for improvement (open text) re: the OPTIMISE intervention / the text message health coaching	OPTIMISE survey 6 M & 12 M
	Usefulness (1–10) over the last 6 months for behavior change, for sitting less and for moving more of the intervention elements: face to face coaching (6 M); telephone coaching (6 M); text message coaching (12 M); monitoring steps/sitting via coach feedback; goal setting; sit-stand workstation; Fitbit.	OPTIMISE survey 6 M & 12 M

^a^ Health coach selects based on participant’s prior selections at tailoring session 1 and then participant can revised at tailoring session 2

### Economic evaluation

Incremental cost-effectiveness analysis is to be undertaken to determine whether the intervention represents “value for money” compared to the “usual care” control. The economic evaluation is conducted from a limited societal perspective, using detailed pathway analysis to specify all relevant intervention activities and costs. A detailed accounting of resource use required to deliver the two arms in the trial is undertaken to allow accurate costing of the interventions. The limited societal perspective incorporates cost impacts on government as the provider of healthcare services, healthcare costs to individuals and costs to workplace organisations. The intervention is costed assuming it is in ‘steady state’ (i.e. excluding research-related costs).

Additionally, a within-trial economic evaluation is to be undertaken as a cost-consequence analysis, comparing the costs of the intervention with the primary outcome measures (e.g. cost per change in HbA1c) at 6 months and 12 months timepoints. A modelled economic evaluation is to be undertaken, extrapolating intervention costs and effects and extending the target population, time horizon and decision context. The model will be extrapolated to lifetime to incorporate benefits accruing to both T2D and dementia prevention. As 5 years is insufficient to observe outcomes of dementia in a prevention study, the intermediate outcomes of the trial (cognitive function, neurotrophic factors and inflammatory markers) will be incorporated.

into final outcomes for economic evaluation, e.g., dementia diagnosis, time to dementia.

Incremental health benefits, reported as quality-adjusted life years (QALYs) saved, and incremental healthcare cost offsets attributable to T2D prevention and dementia are reported. The commonly accepted cost-effectiveness threshold of AUD$50,000 per QALY saved will be used to determine cost-effectiveness over the lifetime. Utility is calculated from the Assessment of Quality of Life AQOL-8D using the Australian algorithm [112] and multiplied using the area under the curve method by the time in the trial to derive the QALYs.

Finally, an incremental cost-effectiveness ratio will be determined by calculating the difference in cost between the intervention and usual care, divided by the difference in QALYs between intervention and usual care.

### Statistics

#### Sample size

With an estimated 20% attrition based on our previous intervention trials, 2-tailed significance of 2.5% (correcting for two primary outcomes for the original OPTIMISE protocol), 125 participants per group (250 total) are required for 80% power to detect minimum differences of interest in HbA1c and sitting of 0.5% and 0.5 h/16 h-day, assuming standard deviations (SD) of 1.6 and 1.3 and a pre-post correlation (r) of 0.7 and 0.6 respectively. A 0.5% HbA1c decrease is clinically meaningful corresponding to to an approximate 10% reduction in diabetes-related mortality [7]. Assumptions concerning attrition, SD and r for primary and most secondary outcomes were based on Living Well with Diabetes [77], AusDiab [66] and Stand Up Victoria [76] studies. Recruitment projections indicate that a sample of 250 participants is feasible within the allocated timeframe. Recruitment will stop when the trial reaches either the required sample size or the maximum number of participants who can be recruited in the trial’s allotted recruitment timeframe, while complying with the unforeseeable restrictions and conditions related to the COVID-19 pandemic.

The sample size for the cognitive function outcomes are dictated by the original sample size calculations described above. From a sample size of 250, we anticipate 160 participants (80 in each arm) will complete the 12-month assessment. This sample size of 80 per group provides > 80% power (5% 2-tailed significance) to detect our minimum difference of interest in the primary outcome of visual memory (PAL TEA) at 12 months (1/3 SD, d = 0.33) assuming r = 0.7, based on a previous trial [113]. Changes in cognitive function have been detected in a similar sample with 145 participants participating in a 24-week RCT assessing pharmacotherapy to improve metabolic control. Minimum detectable differences will be recalculated if the actual sample size is lower than projected due to the potential impact of the COVID-19 pandemic.

#### Statistical analyses

Outcomes are all continuous and expected to be either normal or log-normal, in which case log-transformation will be used. Analyses will be performed in STATA version 15 or higher. Significance is set at *p* < 0.05 two-tailed (except for *p* < 0.025 when testing co-primary outcomes based on α = 0.025). To test the differences between groups in primary and secondary outcomes during (3 months), end of the intervention (OPTIMISE; 6 months) and end of the extended maintenance phase (12 months – OPTIMISE-extended) mixed models statistical analysis will be employed, accounting for repeated measures, adjusting for baseline values, randomisation strata, and potential confounders. Within-group changes for intervention participants occurring during the no health coaching contact phase from 12 to 18 months will be tested and compared to intervention phases. Within-group changes will also be tested in the delayed intervention participants receiving the OPTIMISED intervention after 12 months, at the 18 month timepoint, using paired t-tests (or non-parametric paired tests). Possible confounders will be identified a priori from the literature and narrowed down to a number that can be modelled without overfitting, based on an objective criterion not open to manipulation (backwards elimination: retaining age, sex, and *p* < 0.2 association with the outcome). Analyses will follow intention-to-treat principles. Sensitivity to handling missing data will be evaluated by comparing results from the main analyses (evaluable case analysis for mixed models; complete-case analysis for t-tests) with alternative methods that are appropriate for different missing data scenarios (e.g., multiple imputation; selection-covariate adjustment). Descriptive statistics will be used to describe the implementation indicators of the different phases of the intervention.

## Discussion

Adults with T2D have been shown to engage in higher levels of sedentary behaviour than those with normal glucose metabolism, and many undertake little or no physical activity. Regularly interrupting prolonged sedentary time has been demonstrated to improve cardiometabolic health [18], and increasing physical activity has the potential to improve glycaemic control [114]. Findings from earlier intervention trials have demonstrated the feasibility of reducing sedentary behaviour [115] and have shown modest changes to markers of cardiometabolic health in non-clinical groups [21]. However, no studies have specifically focused on the combination of “sitting less” and “moving more” in the context of type 2 diabetes management.

The Optimise Your Health trial will take advantage of a large sample that is powered to address multiple research questions. There will be an extensive follow-up process at short, medium, and long term with each timepoint encompassing a wide array of phenotyping. A multicomponent intervention will be deployed based on the extensive insights previously obtained from earlier sedentary behaviour interventions, extending to novel behavioural prompts and strategies to promote sitting less and moving more across different behavioural contexts. A modified version of the Optimise Your Health program (OPTIMISED) that is less resource intensive will be implemented and evaluated for feasibility and acceptability. This, in addition to a cost-effectiveness evaluation, will be critical to guiding future wider scale uptake.

Results from the trial will inform whether a sit less and move more intervention is effective in adults with type 2 diabetes. The findings have the potential to inform more prescriptive guidelines and behavioural strategies of benefit in a clinical management context. Overall this study will contribute extensively to the field of sedentary behavior research, build upon existing evidence and provide new insights on the merits of targeting sitting less and moving more as a therapeutic utility to improve health outcomes.

## Supplementary Information

**Additional file 1.** Research funding.

**Additional file 2.** Participant information and consent form.

**Additional file 3.** Inclusion and exclusion criteria.

**Additional file 4.** Intervention handbook.

**Additional file 5.** Example activity feedback report.

**Additional file 6.** Intervention strategy list.

**Additional file 7.** Health coaching script.

**Additional file 8.** COVID-19 Snapshot Questionnaire.

**Additional file 9.** Questionnaire measures.

**Additional file 10.** Adverse events report.

**Additional file 11.** SPIRIT checklist.

## Data Availability

Availability of the data from the OPTIMISE Your Health trial is subject to the approval of a formal application made to chief investigators on the project.

## Abbreviations

ApoE4: Apolipoprotein E4
AQoL: Assessment of Quality of Life
AusDiab: Australian Diabetes, Obesity and Lifestyle study
BCW: Behaviour Change Wheel
BDNF: Brain-derived neurotrophic factor
BMI: Body mass index
CANTAB: Cambridge Neuropsychological Test Automated Battery
COM-B: Capability, opportunity, motivation-behaviour
FBE: Full blood examination
HDL: High-density lipoprotein
hs-CRP: High-sensitivity C-reactive protein
iAUC: Incremental area under curve
IGF-1: Insulin-like growth factor 1
IL-1β: Interleukin 1 beta
IL-6: Interleukin 6
LDL: Low-density lipoprotein
MVPA: Moderate-to-vigorous intensity physical activity
NDSS: National Diabetes Services Scheme
NHMRC: National Health and Medical Research Council
OGTT: Oral glucose tolerance test
QALY: Quality adjusted life year
RCT: Randomised controlled trial
TNF-α: Tumour necrosis factor alpha
