# Supplementary material for: Sitting less and moving more for improved metabolic and brain health in type 2 diabetes: ‘OPTIMISE your health’ trial protocol
Source: BMC Public Health. 2022 May 10;22:929. doi: 10.1186/s12889-022-13123-x (PMC9086419; doi:10.1186/s12889-022-13123-x)
Supplement: Supplementary file 1 — Additional file 1. Research funding. [file 12889_2022_13123_MOESM1_ESM.docx]

According to chronological sequence of research grant attainment, the phases of the OPTIMISE Your Health trial and their respective grants include:

1. National Health and Medical Research Council (NHMRC) Project Grant – APP1139974: OPTIMISE Your Health (0 – 6 months). Commenced in 2018 and referred to as OPTIMISE.
2. NHMRC Boosting Dementia Project Grant – APP1171759: OPTIMISE Your Health extended care (0-12 months intervention; 12-18 months no-contact maintenance). Commenced in 2020, allowed extension of the original protocol from 6 months to 18 months.
3. Diabetes Australia Research Foundation: OPTIMISED (12 – 18 months for delayed intervention group only). Commenced in 2021 and referred to as OPTIMISED.
