# Supplementary material for: Sitting less and moving more for improved metabolic and brain health in type 2 diabetes: ‘OPTIMISE your health’ trial protocol
Source: BMC Public Health. 2022 May 10;22:929. doi: 10.1186/s12889-022-13123-x (PMC9086419; doi:10.1186/s12889-022-13123-x)
Supplement: Supplementary file 2 — Additional file 2. Participant information and consent form. [file 12889_2022_13123_MOESM2_ESM.docx]

**
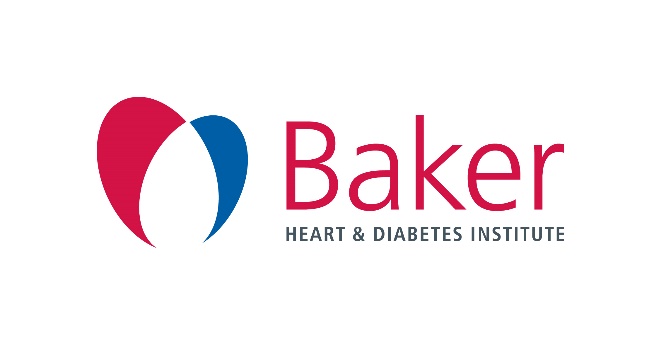
**

**Participant Information Sheet/Consent Form**

**Interventional Study** - *Adult providing own consent*

***Baker Heart and Diabetes Institute***

| **Title** | Office-based Program To Improve Metabolic control In Sedentary Employees with type 2 diabetes |
| --- | --- |
| **Short Title** | The ‘OPTIMISE Your Health’ Study |
| **Protocol Number** | V10.0 |
| **Project Sponsor** | Baker Heart and Diabetes Institute |
| **Principal Investigator** | Prof David Dunstan |
| **Associate Investigators**  **(Australia)** | Prof Neville Owen, Prof Elizabeth Eakin, Prof Stuart Biddle, A/Prof Genevieve Healy, Prof Robin Daly, Prof Daniel Green, Prof Marjory Moodie, Dr Elisabeth Winkler, A/Prof Neale Cohen, Ms Ruth Grigg, Ms Kym Rickards, Ms Frances Taylor, Miss Melanie Townsend, Mr Christian Brakenridge, Dr Francis Dzakpasu, Dr Paul Gardiner, Dr Mia Schaumberg, Dr Brianna Fjeldsoe, Prof Len Gray, A/Prof Tracy Comans, Dr Ana Goode, Dr Ashleigh Homer |
| **Associate Investigators (International)** | A/Prof Matthew Buman (USA) |
| **Location** | Physical Activity Laboratory, Baker Heart and Diabetes Institute, Melbourne, VIC. |

**Part 1 What does my participation involve?**

**1 Introduction**

You are invited to take part in this research project, the “**Office-based program to improve metabolic control in sedentary employees with type 2 diabetes: The 'OPTIMISE Your Health' study**.” This is because you have type 2 diabetes – a condition where too much glucose in the blood can increase your risk of developing diabetes complications, cardiovascular disease and dementia. As 1-in-20 adults in Australia have type 2 diabetes, you represent an important target group for evaluating strategies for disease prevention. This 18-month research project is investigating whether a multicomponent intervention designed to reduce sedentary time (too much sitting) improves blood glucose control and cognitive function compared to standard/usual care.

This Participant Information Sheet/Consent Form tells you about the research project. It explains the tests and treatments involved. Knowing what is involved will help you decide if you want to take part in the research.

Please read this information carefully. Ask questions about anything that you don’t understand or want to know more about. Before deciding whether or not to take part, you might want to talk about it with a relative, friend or your local doctor.

Participation in this research is voluntary. If you don’t wish to take part, you don’t have to. You will receive the best possible care whether or not you take part.

If you decide you want to take part in the research project, you will be asked to sign the consent section. By signing it you are telling us that you:

• Understand what you have read

• Consent to take part in the research project

• Consent to have the tests and treatments that are described

• Consent to the use of your personal and health information as described.

You will be given a copy of this Participant Information and Consent Form to keep.

This research project has been registered with the “Australian New Zealand Clinical Trial Registry” (ANZCTR): ACTRN12618001159246p

**2 What is the purpose of this research?**

It is well known that being physically active is important for maintaining good physical and mental health. However, new evidence shows that being sedentary (sitting for prolonged periods) is associated with elevated blood glucose and insulin – two important risk factors for cardiovascular disease. Interestingly, excessive daily sitting (of which 60% is accrued at the workplace) has been shown to increase the risk of developing cardiovascular disease (the most common complication observed in adults with type 2 diabetes) and also dementia. This highlights a need to explore alternative, practical, and low risk approaches to reduce sitting time across all spheres of daily living (i.e. work and home).

Recent evidence suggests that reducing and breaking up sitting time may be a suitable self-care behaviour to improve glucose control in type 2 diabetes. Our laboratory has recently shown in overweight/obese individuals and those with type 2 diabetes that regular (every 20-30 minutes, brief (2-3 minute) bouts of light-intensity activity (i.e. walking or simple resistance activities) lowers blood glucose levels across the day by 24-35% when compared to prolonged sitting. We’ve also shown that reducing and breaking up sitting can help lower blood pressure, improve measures of blood vessel health, and improve cognitive function. While these results are interesting, it remains to be determined whether there are potential long-term benefits of reducing and breaking up sitting in individuals with type 2 diabetes.

This study will test whether a 6-month multicomponent intervention (involving health coaching, sit-stand workstations at work, and a smartphone behavioural promoting tool) followed by 6-months of text message delivered support, can reduce daily sitting time and improve blood glucose control and cognitive function as well as risk markers for cardiovascular disease (blood pressure and blood vessel health). The results of this study will build on our understanding of the cardiometabolic consequences (i.e. risk of developing heart disease) of too much sitting in individuals with type 2 diabetes and will help design intervention strategies in the community to reduce the risk of developing complications associated with diabetes.

This research has been initiated by Professor David Dunstan and has been funded by two National Health and Medical Research Council (NHMRC) project grants and one grant from the Diabetes Australia Research Foundation. Part of the results of this research will be used by PhD students to obtain doctorate degrees. The PhD students will be supervised by the chief investigators of the NHMRC grants: Professor David Dunstan and Dr Paul Gardiner.

**3 What does participation in this research involve?**

Approximately 250 volunteers will be required for this study. Participation in this study will involve up to five visits to the Baker Heart and Diabetes Institute, over a period of 18 months. This study is broken down into the following steps:

**Step 1 – Consenting to the study**

A researcher will call to confirm that you have read this form and will be available to answer any questions you have about the study. Prior to signing the consent form, please feel free to speak with your family and treating doctor about participating in this research project. You will also need to obtain written permission from your employer to use a sit-stand workstation at your workplace. There are written information fact sheets available for your Employer to read. You will have up to 3 months to obtain your employer’s permission. If you would like to participate, we ask you to sign the consent form. There are two options for you to sign the consent form, either via hard-copy (located at the end of this document and returned via post) or electronically (via an email sent to you and returned automatically when complete). Please let one of the researchers know which one you would prefer.

**Step 2 – Preparation for baseline testing**

Before you start the study, we will measure how much time you normally spend across the various physical activity intensity levels (sedentary, light, moderate and vigorous activity). In order to do this, you will be sent (via post) two physical activity monitors – an accelerometer (worn on the wrist like a watch) and an inclinometer (worn on the thigh using non-allergenic patches). You will be requested to wear these monitors 24 hours a day for ten consecutive days. Additional patches for the inclinometer will be provided to change the dressings as required. During the ten days, we will ask you to complete a short (1-2 min) online questionnaire daily telling us your sleep times, and your work hours.

During the ten days whilst you are wearing the monitors, we will ask you to complete three online questionnaires. These questionnaires are sent to your email address whereby you open the link on a computer or your smartphone to complete the questionnaires. The first questionnaire is a ‘Quality of Life’ questionnaire and asks about how you are currently feeling. This takes 5-10 minutes to complete. The second questionnaire asks about your food and drink habits and takes 10-15 minutes to complete. . The third questionnaire asks about your motivation to increase your Physical Activity and takes 5-10 minutes to complete.

On the tenth day (i.e. the day before your first visit to Baker Institute), you will be asked to refrain from any moderate–vigorous physical activity (exercise), alcohol, and caffeine. This is because moderate–vigorous physical activity, alcohol, and caffeine can all effect the results of tests that we will perform during your testing day.

**Step 3 – Baseline testing (Visit 1 to Baker Institute)**

On your first testing day, you will report to the Baker Clinic in the morning (8am) having fasted for at least 8 hours. The two physical activity monitors will be collected from you when you first arrive. Over a 5-hour period, the following measures we be collected:

1**. Participant registration form:** You will be asked to complete a participant registration form that contains information about your contact details, next of kin details, local GP details, and medication use (medicine name, dose and dosage)

2. [**Anthropometric**](https://www.google.com.au/search?q=Anthropometric&spell=1&sa=X&ved=0ahUKEwiO2sKrqaLcAhUBVLwKHV80DxkQkeECCCYoAA) **and blood pressure:** Your weight, height, waist, and hip circumference and blood pressure will be assessed using standard techniques.

3. **Body fat/muscle scan:** This will be measured by a dual-energy x-ray absorptiometry (DXA) body scanner and will record body fat percentage, lean tissue, and bone mass. It is a non-invasive procedure whereby you lay on a bed below the scanner, which slowly travels along the length of your body to measure and record your body composition. The scan takes about 10 minutes to complete

4. **Blood vessel health:** We will assess how your blood vessels response to an increase in blood flow after occluding the main artery in your arm with a blood pressure cuff for a 5-minute period. This test, known as flow mediated dilatation (FMD), uses ultrasound to measure changes in the artery diameter before and after occlusion of blood flow.

5. **Blood samples:** After completing the above measures, a venous catheter will be inserted into your arm to collect blood at half-hourly intervals during a 2 hour 75-g oral glucose tolerance test (OGTT). The following measures will be assessed: glucose, serum insulin, hs-CRP, HbA1c, total cholesterol, triglycerides, and high-density lipoprotein cholesterol. A fasting blood sample will be collected for further research relating to Type 2 Diabetes and cognitive health, including markers of inflammation (e.g. IL1β, TNFα. IL-6), neurogenesis (e.g. brain-derived neurotrophic factor), and genetic risk of dementia (APOE-4 status).

6. **Questionnaires:** During the 2 hour OGTT, we will ask you to complete various questionnaires that contain questions such as your demographics, your sitting and activity behaviours, your work, neighbourhood and home environment, your mood and energy levels and about your health (past and present).

7. **Cognitive assessments:** Upon completion of the 2 hour OGTT, you will receive a meal (e.g. sandwich/fruit) prior to undergoing two cognitive assessments: 1) ‘cognitive practical assessment’ that involves completing various cognitive assessments (e.g. memory, attention, psychomotor control, executive function) on an iPad; and 2) ‘cognitive written assessment’ that is completed with the researcher and takes about 10-20 minutes to complete.

**Step 4 – Randomisation**

After the measures have been collected, you will be **randomised** to either the ‘intervention’ group or the ‘delayed intervention’ group. You have a 1-in-2 chance in being randomised to the intervention. Regardless of which group you are randomised to, you will be asked to repeat the majority of the measures listed in Step 2 and Step 3 at 3-months, 6-months, 12-months and 18-months. You will be reimbursed for the parking costs or public transport costs associated with your visits to the Baker Clinic.

***For those randomised to the intervention group:***

If you are randomised to the intervention group, you will begin the intervention within 2-3 weeks of your first visit at Baker. During the 2-3 weeks, we ask that you watch 3 very short videos (sent via email) that contain information about the study. The intervention consists of three components in the first 12 months:

***1) Tailored health coaching:*** The tailored health coaching involves 2 x face-to-face health coaching sessions and 8 x telephone sessions. The health sessions will cover topics about how to correctly use the sit-stand workstation provided to you (described in #2 below), feedback on your activity levels, strategies in reducing and breaking up sitting time at work and home, identifying health-related goals and how to use the activity wrist-worn tracker and app. The first face-to-face health coaching sessions will occur when you receive your sit-stand work station, and the second will occur during your 3-month assessment at the Baker Clinic. The telephone calls will occur weekly for the first three weeks, and then every three weeks thereafter. After the first 6 months you will continue to receive health coaching via targeted text message communications. You will receive approximately four text messages every week for 6 months. You will also receive a telephone call at 9 months

***2) Provision of a sit-stand desktop workstation to use at your workplace:*** Approximately 2-3 weeks after your first visit to Baker, we will deliver and install the sit-stand workstation to your workplace. The workstation weighs 22.5kg and is placed on top of your existing work surface. It allows you to easily and quietly alternate your working posture between sitting and standing while still working at your computer. At the end of the study, you will be able to keep your sit-stand workstation.

***3) Monitoring your activity levels:*** For the duration of the study, you will receive a Fitbit activity tracker (worn on the wrist) to use with the Fitbit app on your smartphone. This will allow you to monitor your activity levels and time spent not moving (e.g. sitting, lying). It will also allow you to set reminders to break up your sitting time. At the end of the study, you will be able to keep your Fitbit.

***For those randomised to the ‘delayed intervention’ group:***

If you are randomised to the delayed intervention group, you will go about your usual behaviours over the next 12 months. You will receive newsletters on advice for healthy living as well as follow-up phone calls by the researchers for any questions you have. At the end of the 12-month period, over the next 6 months, you will receive a modified version of the intervention, including the use of a sit-stand workstation at your workplace.


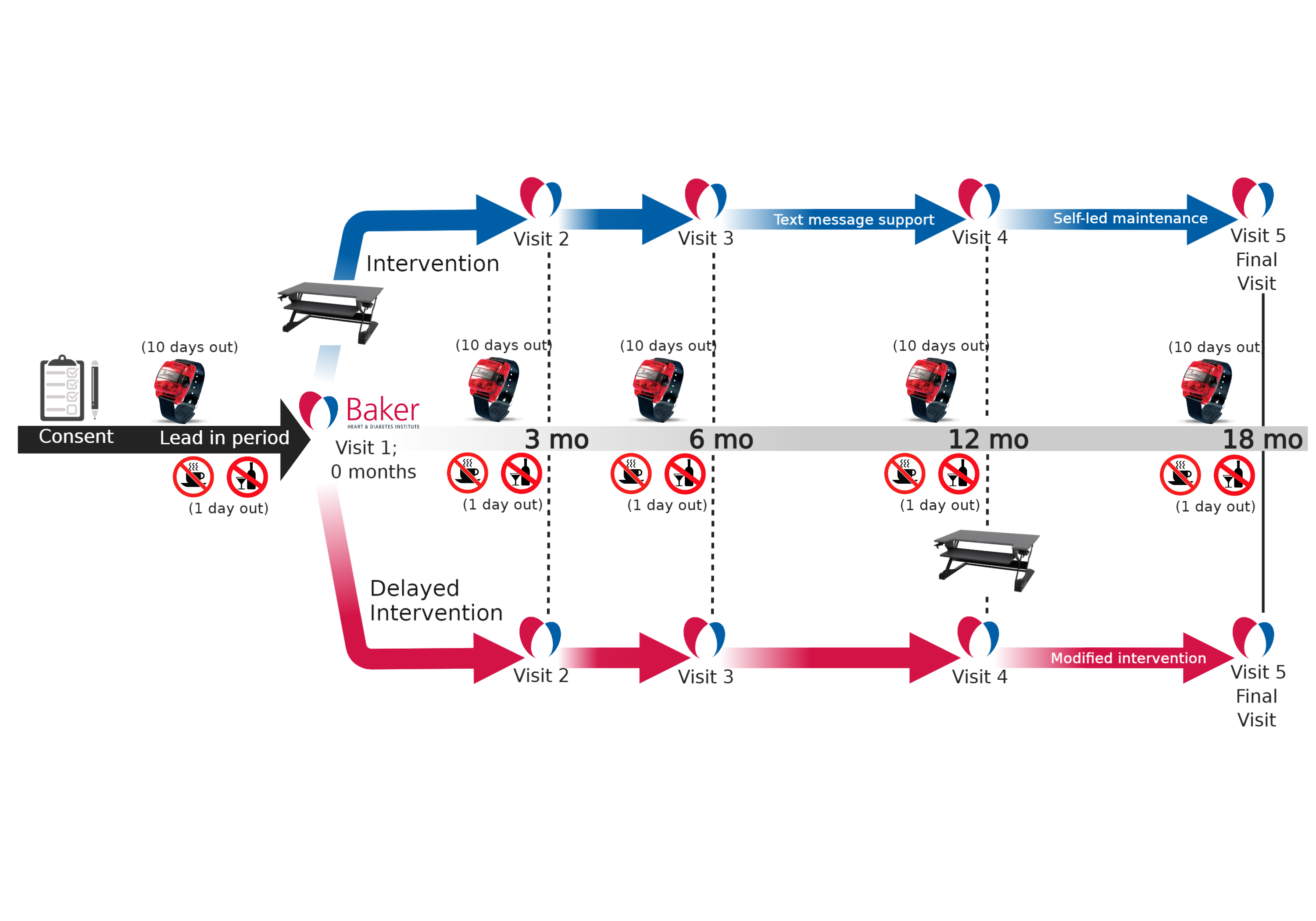


Figure 1 – Overview of study

**4 What do I have to do?**

In order to participate in the study, **all participants** must:

- Be willing to attend five scheduled visits over an 18 month period at the Baker Clinic and be willing to undergo the necessary tests.

- Wear two physical activity monitors (one on the wrist and one on the thigh) for ten consecutive days on five different occasions across the 18-month period.

- Restrict your moderate-vigorous physical activity and alcohol and caffeine intake the 24 hours prior to each assessment at the Baker Clinic.

Throughout the study, you will be able to take your regular medications. However, we do ask that you tell us if there are any changes to the type and dosage of your medications.

If you are randomised to the **intervention group**, you will be asked to:

- Adhere to the study intervention by using the sit-stand workstation during working hours with the aim of reducing your total sitting time and breaking up long periods of sitting.

- Participate in the face-to-face and telephone based health coaching sessions

- Regularly use the Fitbit activity watch and Fitbit App on your smartphone

- Outside of working hours we will provide further tips and strategies to reduce the time you spend sitting but request you to not begin any strenuous new physical activities while participating in the study.

If you are randomised to the **delayed intervention group**, you will be asked to go about your usual behaviours for the next 12 months. Thereafter, for the remaining 6 months you will be provided with a,modified version of the intervention, including the use of a sit-stand workstation.

**5 Other relevant information about the research project**

This project involves researchers from a number of universities working in collaboration. Baker Heart and Diabetes Institute is the primary organisation responsible for the project and all visits will conducted at the Baker Clinic. The other universities that are involved in this study are The University of Queensland, University of Southern Queensland, University of the Sunshine Coast, Deakin University (Victoria), University of Western Australian, and Arizona State University (USA).

**6 Do I have to take part in this research project?**

Participation in any research project is voluntary. If you do not wish to take part, you do not have to. If you decide to take part and later change your mind, you are free to withdraw from the project at any stage.

If you do decide to take part, you will be given this Participant Information and Consent Form to sign and you will be given a copy to keep.

Your decision whether to take part or not to take part, or to take part and then withdraw, will not affect your relationship with Baker Heart and Diabetes Institute

**7 What are the possible benefits of taking part?**

We cannot guarantee or promise that you will receive any benefits from participating in this study. We know that large amounts of uninterrupted sitting time are linked to poor health, so breaking up sitting time may lead to improved health. Previous workplace interventions that used sit/stand workstations or encouraged breaks in sitting have reported reductions in perceived musculoskeletal discomfort, eyestrain, tiredness and improved mood, with neutral or positive impacts on productivity (refer to *Reducing prolonged sitting in the workplace – VicHealth report*). However, we don’t know if such interventions can also lead to improvements in blood glucose control and risk makers of cardiovascular disease or cognitive function. If you do take part, you will receive feedback on your activities (sitting, standing, lying) as well as your physical and blood measures, and how these may have changed since the start of the study. This study will provide us with valuable information to develop and improve workplace health promotion programs. Therefore, your participation may help improve the health of office workers in the future.

**8 What are the possible risks and disadvantages of taking part?**

**Possible side effects**

This research involves a number of procedures in which you may have none, some or all of the effects listed below, and they may be mild, moderate or severe. If you experience any of the side effects, or are worried about them, talk with your study doctor. Your study doctor will also be looking out for these possible side effects. There may be side effects that the researchers do not expect or do not know about and that may be serious. Tell your study doctor immediately about any new or unusual symptoms. Many side effects go away following the completion of a procedure. However, sometimes side effects can be serious, long lasting or permanent. A member of the research team will discuss the best way of managing any side effects with you.

The risks associated with the individual procedures involved in the study are as follows:

**1. Cannulation / blood test** – Having a blood sample taken may cause some discomfort or bruising. Sometimes, the blood vessel may swell, or blood may clot in the blood vessel, or the spot from which tissue is taken could become inflamed. Some people may feel faint when having blood taken, and may occasionally faint. Rarely, there can be a minor infection or bleeding. If this happens, it can be easily treated.

**2. Skin irritations** – In the unlikely event, participants may develop a skin irritation from the tape used to keep the thigh monitor in position. An alternative tape can be supplied to you if this occurs,

**3. Disclosing personal information** – This study involves disclosing personal information such as your medication use and type 2 diabetes history. Although all information will remain confidential, some participants may experience some mild anxiety/distress while answering these questions.

**4. Vascular function assessments** – Mild discomfort (e.g. pins and needles) may arise during the inflation of blood pressure cuffs on the right leg during the vascular function assessments (e.g. ‘the FMD test’). The longest period of cuff inflation will be five minutes. Cuffs will not be inflated to such an extent or duration that will cause permanent damage to your arms and leg.

**5. Body composition (DXA) assessment –** This is a 10-minute non-invasive medical test which involves exposure to a very small amount of radiation. As part of everyday living, everyone is exposed to naturally occurring background radiation and receives a dose of about 2 millisieverts (mSv) each year. The effective dose from this study is about 0.02 mSv. At this dose level, no harmful effects of radiation have been demonstrated as any effect is too small to measure. The risk is believed to be minimal. The procedure involves lying on a table fully clothed and having a scanner move up and down to compile a full picture of your body. **Have you been involved in any other research studies that involve radiation?** If so, please tell us. Please keep information contained within the Patient Information and Consent Form about your exposure to radiation in this study, including the radiation dose, for at least five years. You will be required to provide this information to researchers of any future research projects involving exposure to radiation.

**6. Using the sit-stand desktop workstation** – Some initial discomfort may be experienced from using the sit-stand desktop workstation as a result of standing more. Additionally, there is a small possibility that you may experience minor and transient muscle soreness following the suggested active breaks.

There may be additional unforeseen or unknown risks. Participation in this study can be suspended or terminated if a medical issue or distress occurs. If you have a regular local doctor, it is desirable that they be advised of your decision to participate in this research study. If you do have a local doctor, by signing this consent form, you are agreeing to inform him/her of your participation in the study.

**Finding unknown abnormal results**

During the screening process and assessment days, there is a possibility that your results will uncover an abnormal test result or medical condition. If an abnormal test result or medical condition comes to light during the study, the project coordinator or the study doctor will inform you of your abnormal results. A copy of the results will be provided to you and you will be advised to discuss the results with your local GP. If further care is provided, the project coordinator can arrange an appointment with any member of our health care team, including endocrinologists and diabetes educators.

**9 What will happen to my test samples?**

On the five assessment days, blood samples will be collected on five occasions via a cannula (at baseline, 30 mins, 60 mins, 90 mins and 120 mins). The blood collected will be analysed for blood glucose, insulin, hs-CRP, HbA1c,fasting lipids (total cholesterol, triglycerides, HDLC), markers of chronic inflammation (e.g. TNFα, IL-6), neurogenesis (brain-derived neurotrophic factor). Approximately 100ml (equivalent to 6 tablespoons) will be collected over the course of each assessment day.

At each blood collection, some of the blood that is taken will be in excess to what is required for the tests described above. This spare sample may be needed to re-run the tests described above to ensure accurate results. We would also like your permission to store these extra samples for future research into diabetes, heart disease and cognitive function. These samples will be stored in identifiable format (e.g. using a participant ID number, with no names or contact details) indefinitely at -80°C in a locked freezer at the Baker Heart and Diabetes Institute and the Sunshine Coast Health Institute. Only study investigators will have access to your samples. By signing the Consent Form for tissue sample storage and use, you consent to the analysis of markers relevant to diabetes, heart disease and cognitive function in your blood samples. The storage of any excess sample is voluntary. We are also asking permission to use blood from your first visit to analyse your sample for potential genetic risk of dementia (APOE-4 status).

Once the study has been completed you will be provided with a summary of both your individual results and group data by mail. The non-identifiable group data will also be published in a peer reviewed scientific journal.

**10 What if new information arises during this research project?**

Sometimes during the course of a research project, new information becomes available about the treatment that is being studied. If this happens, your study doctor will tell you about it and discuss with you whether you want to continue in the research project. If you decide to withdraw, your study doctor will make arrangements for your regular health care to continue. If you decide to continue in the research project you will be asked to sign an updated consent form.

Also, on receiving new information, your study doctor might consider it to be in your best interests to withdraw you from the research project. If this happens, he/ she will explain the reasons and arrange for your regular health care to continue.

**11 Can I have other treatments during this research project?**

It is important to tell your study doctor and the study staff about any treatments or medications you may be taking, including over-the-counter medications, vitamins or herbal remedies, acupuncture or other alternative treatments. You should also tell your study doctor about any changes to these during your participation in the research project. Your study doctor should also explain to you which treatments or medications need to be stopped for the time you are involved in the research project.

**12 What if I withdraw from this research project?**

If you decide to withdraw from the project, please notify a member of the research team before you withdraw. This notice will allow that person or the research supervisor to discuss any health risks or special requirements linked to withdrawing.

If you do withdraw your consent during the research project, the study doctor and relevant study staff will not collect additional personal information from you, although personal information already collected will be retained to ensure that the results of the research project can be measured properly and to comply with law. You should be aware that data collected by the time you withdraw will form part of the research project results. If you do not want them to do this, you must tell them before you join the research project.

**13 Could this research project be stopped unexpectedly?**

This research project may be stopped unexpectedly for a variety of reasons including unacceptable side effects, but this is very unlikely.

**14 What happens when the research project ends?**

At the completion of the study, you will be provided with an individual report of your results. If requested, you can also receive a report of the main findings of the study and copies of any subsequent publications. Any incidental findings related to your participation will not be returned to you.

**Part 2 How is the research project being conducted?**

**15 What will happen to information about me?**

By signing the consent form you provide your consent to the relevant research staff collecting and using personal information about you for the research project. Any information obtained in connection with this research study that can identify you will remain confidential and will only be used for the purpose of this research study. It will only be disclosed with your permission, or in compliance with the journal requirements and the law.

**Where will my data be stored?**

This research study involves the establishment of a databank. When you sign the attached consent form, you are consenting your information being used for this specific study.

Your data will be stored electronically using four secure platforms:

**1)** **Research Electronic Data Capture (‘REDCap’):** All participant data will be stored using a Baker-approved data management tool called *Research Electronic Data Capture* (‘REDCap’). REDCap is a secure web application for building and managing online surveys and databases. The database will only be accessed by the researchers involved in the study. The data stored in REDCap is in identifiable format. However, only the project coordinators and support staff assisting in data collection will have access to your personal details. All other investigators on the study will be restricted to data that is re-identifiable (i.e. they will not have access to your name, contact details, etc.).

**2)** **LabArchives:** All pathology records such as frozen blood tubes stored in the -80°C freezer will be recorded in a secure cloud based e-notebook called LabArchives. In accordance with Australian privacy legislation, no personal identifying information will be entered into LabArchives, e.g. the data will be stored in a de-identifiable format. Accounts will be password protected and data will only be accessible by study investigators.

**3) Research Data Manager:** As many of the researchers involved in this study are from different Universities/Institutes, a shared and secure platform, called ‘Research Data Manager’ will be used to store the study materials (e.g. ethics application, study forms, meetings notes) and study data results. In accordance with Australian privacy legislation, no personal identifying information will be entered into the Research Data Manager and only named chief investigators and the project team approved by ethics will have access to this platform. All data is kept within Australia and the platform has relevant security and back-up requirements.

**4) Fitabase:** Fitabase is a comprehensive data management platform designed to support research projects using wearable and internet-connected devices. If you are in the ‘intervention group’, Fitabase will be used to collect all physical activity data collected by the Fitbit watch that you would be wearing whilst participating in the study. In accordance with Australian privacy legislation, no personal identifying information will be entered into Fitabase, e.g. the data will be stored in a de-identifiable format by participant ID only. The Fitabase account will be password protected and data will only be accessible by study investigators. At the completion of the study, Fitabase will not be used to collect any of your activity.

**5) Propelo:** propelo is a web-based platform used to send and receive tailored text messages. If you are in the ‘intervention group’, your data will be transferred to this platform to enable tailored messages to be sent. Data transferred to propelo will include first name only, mobile phone number and a range of variables about your text messaging preferences and behavioural goals. The propelo platform will be password protected and data will only be accessible by study investigators. At the completion of the study, propelo will no longer access your data**.**

Hard copy data will be stored in a locked filing cabinet in the Study Coordinator’s office at the Physical Activity laboratory, Baker Heart & Diabetes Institute, 99 Commercial Rd, Melbourne. The hard copy data will be stored in a re-identifiable (coded) format (with the exception of your consent form) for 15 years as per Alfred Hospital Study policy. Hard copy confidential records or materials of a personal nature will be destroyed by way of maintaining de-identification. This can be achieved by utilising the secure service provided to the organisation.

**Reports and publications**

It is anticipated that the results of this research study will be published and/or presented in a variety of forums, which may include publication in scientific journals, presentations at scientific conferences and clinical trial registries such as the Australia New Zealand Clinical Trial Registry (https://www.anzctr.org.au). Identifying information will not appear in any data reports or publications. Only group data will be published and presented. As a requirement for some scientific journals to ensure integrity of the study’s findings, the non-identifiable data underlying the findings described in the publication may need to be submitted to the journal for review. If this is the case, the data file provided will not contain information that can identify you (e.g. no names, contact details or addresses will be provided). In addition, some of your survey responses may be shared with the registered owners who have created the survey items. This is part of a user agreement license to use validated and reliable survey instruments. Similar to above, only non-identifiable data (such as your participant ID) and the relevant survey responses will be shared with the survey owners. Your identity as a participant in this study will remain confidential.

**Your rights**

In accordance with relevant Australian and Victorian privacy and other relevant laws, you have the right to request access to your information collected and stored by the research team. You also have the right to request that any information with which you disagree be corrected. Please contact the study team member named at the end of this document if you would like to access your information.

**Audits**

In accordance with the NHMRC National Statement, the Research Ethics Committee is required to conduct audits of research projects from time to time. It may therefore be possible that the Research Ethics Committee which has approved this research will seek to view a copy of your signed consent form, or to contact you, to ensure that the research is being conducted according to the ethical standards required by the National Statement.

**16 What if I get injured in this research?**

If you suffer any injuries or complications as a result of this research project, you should contact the study team as soon as possible and you will be assisted with arranging appropriate medical treatment. If you are eligible for Medicare, you can receive any medical treatment required to treat the injury or complication, free of charge, as a public patient in any Australian public hospital. If you ineligible for Medicare, you may be able to receive medical treatment through your private health insurer. You should contact your insurer before enrolling in this study to ensure any medical treatment you require in connection with participation falls within your policy’s terms and conditions.

**17 Who is organising and funding the research?**

This research study is being conducted and funded through two National Health and Medical Research Council (NHMRC) Project Grants and one Diabetes Australia Research Foundation grant. The project is led by Professor David Dunstan, Head of the Physical Activity Laboratory at Baker Heart and Diabetes Institute. Other grants are led by Dr Paul Gardiner of the Centre for Health Services Research at The University of Queensland and Associate Professor Genevieve Healy of the School of Public Health at The University of Queensland.

You will not benefit financially from your involvement in this research study even if, for example, your samples (or knowledge acquired from analysis of your samples) prove to be of commercial value to the Baker Heart and Diabetes Institute. In addition, if knowledge acquired through this research leads to discoveries that are of commercial value to the study team or their institutions, there will be no financial benefit to you or your family from these discoveries.

The Baker Heart and Diabetes Institute, The University of Queensland and University of the Sunshine Coast will receive payment for the direct research costs for this study from the NHMRC. No member of the research team will receive a personal financial benefit from your involvement in this research study (other than their ordinary wages).

**18 Who has reviewed the research project?**

All research in Australia involving humans is reviewed by an independent group of people called a Human Research Ethics Committee (HREC). The ethical aspects of this research project have been approved by the HREC of The Alfred Hospital, Melbourne.

This project will be carried out according to the *National Statement on Ethical Conduct in Human Research (2007)*. This statement has been developed to protect the interests of people who agree to participate in human research studies.

**19 Further information and who to contact**

**Study contact personal**

The person you may need to contact will depend on the nature of your query.

If you want any further information concerning this project or if you have any medical problems which may be related to your involvement in the project (for example, any side effects), you can contact the following people:

*Project Coordinator*

*Project support staff*

*Lead Investigators*

**Complaints**

**Consent Form - *Adult providing own consent***

| **Title** | Office-based Program To Improve Metabolic control In Sedentary Employees with type 2 diabetes |
| --- | --- |
| **Short Title** | The ‘OPTIMISE Your Health’ Study |
| **Protocol Number** | V10.0.0 |
| **Project Sponsor** | Baker Heart and Diabetes Institute |
| **Principal Investigator** | Prof David Dunstan |
| **Associate Investigators**  **(Australia)** | Prof Neville Owen, Prof Elizabeth Eakin, Prof Stuart Biddle, A/Prof Genevieve Healy, Prof Robin Daly, Prof Daniel Green, Prof Marjory Moodie, Dr Elisabeth Winkler, A/Prof Neale Cohen, Ms Ruth Grigg, Ms Kym Rickards, Ms Frances Taylor, Miss Melanie Townsend, Mr Christian Brakenridge, Dr Francis Dzakpasu, Dr Paul Gardiner, Dr Mia Schaumberg, Dr Brianna Fjeldsoe, Prof Len Gray, A/Prof Tracy Comans, Dr Ana Goode, Ms Ashleigh Homer. |
| **Associate Investigators (International)** | A/Prof Matthew Buman (USA) |
| **Location** | Physical Activity Laboratory, Baker Heart and Diabetes Institute, Melbourne, VIC. |

**Declaration by Participant**

I have read the Participant Information Sheet.

I understand the purposes, procedures and risks of the research described in the project.

I have had an opportunity to ask questions and I am satisfied with the answers I have received.

I freely agree to participate in this research project as described and understand that I am free to withdraw at any time during the study without affecting my future health care.

I understand that I will be given a signed copy of this document to keep.

I give permission for my doctors, other health professionals, hospitals or laboratories outside this hospital to release information to Baker concerning my condition and treatment for the purposes of this project. I understand that such information will remain confidential.

I understand that the researchers may contact me in the near future for any follow-up research relevant to my condition.

|  | | | | | | |
| --- | --- | --- | --- | --- | --- | --- |
|  | Name of Participant | |  |  |  |  |
|  | Signature |  | | Date |  |  |
|  | | | | | | |

**Declaration by Study Doctor/Senior Researcher^†^**

I have given a verbal explanation of the research project, its procedures and risks and I believe that the participant has understood that explanation.

|  | | | | | | |
| --- | --- | --- | --- | --- | --- | --- |
|  | Name of Study Doctor/  Senior Researcher^†^ (please print) | |  | | |  |
|  | | | | | |  |
|  | Signature |  | | Date |  |  |
|  | | | | | | |

^†^ A senior member of the research team must provide the explanation of, and information concerning, the research project.

Note: All parties signing the consent section must date their own signature.

**Consent Form For The Storage And Use Of**

**Additional Blood Samples**

| **Title** | Office-based Program To Improve Metabolic control In Sedentary Employees with type 2 diabetes |
| --- | --- |
| **Short Title** | The ‘OPTIMISE Your Health’ Study |
| **Protocol Number** | V10.0.0 |
| **Project Sponsor** | Baker Heart and Diabetes Institute |
| **Principal Investigator** | Prof David Dunstan |
| **Associate Investigators**  **(Australia)** | Prof Neville Owen, Prof Elizabeth Eakin, Prof Stuart Biddle, A/Prof Genevieve Healy, Prof Robin Daly, Prof Daniel Green, Prof Marjory Moodie, Dr Elisabeth Winkler, A/Prof Neale Cohen, Ms Ruth Grigg, Ms Kym Rickards, Ms Frances Taylor, Miss Melanie Townsend, Mr Christian Brakenridge, Dr Francis Dzakpasu, Dr Paul Gardiner, Dr Mia Schaumberg, Dr Brianna Fjeldsoe, Prof Len Gray, A/Prof Tracy Comans, Dr Ana Goode, Ms Ashleigh Homer. |
| **Associate Investigators (International)** | A/Prof Matthew Buman (USA) |
| **Location** | Physical Activity Laboratory, Baker Heart and Diabetes Institute, Melbourne, VIC. |

**Declaration by Participant**

I have read Section 9 of this Participant Information Form and am aware that extra blood is being collected for verification of pathology tests if required.

In the case that verification is not required, we ask for your consent to store and use these samples for future research into cardiovascular disease and diabetes (i.e. for future research to be approved by the Alfred Human Ethics Committee)

Note: Storage of extra samples is voluntary and will not affect your participation in the study. If you do not give consent, your samples will be destroyed at the conclusion of the study.

| Do you give consent for researchers to store and use these samples for future research into CVD and/or diabetes and/or cognitive function? | |
| --- | --- |
|  | *Please write “Yes” or “No”* |
|  | |
|  | |

|  | | | | | | |
| --- | --- | --- | --- | --- | --- | --- |
|  | Name of Participant (please print) | |  |  |  |  |
|  | | | | | | |
|  | Signature |  | | Date |  |  |
|  | | | | | | |

| Do you give consent for researchers to collect and analyse your blood for genetic markers related to Type 2 Diabetes and/or cognitive health? | |
| --- | --- |
|  | *Please write “Yes” or “No”* |
|  | |
|  | |

|  | | | | | | |
| --- | --- | --- | --- | --- | --- | --- |
|  | Name of Participant (please print) | |  |  |  |  |
|  | | | | | | |
|  | Signature |  | | Date |  |  |
|  | | | | | | |

**Declaration by Study Doctor/Senior Researcher^†^**

I have given a verbal explanation of the research project, its procedures and risks and I believe that the participant has understood that explanation.

|  | | | | | | |
| --- | --- | --- | --- | --- | --- | --- |
|  | Name of Study Doctor/  Senior Researcher^†^ (please print) | |  | | |  |
|  | | | | | |  |
|  | Signature |  | | Date |  |  |
|  | | | | | | |

^†^ A senior member of the research team must provide the explanation of, and information concerning, the research project.

Note: All parties signing the consent section must date their own signature.

**Consent Form For Testing of Genetic Material**

| **Title** | Office-based Program To Improve Metabolic control In Sedentary Employees with type 2 diabetes |
| --- | --- |
| **Short Title** | The ‘OPTIMISE Your Health’ Study |
| **Protocol Number** | V10.0 |
| **Project Sponsor** | Baker Heart and Diabetes Institute |
| **Principal Investigator** | Prof David Dunstan |
| **Associate Investigators**  **(Australia)** | Prof Neville Owen, Prof Elizabeth Eakin, Prof Stuart Biddle, A/Prof Genevieve Healy, Prof Robin Daly, Prof Daniel Green, Prof Marjory Moodie, Dr Elisabeth Winkler, A/Prof Neale Cohen, Ms Ruth Grigg, Ms Kym Rickards, Ms Frances Taylor, Miss Melanie Townsend, Mr Christian Brakenridge, Dr Francis Dzakpasu, Dr Paul Gardiner, Dr Mia Schaumberg, Dr Brianna Fjeldsoe, Prof Len Gray, A/Prof Tracy Comans, Dr Ana Goode. |
| **Associate Investigators (International)** | A/Prof Matthew Buman (USA) |
| **Location** | Physical Activity Laboratory, Baker Heart and Diabetes Institute, Melbourne, VIC. |

**Declaration by Participant**

I have read Section 9 of this Participant Information Form and am aware blood from the first visit will be used to test for potential genetic markers of dementia, i.e. APOE.

Note: This analysis is voluntary and will not affect your participation in the study. If you do not give consent, your samples will not be analysed and be destroyed at the conclusion of the study.

APOE-4 is a commonly assessed genetic risk marker in studies investigating cognitive decline. The presence of the APOE-4 gene does not indicate the presence of disease therefore you will not be provided with the results of this test.

| Do you give consent for researchers to test your blood for a potential marker of dementia, i.e. APOE? | |
| --- | --- |
|  | *Please write “Yes” or “No”* |
|  | |
|  | |

|  | | | | | | |
| --- | --- | --- | --- | --- | --- | --- |
|  | Name of Participant (please print) | |  |  |  |  |
|  | | | | | | |
|  | Signature |  | | Date |  |  |
|  | | | | | | |

| Do you give consent for researchers to collect and analyse your blood for genetic markers related to Type 2 Diabetes and/or cognitive health? | |
| --- | --- |
|  | *Please write “Yes” or “No”* |
|  | |
|  | |

|  | | | | | | |
| --- | --- | --- | --- | --- | --- | --- |
|  | Name of Participant (please print) | |  |  |  |  |
|  | | | | | | |
|  | Signature |  | | Date |  |  |
|  | | | | | | |

**Declaration by Study Doctor/Senior Researcher^†^**

I have given a verbal explanation of the research project, its procedures and risks and I believe that the participant has understood that explanation.

|  | | | | | | |
| --- | --- | --- | --- | --- | --- | --- |
|  | Name of Study Doctor/  Senior Researcher^†^ (please print) | |  | | |  |
|  | | | | | |  |
|  | Signature |  | | Date |  |  |
|  | | | | | | |

^†^ A senior member of the research team must provide the explanation of, and information concerning, the research project.

Note: All parties signing the consent section must date their own signature.

**Form for Withdrawal of Participation**

| **Title** | Office-based Program To Improve Metabolic controlIn Sedentary Employees with type 2 diabetes |
| --- | --- |
| **Short Title** | The ‘OPTIMISE Your Health’ Study |
| **Protocol Number** | V10.0 |
| **Project Sponsor** | Baker Heart and Diabetes Institute |
| **Principal Investigator** | Prof David Dunstan |
| **Associate Investigators**  **(Australia)** | Prof Neville Owen, Prof Elizabeth Eakin, Prof Stuart Biddle, A/Prof Genevieve Healy, Prof Robin Daly, Prof Daniel Green, Prof Marjory Moodie, Dr Elisabeth Winkler, A/Prof Neale Cohen, Ms Ruth Grigg, Ms Kym Rickards, Ms Frances Taylor, Miss Melanie Townsend, Mr Christian Brakenridge, Dr Francis Dzakpasu, Dr Paul Gardiner, Dr Mia Schaumberg, Dr Brianna Fjeldsoe, Prof Len Gray, A/Prof Tracy Comans, Dr Ana Goode, Ms Ashleigh Homer |
| **Associate Investigators (International)** | A/Prof Matthew Buman (USA) |
| **Location** | Physical Activity Laboratory, Baker Heart and Diabetes Institute, Melbourne, VIC. |

**Declaration by Participant**

I wish to withdraw from participation in the above research project and understand that such withdrawal will not affect my routine treatment, my relationship with those treating me or my relationship with Baker Heart and Diabetes Institute.

|  | | | | | | |
| --- | --- | --- | --- | --- | --- | --- |
|  | Name of Participant (please print) | |  |  |  |  |
|  | | | | | | |
|  | Signature |  | | Date |  |  |
|  | | | | | | |

*In the event that the participant’s decision to withdraw is communicated verbally, the Study Doctor/Senior Researcher will need to provide a description of the circumstances below.*

|  |
| --- |

**Declaration by Study Doctor/Senior Researcher^†^**

I have given a verbal explanation of the implications of withdrawal from the research project and I believe that the participant has understood that explanation.

|  | | | | | | |
| --- | --- | --- | --- | --- | --- | --- |
|  | Name of Study Doctor/  Senior Researcher^†^ (please print) | |  | | |  |
|  | | | | | |  |
|  | Signature |  | | Date |  |  |
|  | | | | | | |

^†^ A senior member of the research team must provide the explanation of and information concerning withdrawal from the research project.

Note: All parties signing the consent section must date their own signature.
