# Supplementary material for: Sitting less and moving more for improved metabolic and brain health in type 2 diabetes: ‘OPTIMISE your health’ trial protocol
Source: BMC Public Health. 2022 May 10;22:929. doi: 10.1186/s12889-022-13123-x (PMC9086419; doi:10.1186/s12889-022-13123-x)
Supplement: Supplementary file 3 — Additional file 3. Inclusion and exclusion criteria. [file 12889_2022_13123_MOESM3_ESM.docx]

**To be included in the study, participants must be:**

- Aged between 35-65 years;
- Have a body mass index (BMI) between 25-45 kg/m^2^;
- Be medically diagnosed with T2D for at least three months
- T2D to be treated by diet alone or with oral hypoglycaemic agents or GLP1 agonists and be on stable treatment regimen for > 3 months
- Stable body weight (+/-5 kg) for > 3 months prior to beginning the intervention
- Working at least 0.8 full-time equivalent in a desk-based occupation for > 6 months with the same employer and able to obtain employer permission to install the provided sit–stand workstation on their work surface.

**Participants are unable to participate in the study if they are:**

- Using insulin to treat T2D
- HbA1c <6.5% (indicative of good control) or >10% (reflects uncontrolled);
- Pregnant
- Women of childbearing potential not currently using adequate contraception
- Non-Smartphone user
- Currently using a height adjustable workstation at their workplace
- Regularly engaged in moderate-vigorous intensity exercise ≥ 30 min/day for >3 months
- Regularly engaged in > 30 minutes/week of structured strength/resistance training (i.e. involving machine or free weights) for > 3 months
- Regularly sits for < 7 hours per day for > 3 months
- Major illness/physical problems (acute or chronic) that may limit participation in the intervention.
- Significant cardiovascular disease (unstable angina, cardiac failure) or recent myocardial infarction, coronary artery bypass graft, ischaemic or haemorrhagic stroke in the previous 3 months
- Cancer diagnosis in the previous 12 months
- A medically diagnosed and untreated severe sleeping disorder
- Planning to move from the current employer in the next 6 months
- Intending to have an extended vacation (> 4 weeks) in the next 6 months
- Unable to communicate in English
- Unable to provide written informed consent
