# Supplementary material for: Sitting less and moving more for improved metabolic and brain health in type 2 diabetes: ‘OPTIMISE your health’ trial protocol
Source: BMC Public Health. 2022 May 10;22:929. doi: 10.1186/s12889-022-13123-x (PMC9086419; doi:10.1186/s12889-022-13123-x)
Supplement: Supplementary file 4 — Additional file 4. Intervention handbook. [file 12889_2022_13123_MOESM4_ESM.pdf]

Contents

Introduction ____ 2
Goal #1—Sit less! ____ 3
Sit-stand workstations ____ 4
Desk ergonomics ____ 5
Goal #2—Move More! ____ 6
Fitbit—App Instructions ____ 7
Fitbit—Watch Instructions ____ 9
Simple Resistance Activities ____ 10
Logging your 'SRA' breaks ____ 12
At work—Strategies to Sit Less ____ 13
At work—Strategies to Move More ____ 14
Outside of work—Strategies to Sit Less ____ 15
Outside of work—Strategies to Move More ____ 16
Face to Face Health Coaching Session #1 ____ 17
Telephone Health Coaching Phone Call #1 ____ 18
Telephone Health Coaching Phone Call #2 ____ 19
Telephone Health Coaching Phone Call #3 ____ 20
Telephone Health Coaching Phone Call #4 ____ 21
Telephone Health Coaching Phone Call #5 ____ 22
Face to Face Health Coaching Session #2 ____ 23
Telephone Health Coaching Phone Call #6 ____ 24
Telephone Health Coaching Phone Call #7 ____ 25
Telephone Health Coaching Phone Call #8 ____ 26
Frequently Asked Questions ____ 27
Study Timeline ____ 28

Introduction

INTRODUCTION

It is well known that being physically active is important for maintaining good health. However, new evidence shows that being sedentary (sitting for prolonged periods) is associated with elevated blood glucose and insulin – two important risk factors for cardiovascular disease. Interestingly, excessive daily sitting (of which 60% is accrued at the workplace) has been shown to increase the risk of developing cardiovascular disease (the most common complication observed in adults with type 2 diabetes). This highlights a need to explore alternative, practical, and low risk approaches to reduce sitting time across all spheres of daily living (i.e. work and home).

Recent evidence suggests that **reducing and breaking up sitting time** may be a suitable self-care behaviour to improve glucose control in adults with type 2 diabetes. Our laboratory has recently shown that regularly interrupting sitting time can improve blood glucose levels, blood pressure and improve measures of blood vessel health.

ABOUT THE 'OPTIMISE YOUR HEALTH' STUDY

The 'OPTIMISE Your Health' study aims to test whether 'sitting less' and 'moving more' can improve blood glucose control as well as risk markers for cardiovascular disease (blood pressure and blood vessel health). Throughout the study, the health coaches will work closely with you to help you to sit less and move more throughout the working day and outside of work.

This booklet contains information on how we will help you to 'sit less' (e.g. by using the sit-stand workstation) and to 'move more' (e.g. by using your Fitbit watch). Throughout the study, we will be contacting you regularly to help you transition to a less sedentary and more active lifestyle.

Goal 1 - Sit Less!

SIT LESS

The average Australian adult sits for over 9 hours a day, with office based workers most at risk of sitting for long periods throughout the day. Our ultimate goal for the study is to work closely with you to try to reduce the time spent sitting, and to break up your long bouts of sitting time. To do this, we ask all participants to try and reach a 50/50 split across the whole day, meaning 50% sitting and 50% not sitting (e.g. standing or moving).

TO HELP YOU SIT LESS

To help you reach the 50/50 split, the 'OPTIMISE Your Health' study will provide you with a sit-stand workstation that will allow you to stand up at your desk whilst you continue to work. We will also work closely with you to establish your own individual goals to SIT LESS and incorporate other strategies to help you achieve these goals.

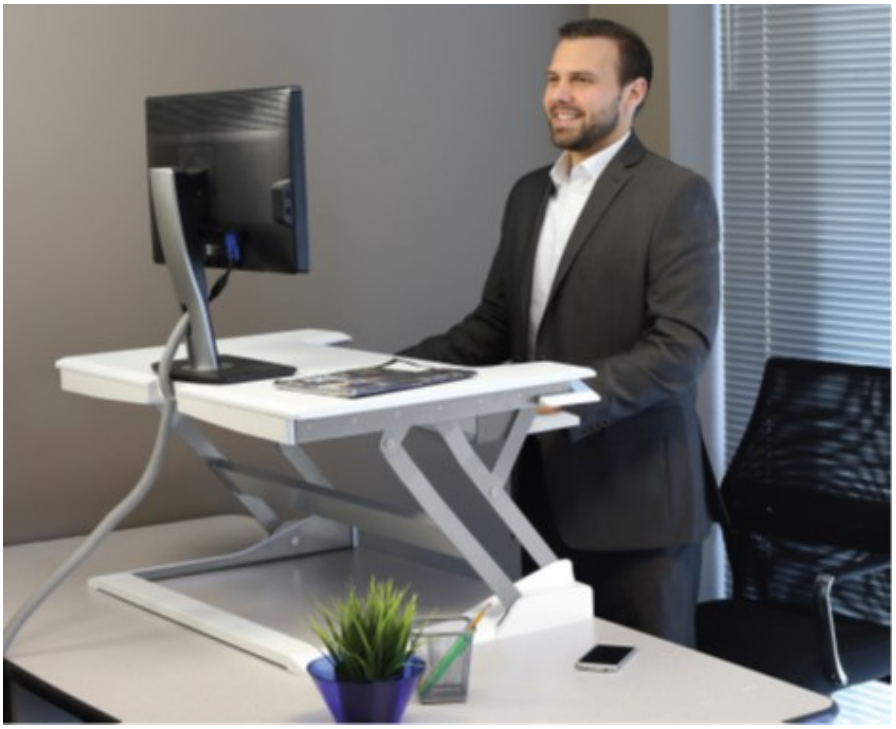

Baker HEART & DIABETES INSTITUTE

OPTIMISE
OPTIMISE YOUR HEALTH Study

Sit-Stand Workstation

GETTING THE MOST OUT OF YOUR SIT-STAND WORKSTATION

- The sit-stand workstation will help you to sit less by replacing some of your sitting with standing, whilst continuing to work.

- The regular health coaching sessions will help you determine goals necessary to reduce your sitting time. These goals will be incremental and progress over time as you get used to the standing desk.

- An initial SIT LESS goal could be to replace the first 5 minutes of the hour with standing or moving.

- Prolonged standing may create muscular discomfort in your feet, legs, or back. Remember to always listen to your body and make sure you make an effort to always change between sitting, standing and moving.

- If you can, set up your workstation such that you are not directly overlooking your colleagues. This is important so as not to create too much interference in the office space.

- In order to support more standing at your work station, you can add an anti-fatigue standing mat. Ensure that the mat is lightweight and thin so as not to be a trip hazard.

- We encourage you to make other changes to your office environment and office systems that encourage sitting less and moving more. For example locate printers and rubbish bins away from your immediate work area so you need to walk to them, or encourage use of stairs instead of lifts or standing during meetings.

Desk Ergonomics

TOP 10 TIPS FOR SETTING UP YOUR SIT-STAND WORKSTATION:

1) Position the top of your monitor screen at eye level. If using bifocals, lower the monitor further and turn screen upward
2) Tilt your monitor back 10° to 20° to keep the same focal length as your eyes scan from the top to bottom of screen. If using glasses, use a 30° to 40° angle
3) Position your monitor no closer than 50 cm from your eyes. A good guide is an arm's length distance. The larger the screen, the more distance you'll need. With bifocals, the distance should be 40 cm, matching the lenses' focal length
4) The top of your keyboard should be level with the height of your elbow
5) Tilt your keyboard back slightly so that your wrists remain flat
6) Use a wrist rest so your hands and wrists remain relaxed
7) Rest your eyes periodically by focusing on an object 6 + metres away
8) Stand and stretch your back and arms from time to time
9) Use an easily adjusted chair, display mount and keyboard tray. Change the position of your display and keyboard to accommodate reflexive changes in your posture
10) Arrange your workstation in a way that allows you to stand periodically while computing

Remember! Even if your workspace is set up properly you can still get muscle fatigue from maintaining the same posture for too long—adjust the position of your monitor, keyboard and chair as your posture changes.

Baker HEART & DIABETES INSTITUTE

OPTIMISE
OPTIMISE YOUR HEALTH Study

Goal 2 - Move More!

MOVE MORE

The second goal of the study is to MOVE MORE, by replacing some of your sitting time with moving time. This can be accomplished by breaking up your sitting by walking around your workplace and/or by performing 'simple resistance activities' at your desk. Our goal is to increase the number of steps you take each week, so that you are moving more by the end of the study.

As a guide, we suggest you increase the number of steps slowly and gradually, until you have reached your end goal. For example, you might like to increase your steps by 10% in the first week. This means that if you are currently getting 5,000 steps per day, you could aim to increase your steps to 5,500 steps per day.

INCREASING STEPS

As a general rule, it takes about 1 minute to achieve approximately 100 steps. Therefore, if your goal was to take an extra 1,000 steps each day, that would require an extra 10 minutes of walking. It doesn't take much for the steps to add up, so try take a 5-10 minute walk any time you can!

TO HELP YOU MOVE MORE

In order to help you MOVE MORE, you will be provided a Fitbit watch to use throughout the study. The Fitbit watch allows you to monitor your activity by seeing how many steps you take each day.

You can also set reminders to help you MOVE MORE throughout the day by trying to move at least 250 steps each hour.

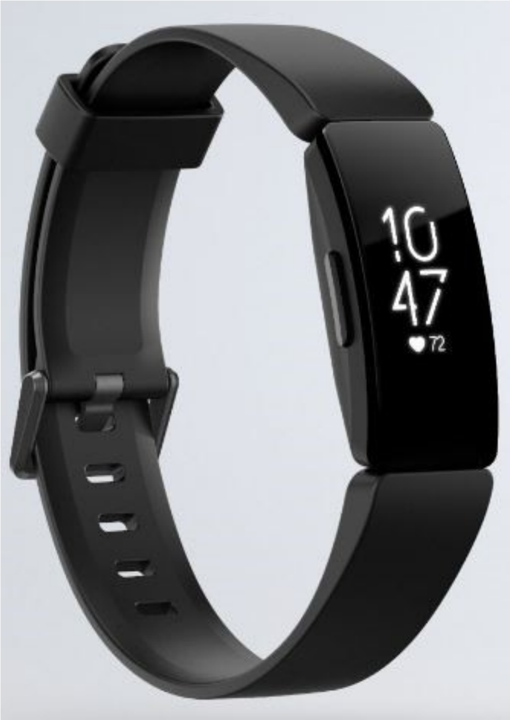

Fitbit—App Instructions

The Fitbit App has a many features. We recommend you have a look through the array of options!

The Fitbit home screen, better known as the **dashboard** displays a number of handy options that let you know how you are tracking with your activity and health.

These include:

Active Breaks/Reminders to Move: This is a key part of the OPTIMSE study. This section is covered further in the next few pages. Each red dot represents an hour of the day that you have broken up prolonged idle time with movement.

Steps: Your steps will be measured over the course of a day. Accomplishing your 'step goal' will complete the step wheel. You can tap the steps feature for a more detailed breakdown of the entire time you have worn the watch. To edit your 'step goal', you can do this by clicking on Account (top right corner) —> Activity —> and changing the 'steps'.

Floors, Distance, Calories, Active Minutes: These features will allow you to gain a better overview of how you have moved during the day. Active minutes (the lightning bolt) describes bouts of activity over 10 minutes conducted in the moderate intensity heart rate range.

Exercise: Depicts how many days you have engaged in some moderate to vigorous intensity exercise. Tap further to get additional breakdown information of your exercise time.

Heart Rate: Depicts your average resting heart rate in beats per minute. Tap on the icon to gain insight as to how your resting heart rate has changed over the last 30 days.

Weight, Water, Eating, Sleep: Feel free to play around with these features.

Fitbit—App Instructions

Active breaks / Reminders to move

Finding time to sit less and move more can be challenging. It is also important to distinguish this advice from traditional advice advocating activity before and after work rather than finding time throughout the day to get in regular bouts of activity. Throughout the study, you will work closely with your health coach to create goals as to how much you want to stand per hour, or how many minutes of extra walking you would like to strive for. The Fitbit will also help to prompt you every hour to break up your idle time.

You can achieve a ‘Fitbit break’ by taking 250 steps each hour. If you haven’t taken 250 steps within the hour, you will receive a notification at the 50 minute mark on your Fitbit watch to remind you to move. Try to engage in as many ’Fitbit breaks’ as you can.

Looking at the ‘Hourly Activity’ Figure, when a ‘Fitbit break’ is achieved, the grey coloured dot turns red. In this example, this person achieved 10 ‘Fitbit’ breaks across the day.

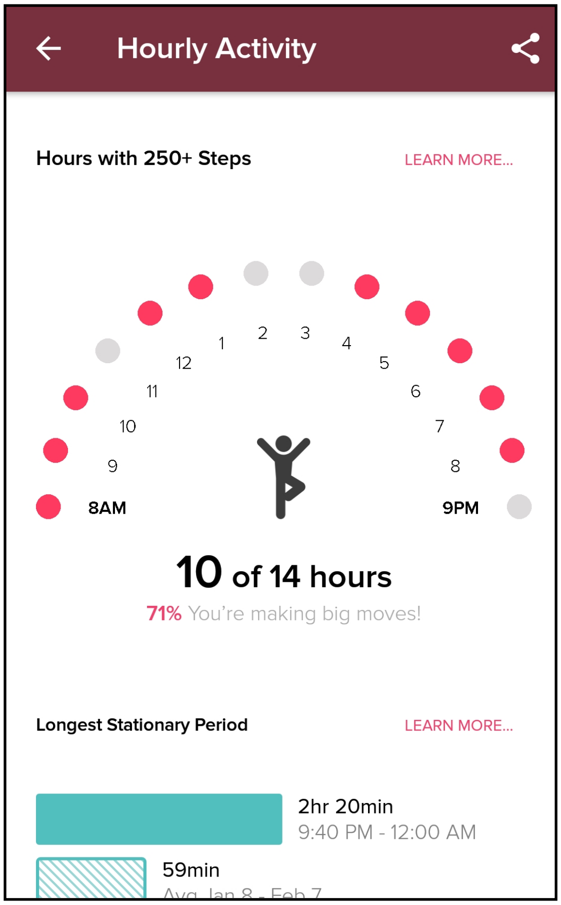

Tips for Active Breaks

- You can reach the ‘Hourly Activity’ page on your Fitbit app.
- Remember that some moving is always better than no moving.
- Remember to listen to your body and look more towards sustained improvement with small increments rather than big changes right from the start.
- Unfortunately, the Fitbit won’t recognise the ’Simple Resistance Activity’ breaks as a ’Fitbit break’. You will need to log this separately (see Page 13 for more details)

OPTIMISE
OPTIMISE YOUR HEALTH Study

Baker
HEART & DIABETES INSTITUTE

Fitbit — Watch Instructions

Day to day operations:

- Your watch screen comes to life when you: 1) actively turn your wrist to face you (screen wake); 2) when you tap the screen; and 3) if you pinch the sides of your watch. If the screen wake feature is disruptive (e.g. in your sleep) you can pinch and hold your Fitbit which will bring up settings to turn off screen wake.
- Swiping up on the Fitbit watch brings up your current day progress similar to the Fitbit app dashboard. This is a way to review your current heart rate, your steps accomplished per day, your active breaks etc.
- Swiping left on your watch will allow you to look through the activity options
- If you ever need to go back to the home screen, pinch the sides of the device. This will cause a vibration if done correctly.

How long does the battery last?

The Fitbit Inspire HR battery lasts up to 5 days. It is recommended that you charge it at least every 4 days either overnight whilst sleeping or when you are resting so as not to miss any steps!

How do I charge my Fitbit watch?

The Fitbit devices comes with a USB charger that plugs into a computer or a wall dock. To charge the Fitbit, attach the prongs on the magnetic charger to the back of the watch. If you have connected up the prongs correctly, you should get a picture of a battery recharging on the watch. Charging from 0% takes approximately 2—3 hours.

Do I sleep with my Fitbit watch?

Yes please! This will provide an overview of your sleep quality.

Is the Fitbit watch waterproof?

Yes, your Fitbit watch is water resistant to 50 metres. This means that you can swim with the device on, take a bath, shower.

Simple Resistance Activities

As well as taking 'walking breaks' we recommend completing some 'simple resistance activity' breaks. These activities are designed to be completed at your desk. Each exercise takes 20 seconds to complete, and should be completed 3 times to add up to a total of 3 minutes.

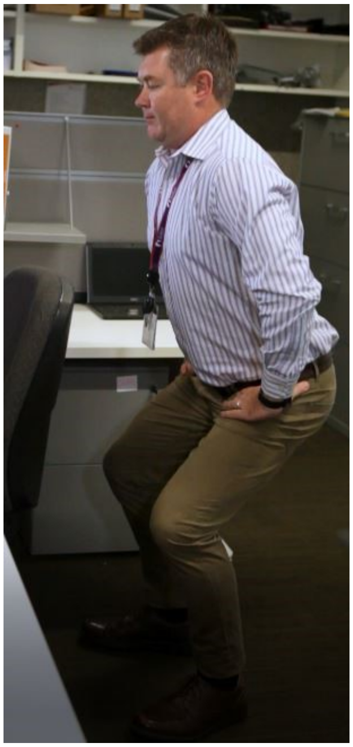

1. Squats

The first exercise is a squat. To correctly perform a squat, stand with your feet slightly wider than hip width apart, with your toes facing forward. Bending at the knees and ankles, drive your hips back as if you are moving into a sitting position, then return to a standing position, squeezing your buttocks to help you rise. Ensure your chest is up and your shoulders are back. Perform this exercise repeatedly for 20 seconds.

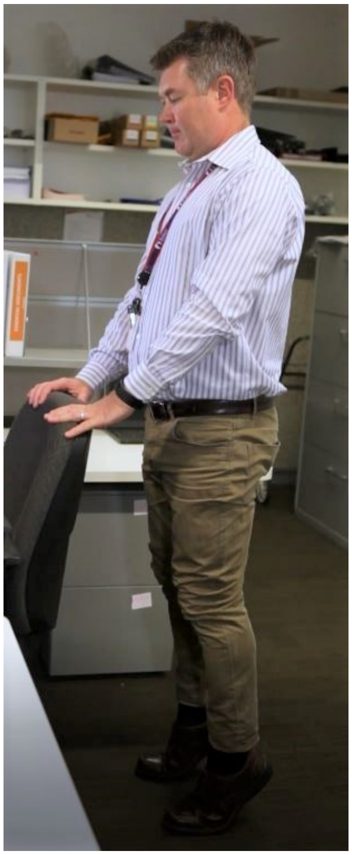

2. Standing calf raise

The second exercise is a calf raise. To perform a calf raise stand up straight, with your feet hip width apart. It's a good idea to perform this exercise holding onto your chair or desk for balance. Raise your heels up a few inches so you are up on your toes, then lower slowly. Perform this exercise repeatedly for 20 seconds before moving on to the next exercise.

Simple Resistance Activities

3. Kickbacks

The last of the three exercises is a kickback. To perform a kick back, stand up straight with your feet hip width apart. Without bending forward at the hips, engage your buttocks and extend your leg straight out behind you. It's a good idea to perform this exercise holding onto your chair or desk for balance.

Perform this exercise repeatedly for 20 seconds, alternating legs.

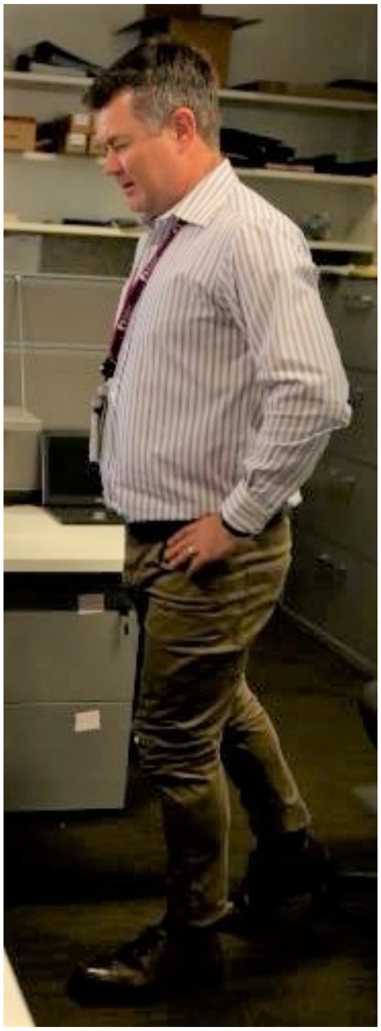

Logging your 'SRA' Breaks

In order to log a 'Simple Resistance Activity' (SRA) break in your Fitbit, perform the following steps:

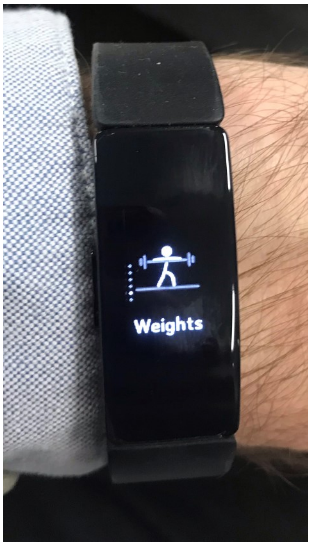

1. Swipe down once to get to the exercise menu.
2. Tap exercise
3. Swipe up until you reach "weights", click weights,
4. Tap start.
5. Once your 3 minutes of SRA is up— perform a pinching action on either side of the Fitbit which should cause the device to vibrate. Press it once again to be presented with a "finish" prompt.
6. Tap finish!

Once it is synchronised with your phone app—this should result in the SRA being logged as a weights bout as below:

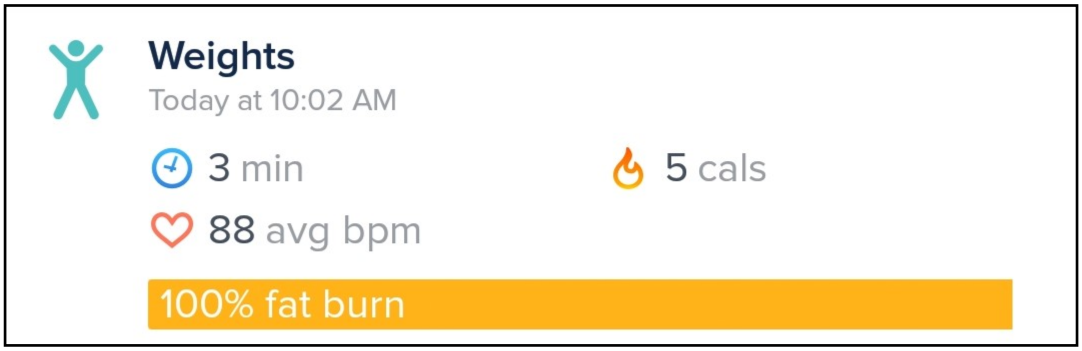

NOTES:

- Synchronisation will not occur immediately , you can start the process by using your smartphone application and pulling down on the screen to start a synchronisation.
- This process is not mandatory—it is a way to keep record of the SRAs that you have completed. However, any detail is certainly appreciated by the research team!

OPTIMISE
OPTIMISE YOUR HEALTH Study

Baker
HEART & DIABETES INSTITUTE

At Work— Strategies to 'Sit Less'

- ☐ 1) Use the sit-stand workstation in the 'standing' position at your desk
- ☐ 2) Leave your desk in the standing position when you go for breaks
- ☐ 3) Stand up at regular intervals
- ☐ 4) Stand up for each phone call
- ☐ 5) Stand up for checking or writing emails
- ☐ 6) Stand when feeling tired and/or uncomfortable
- ☐ 7) Stand up when drinking water
- ☐ 8) Stand up when you visit colleagues at their desks
- ☐ 9) Stand up after completing a work task
- ☐ 10) Stand up during meetings
- ☐ 11) Stand up at the back of the room during presentations
- ☐ 12) Set a timer on your phone to remind you to stand up
- ☐ 13) Your own: ____
  ____
- ☐ 14) Your own: ____
  ____

At Work— Strategies to 'Move More'

- ☐ 1) Set your Fitbit to remind you to move (at least 250 steps) every hour
- ☐ 2) iMails (walk over and talk) instead of eMails to colleagues
- ☐ 3) Remove bins/printers from each desk and use a central one
- ☐ 4) Drink more water so you have to go to the water cooler (and bathroom) more often
- ☐ 5) Using glasses to drink water and filling up the glass more regularly
- ☐ 6) Walk to a bathroom that is further away
- ☐ 7) Step outside for fresh air
- ☐ 8) Use the stairs instead of a lift
- ☐ 9) Have lunch away from your desk
- ☐ 10) Go for a short walk at lunch time (tip: bring in footwear and clothing you can comfortably walk in)
- ☐ 11) Find a walking buddy for your lunch time walks
- ☐ 12) Going for a short walk after a certain task (e.g. phone call)
- ☐ 13) Take the longer route around the office
- ☐ 14) Using a printer (picking up printing) further away from your desk
- ☐ 15) Organise walking meetings
- ☐ 16) Stand up and move around during meetings
- ☐ 17) Check your Fitbit steps prior to leaving work, to see how many more you need to meet your daily step goal.
- ☐ 18) Your own: ____
  ____
- ☐ 19) Your own: ____
  ____

Outside of Work— Strategies to Sit Less

- ☐ 1) Use your laptop on the kitchen worktop, or somewhere where you can stand
- ☐ 2) Stand up while on public transport, or waiting for the bus/train/tram.
- ☐ 3) Stand up while watching your children/grandchildren's sporting events.
- ☐ 4) While watching TV, do household chores such as folding clothes, washing dishes, or ironing during commercial breaks
- ☐ 5) Stand up and walk around while talking on the phone
- ☐ 6) When sitting down while reading a book, get up every few pages.
- ☐ 7) Stand up while reading the morning newspaper, mail, or email
- ☐ 8) Move around the house when checking text messages or email on your phone
- ☐ 9) Wash your car by hand instead of using a drive-through car wash
- ☐ 10) Break up sitting time with little jobs, instead of working straight for longer periods then sitting for longer periods
- ☐ 11) Stand during intervals at sporting events, theatres or concerts.
- ☐ 12) Set a timer on your phone with Siri or Ok Google to remind you to stand up and move
- ☐ 13) Your own: ____
  ____
- ☐ 14) Your own: ____
  ____

Outside of Work— Strategies to 'Move More'

☐ 1) Use an active way of commuting to and from work (walk or ride your bike)

☐ 2) Try to have reached your step goal prior to settling in for the evening

☐ 3) Join a social walking group, gym or activity class

☐ 4) Park your car further away from your work or other destination and walk a bit instead

☐ 5) Choose more active ways of commuting: cycle or walk all the way or use public transport so you have to cycle/walk to the next transport stop

☐ 6) Walk to visit neighbours instead of calling them

☐ 7) While watching TV, get off the couch and walk around the house during commercial breaks

☐ 8) Put the remote control next to the TV or on a cupboard so that it is away from where you are sitting while watching TV

☐ 9) Your own: ____
____

☐ 10) Your own: ____
____

Face to Face Health Coaching Session #1

Study timeline: Week '0'

Date: ____

Health coach: ____

Notes: ____

____

____

____

____

____

____

____

____

____

____

____

____

____

____

____

____

____

____

____

____

____

____

____

Baker HEART & DIABETES INSTITUTE

OPTIMISE
OPTIMISE YOUR HEALTH Study

Telephone Health Coaching Phone Call #1

Study timeline: Week '1'

Date: ____

Health coach: ____

Notes: ____

____

____

____

____

____

____

____

____

____

____

____

____

____

____

____

____

____

____

____

____

____

____

____

OPTIMISE

OPTIMISE YOUR HEALTH Study

Baker

HEART & DIABETES INSTITUTE

Telephone Health Coaching Phone Call #2

Study timeline: Week '2'

Date: ____

Health coach: ____

Notes: ____

____

____

____

____

____

____

____

____

____

____

____

____

____

____

____

____

____

____

____

____

____

____

____

____

Baker HEART & DIABETES INSTITUTE

OPTIMISE
OPTIMISE YOUR HEALTH Study

Telephone Health Coaching Phone Call #3

Study timeline: Week '3'

Date: ____

Health coach: ____

Notes: ____

____

____

____

____

____

____

____

____

____

____

____

____

____

____

____

____

____

____

____

____

____

____

____

Telephone Health Coaching Phone Call #4

Study timeline: Week '6'

Date: ____

Health coach: ____

Notes: ____

____

____

____

____

____

____

____

____

____

____

____

____

____

____

____

____

____

____

____

____

____

____

____

____

Telephone Health Coaching Phone Call #5

Study timeline: Week '9'

Date: ____

Health coach: ____

Notes: ____

____

____

____

____

____

____

____

____

____

____

____

____

____

____

____

____

____

____

____

____

____

____

____

Face to Face Health Coaching Session #2 (Baker)

Study timeline: Week '12'

Date: ____

Health coach: ____

Notes: ____

____

____

____

____

____

____

____

____

____

____

____

____

____

____

____

____

____

____

____

____

____

____

____

____

Telephone Health Coaching Phone Call #6

Study timeline: Week '15'

Date: ____

Health coach: ____

Notes: ____

____

____

____

____

____

____

____

____

____

____

____

____

____

____

____

____

____

____

____

____

____

____

____

Telephone Health Coaching Phone Call #7

Study timeline: Week '18'

Date: ____

Health coach: ____

Notes: ____

____

____

____

____

____

____

____

____

____

____

____

____

____

____

____

____

____

____

____

____

____

____

____

____

Telephone Health Coaching Phone Call #8

Study timeline: Week '21'

Date: ____

Health coach: ____

Notes: ____

____

____

____

____

____

____

____

____

____

____

____

____

____

____

____

____

____

____

____

____

____

____

____

Frequently Asked Questions

- How many steps do I need to take each day?
  - ⇒ Current physical activity guidelines in Australia advocate for 10,000 steps per day.

- What if I don’t reach my goals?
  - ⇒ Don’t worry! Continue to try to improve both sitting less and moving more, if you are not meeting your goals you can try a smaller goal and look to improve from there.

- I walk to and from work everyday, isn’t that enough?
  - ⇒ The adverse health effects of sitting unfortunately cannot be reconciled with physical activity at the bookends of each day. We need to move more regularly throughout the entire day to maximise the best health benefits.

- Does Fitbit log my time walking, running, cycling
  - ⇒ Fitbit does some automated logging, mainly with running and walking and it knows when you are idle. However, if you are engaging in a structured task such as swimming, riding a bike, or running on a treadmill it is recommended that you log this activity with the “Exercise” feature on the Fitbit.

- I am having difficulty syncing my data from my Fitbit to my phone?
  - ⇒ Fitbit has a number of troubleshooting options for this issue. Try first restarting your Bluetooth connection and ensuring your Fitbit watch is sufficiently charged. Failing this, you can un-pair the device from your App and re-pair it which should re-enable the synchronisation process.

Study Timeline

TIMEPOINT	ACTIVITY
Week 0	Sit-Stand Desk installation +<br>Face-to-face Health coaching #1
Week 1	Telephone call #1
Week 2	Telephone call #2
Week 3	Telephone call #3
Week 4	
Week 5	
Week 6	Telephone call #4
Week 7	
Week 8	
Week 9	Telephone call #5
Week 10<br>Week 11	Wear physical activity monitors (10 days)
Week 12	**3 MONTH VISIT @ BAKER**<br>Face-to-face health coaching #2
Week 13	
Week 14	
Week 15	Telephone call #6
Week 16	
Week 17	
Week 18	Telephone call #7
Week 19	
Week 20	
Week 21	Telephone call #8
Week 22<br>Week 23	Wear physical activity monitors (10 days)
Week 24	**6 MONTH VISIT @ BAKER**
**Week 25-52**	Text message health coaching<br>Telephone call #9

OPTIMISE
OPTIMISE YOUR HEALTH Study

Baker
HEART & DIABETES INSTITUTE
