# Supplementary material for: Sitting less and moving more for improved metabolic and brain health in type 2 diabetes: ‘OPTIMISE your health’ trial protocol
Source: BMC Public Health. 2022 May 10;22:929. doi: 10.1186/s12889-022-13123-x (PMC9086419; doi:10.1186/s12889-022-13123-x)

‘OPTIMISE Your Health’ Study

Activity Monitor Report

This report is for: Participant Name

Prepared on: 2/03/2022

This report was prepared by:
Baker Heart and Diabetes Institute

Dear Participant Name,

Thank you for taking part in the ‘**OPTIMISE Your Health’ study**. This is your personal feedback report from the thigh monitor you wore from Date1 to Date2. The monitor tells us when and how much you sit or lie down (referred to as sitting in this report) and how often you step (referred to as moving in this report). However, if something doesn’t seem right or is confusing, don’t worry, you can discuss that with your Optimise coach at your first meeting.

The report shows only your waking hours (not counting sleep or when you were not wearing the monitor). There is also information specifically about your work hours when this was provided in your diary.

Why is sitting less and moving more important ?

Too much sitting has now been linked to poor health and early death. In particular, sitting for long periods at a time is related to less healthy levels of many health markers, including body mass index, waist circumference, blood glucose levels, and triglycerides.

On the other hand, regularly breaking up sitting time has been shown to have health benefits. Standing and stepping (or ‘moving’) are simple ways to break up and reduce sitting time. When you meet your coach, you’ll be talking about how you can do this during work and at home.

Thank you again for taking part in the study. Please contact us at any time if you have any questions.

Best wishes,

SIGNATURE

Your sitting time overall and during work

The bar graph below shows your time during the day and during work. It is divided between sitting, standing and moving time.

The overall 'OPTIMISE Your Health' study goal is 50/50; meaning 50% of the time (or less) is spent sitting and the other 50% of the time (or more) is spent standing up or moving around.

During the day, your assessment indicates you spend on average, 9.0 hours per day sitting, which is 65% of your day. The rest of the time you are either standing (22%) or moving (13%). Another way to look at this is to break it down into 'per hour'. For example, a typical hour would involve about 39 minutes of sitting, 13 minutes of standing and 8 minutes of moving. According to your assessment, you are not meeting the 50/50 goal.

During work, based on your assessment, you typically spend about 80% of the time sitting, and the remaining time standing (14%) or moving (5%). This is a bit more sitting than you do in general. For you, a typical hour of work would involve about 48 minutes of sitting, 9 minutes of standing and 3 minutes of moving.

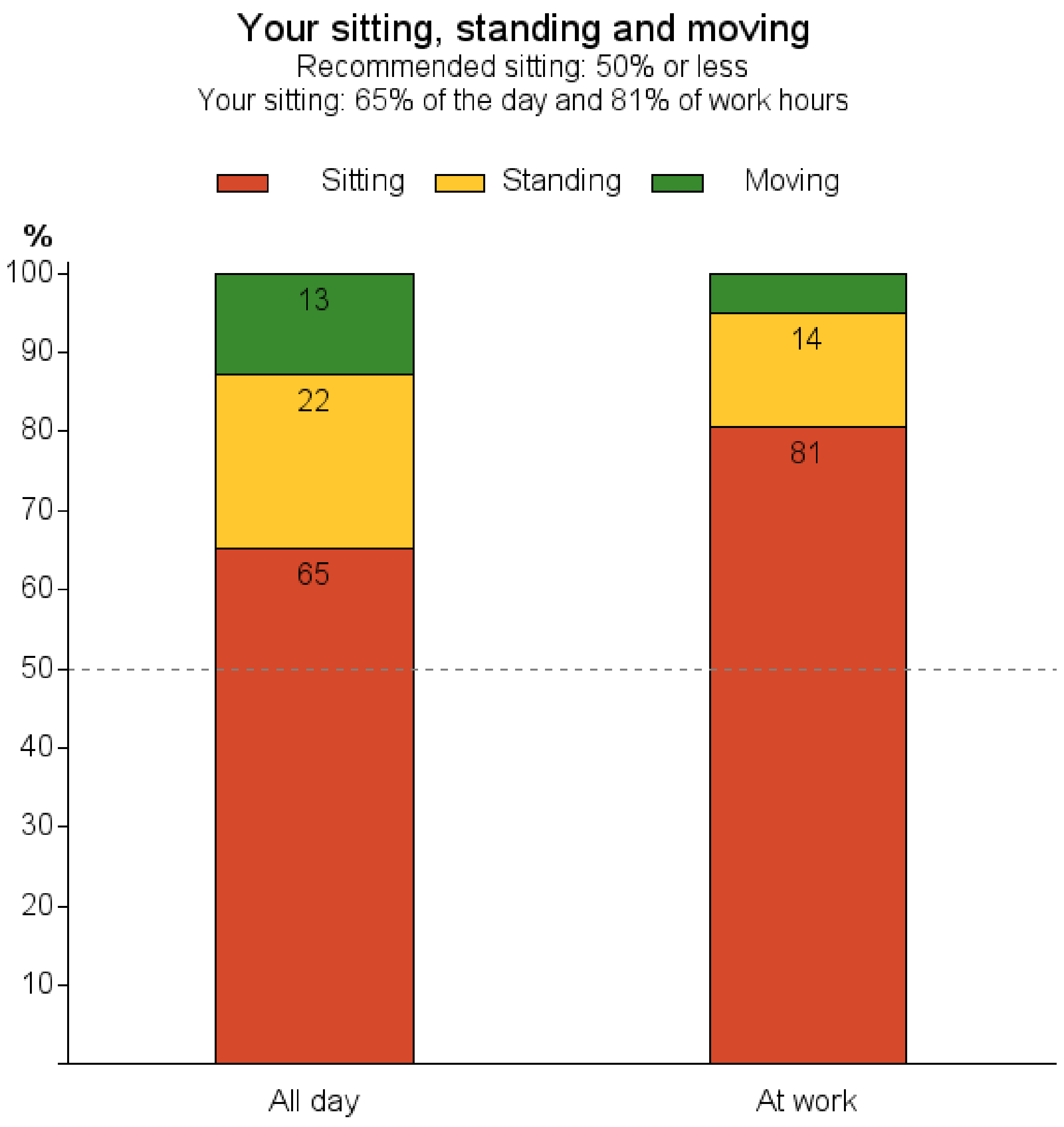

Your daily step counts

The number of steps you took each day is shown in the graph below. The recommended daily step goal for a healthy adult is 10,000 steps per day. You averaged 8908 steps per day.

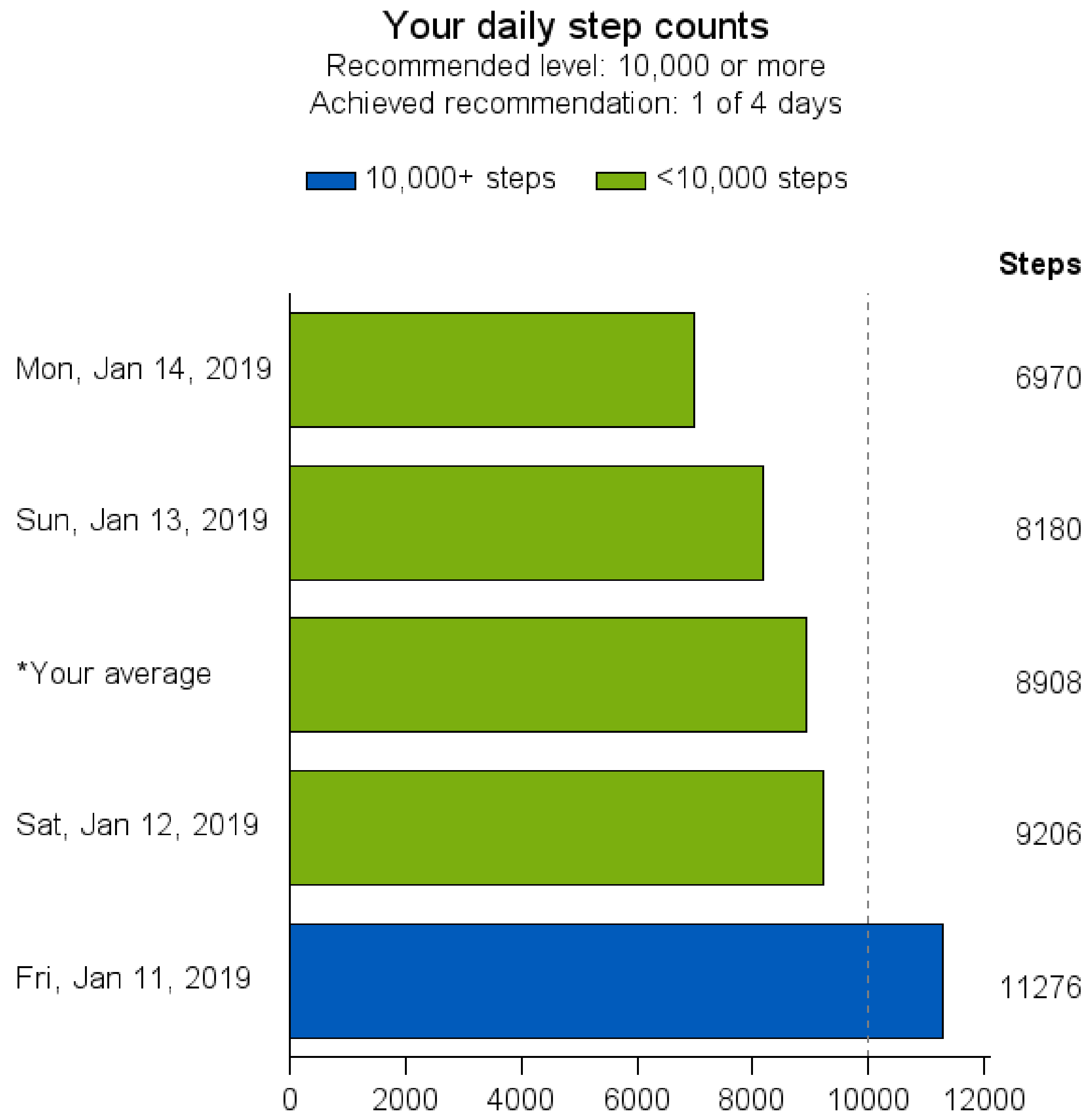

Identifying ‘danger zones’

‘Danger zones’ are areas where lots of sitting occurs throughout the day. There are two types of danger zones. **Modifiable** danger zones are during times when it is possible to replace some of the sitting with standing or moving. For example, when starting your day at work, it might be possible to swap some sitting at the desk with standing or moving. **Non-modifiable** danger zones are during times when it is not possible to replace sitting. For example, this could be during the commute to and from work when you could be stuck in peak traffic. For this study, we are focusing on the modifiable danger zones.

We will use the the graph below to help find your ‘danger zones’.

Each day’s recording is shown as a separate column, from the beginning of the day (bottom) to the end of the day (top). Times you were asleep or not wearing the monitor are not shown. The colours show what you were doing at the time: sitting (red blocks), standing (yellow blocks), or moving (green blocks). A dark red indicates you sat continuously for over 30 minutes. This type of sitting, ‘prolonged sitting’, is likely to be more harmful than other forms of sitting (shown in orange red).

By focusing on some of those bigger patches of dark red on the heatmap (your ‘danger zones’), can you recall what you were doing during the times you were sitting? Are there some opportunities for you to sit less by standing up or moving at those times? *To help you remember where you were at the time, your work hours are marked in blue.*

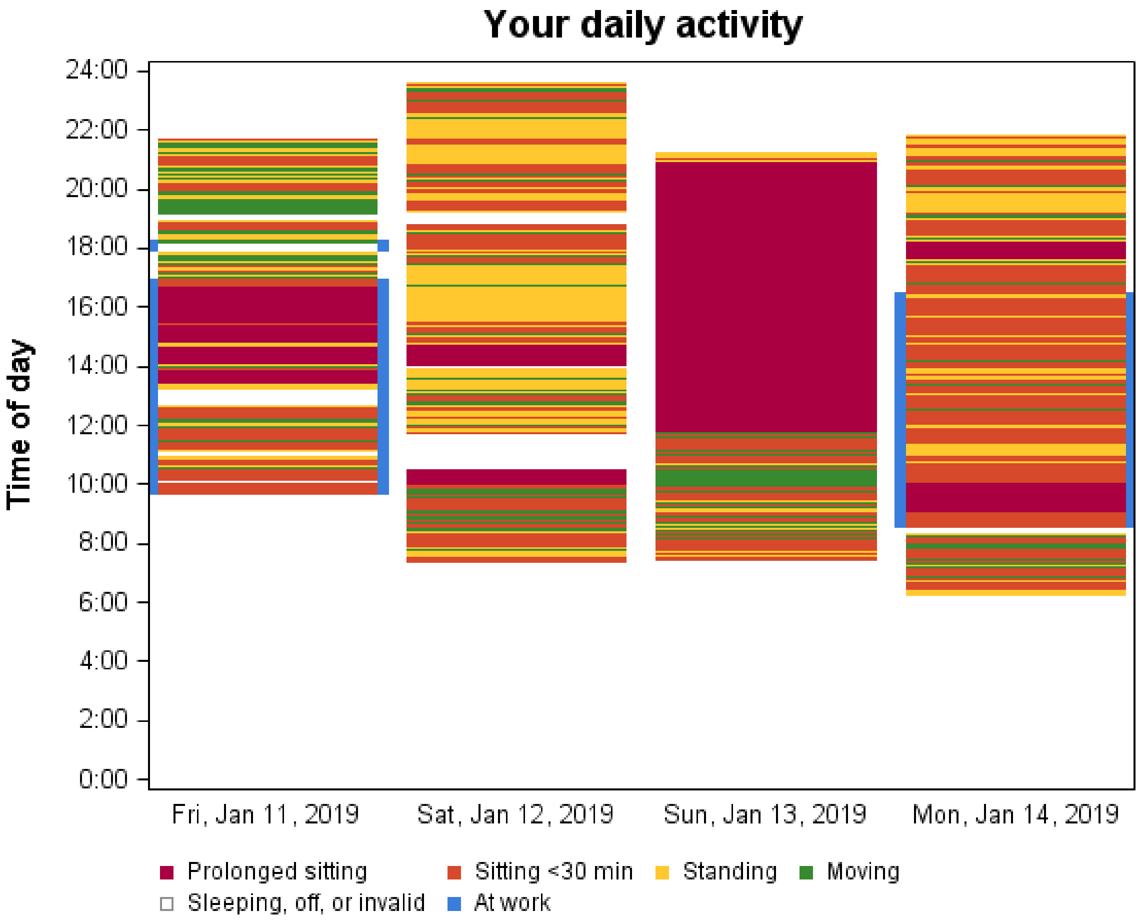

Supplement: Supplementary file 5 — Additional file 5. Example activity feedback report. [file 12889_2022_13123_MOESM5_ESM.pdf]
