# Supplementary material for: Sitting less and moving more for improved metabolic and brain health in type 2 diabetes: ‘OPTIMISE your health’ trial protocol
Source: BMC Public Health. 2022 May 10;22:929. doi: 10.1186/s12889-022-13123-x (PMC9086419; doi:10.1186/s12889-022-13123-x)
Supplement: Supplementary file 6 — Additional file 6. Intervention strategy list. [file 12889_2022_13123_MOESM6_ESM.docx]

**STRATEGIES AT WORK**

**SIT LESS AT WORK**

1. Use the sit-stand workstation in the ‘standing’ position at your desk for a certain portion of the hour (e.g. the first 10 minutes of the hour)
2. Leave your desk in the standing position when you go for breaks
3. Stand up at regular intervals (e.g. every half an hour)
4. Stand up for each phone call
5. Stand up for checking or writing emails
6. Stand when feeling tired and/or uncomfortable
7. Stand up when drinking water
8. Stand up when you visit colleagues at their desks
9. Stand up after completing a work task
10. Stand up during meetings
11. Stand up at the back of the room during presentations
12. Set a timer on your phone with Siri or Ok Google to remind you to stand up and move
13. Other: ______________________________________________________________
14. Other: ______________________________________________________________

**MOVE MORE AT WORK**

1. Set your Fitbit to remind you to move (at least 250 steps) every hour
2. iMails (walk over and talk) instead of eMails to colleagues
3. Remove bins/printers from each office and use a central one
4. Drink more water so you have to go to the water cooler (and bathroom) more often
5. Using glasses to drink water and filling up the glass more regularly
6. Walk to a bathroom that is further away
7. Step outside for fresh air
8. Use the stairs instead of a lift
9. Have lunch away from your desk
10. Go for a short walk at lunch time (tip: bring in footwear and clothing you can comfortably walk in)
11. Find a walking buddy for your lunch time walks
12. Going for a short walk after a certain task (e.g. phone call)
13. Take the longer route around the office
14. Using a printer (picking up printing) further away from your desk
15. Organise walking meetings
16. Stand up and move around during meetings
17. Check your Fitbit steps prior to leaving work, to see how many more you need to meet your daily step goal.
18. Other: ______________________________________________________________
19. Other: ______________________________________________________________

**STRATEGIES OUTSIDE OF WORK**

**SIT LESS OUTSIDE OF WORK**

1. Use your laptop on the kitchen worktop, or somewhere where you can stand
2. Stand up while on public transport, or waiting for the bus/train/tram.
3. Stand up while watching your children/grandchildren’s sporting events.
4. While watching TV, do household chores such as folding clothes, washing dishes, or ironing during commercial breaks
5. Stand up and walk around while talking on the phone
6. When sitting down while reading a book, get up every few pages.
7. Stand up while reading the morning newspaper, mail, or email
8. Move around the house when checking text messages or email on your phone
9. Wash your car by hand instead of using a drive-through car wash
10. Break up sitting time with little jobs, instead of working straight for longer periods then sitting for longer periods
11. Stand during intervals at sporting events, theatres or concerts.
12. Set a timer on your phone with Siri or Ok Google to remind you to stand up and move
13. Other: ______________________________________________________________
14. Other: ______________________________________________________________

**MOVE MORE OUTSIDE OF WORK**

1. Use an active way of commuting to and from work (walk or ride your bike)
2. Try to have reached your step goal prior to settling in for the evening
3. Join a social walking group, gym or activity class
4. Park your car further away from your work or other destination and walk a bit instead
5. Choose more active ways of commuting: cycle or walk all the way or use public transport so you have to cycle/walk to the next transport stop
6. Walk to visit neighbours instead of calling them
7. While watching TV, get off the couch and walk around the house during commercial breaks
8. Put the remote control next to the TV or on a cupboard so that it is away from where you are sitting while watching TV
9. Other: ______________________________________________________________
10. Other: ______________________________________________________________
