# Supplementary material for: Sitting less and moving more for improved metabolic and brain health in type 2 diabetes: ‘OPTIMISE your health’ trial protocol
Source: BMC Public Health. 2022 May 10;22:929. doi: 10.1186/s12889-022-13123-x (PMC9086419; doi:10.1186/s12889-022-13123-x)
Supplement: Supplementary file 7 — Additional file 7. Health coaching script. [file 12889_2022_13123_MOESM7_ESM.docx]

**OPTIMISE Health coaching (face-to-face): First**

- Estimated to take about 50-60 min
- To be conducted in a private room at the participant’s workplace to maintain confidentiality (except workstation check during #3a)
  - Note: For small office type situations, try to find the quietest and most confidential area possible
- Hard copy materials to give to participants:
  - Participant intervention handbook
  - Participant activity report
- Coach’s materials: IPad, hard copy materials listed above, health coaching script
- Coding: **Black – key points; Blue – example conversation; Purple – notes to health coach; Red - Redcap messaging**

**Important note about health coaching script**

- Below is the structure for the first session, including a suggested script. Coaches will need to balance intervention fidelity (following the structure) with a participant-centred approach. For example, at the start of the session, if the participant starts to discuss barriers to the stand-up desk that have emerged in its use (e.g., I forget to use it; I feel weird using it; people keep asking me why I got one….), it will be important to acknowledge these, noting that later in the session, they will be collectively coming up with solutions.

# INTRODUCTION

1. Coach introduces self and ensures participant is comfortable with the meeting room to chat about their involvement in the study.
2. Briefly check in on work station installation status
   - “It looks like your workstation has been successfully installed. Have you had a chance to use it yet?”
     - **To note:** They would have likely been using it, so have a brief conversation about how they’ve found it. Did they use it/like it? The longer the participant has already had the workstation prior to the consultation, the more important it is to get into a little more detail about how they went as this will be helpful for goal-setting later in the consult.
3. Explain today’s health coaching session
   - “Today we are going to review your results from the monitors, demonstrate how to use sit-stand desk, set up your Fitbit monitor, discuss some of your goals and strategies to help you meet the OPTIMISE goals, and to book in your next health coaching sessions”
   - “I will also take some notes on my iPad throughout our conversation, so I can keep track of what we discuss and send you an email summary”.
4. Explain briefly the optimise main messages/study goals: sit less and move more throughout the day
   - “Over the next six months, we are going to work together on two key things: SITTING LESS and MOVING MORE. We are working towards an overall goal of 50/50 – meaning 50% or less of your time is spent sitting and the other 50% is spent standing up or moving around.
   - We are going to help you are achieve this goal at work and also across the whole day.
   - The desk will help you sit less at work while the Fitbit will help you move more throughout the whole day.
5. Discuss with the participant their involvement with the study
   - “What has drawn you towards participating in the OPTIMISE study?”
     - **Note:** Important to establish the rationale for the participant’s involvement. This rationale will help guide impetus for change as the baseline results and goals are discussed. Keep it generic and lifestyle focused where possible so as to avoid any protracted discussion about diabetes management.

# REVIEW PARTICIPANT ACTIVITY REPORT

1. Reviews baseline feedback: sitting, standing and moving **(1^st^ graph)**
   - “This bar graph shows the hours you spend sitting, standing and moving throughout the entire day (1^st^ bar graph) and the hours you spend in these behaviours specifically during work hours (2^nd^ bar graph).

1a. Explain sitting time OVERALL and compare to average Australian

- - - On average you spent ___ hours of your overall daily waking hours sitting down which is about ____% of your time [hc1_b_1a_sen1]
    - So, currently you (are [0-50%] / are not [51-100%]) meeting the 50/50 goal for Optimise in terms of your daily sitting
  - To put this into context, the average Australian sits about 50% of their day overall.” Does this make sense to you? Is this what you expected?”
    1. **To note:** if it is not what they expected, probe more on what they thought it would come back with. The monitor also doesn’t pick up all activity – for example, if they do cycling or swimming their activity results may not be as accurate.

1b. Explain sitting time AT WORK and compare to average Australian office worker

- - “Now let’s specifically look just at your working hours”:
    - On average, you spent ___ hours per day sitting down at work which is about ____ % of your work hours.
    - When you look at the rest of your work hours, you are spending about ____ % standing and ____ % moving.
    - Office workers typically spend about 75% of their time at work sitting, so you are (higher [>80%]/ a little higher [76-80%]/ similar [75%] / a little lower [70-75%] / lower [<70%]) in comparison.
    - Thinking of our 50/50 goal, you (are achieving it [0-50]/ are close to achieving it [51-60]/ want to work gradually towards it [61-100]) in terms of your work hours.
  - Does this make sense to you? Are there any surprises?”

1. Explain ‘your daily step count’ graph (**2^nd^ graph**)
   - The monitor you wore also measured your step count. You can see here the number of steps for each day that you wore the monitor.
   - The recommended daily step goal for a healthy adult is 10,000 steps per day.
     - During the time of the assessment, you averaged ____ steps per day. / You averaged ____ steps per day and you achieved the 10,000 steps recommendation on ___ day over your ___ day assessment. / You averaged ____ steps per day and you achieved the 10,000 steps recommendation on ___ days over your ___ day assessment.) *Options for meeting the goal on 0 days 1 day and >1 day.*
   - Does this make sense to you? Are there any surprises?
2. Review ‘danger zones’ (**3^rd^ graph**)
   - This final graph is what is called a heat map. This heat map shows your activity pattern each day, with orange/red sitting, yellow ‘standing and green ‘stepping/moving’. The blue bars show the time you spent at work.
   - What we are interested here, are the big chunks of dark red here and there [point out], which indicates that you have been sitting during those times for at least 30 minutes without any interruptions. Can you remember what you were doing then? [write down].
   - Now let’s look for some other time periods where you have been a bit more active [point out green and yellow parts in heat map]. These time periods look much better, with lots of yellow and green stripes throughout the red indicating more frequent postural changes, about equal proportions of sitting and standing, and some moving [point out on heat map].
   - So the goal is to minimise those long red sitting bouts as much as possible, and increase those yellow and green stripes by having active breaks to achieve that 50/50 goal. Today we’ll talk about how to help you reach this recommendation. Does this make sense? Any questions about your feedback?

# SIT LESS AND MOVE MORE AT WORK

1. Introduction: Focus on the workplace (with goal of developing participant action plan around workplace strategies)
   - Ok – we are first going to talk about sitting less and moving more at your work place.
2. Sit less – via the desk
   - The main thing here that we are going to use to help you sit less is your sit-stand workstation. [As you have probably already worked out] – these desks allow you to continue working while you are in either a sitting or standing posture.
   - To help you [meet / keep] that 50/50 goal, I am going to ask you to set a target amount you are going to try and stand at your desk each day at work over the next week. The point here is to be realistic and start off gradually so your body can get used to using the workstation.
   - So – what would you like to work towards over the next week in terms of standing at your desk? Record this target in [**hc1_c_2_goal]**
     1. Note: This discussion is not necessarily posited to be quantitative in nature. Important to get a feeling for how the participant will best like an improvement to be expressed: whether by percentage or by an hour / min reduction and then help them to establish a clear and simple goal around sitting reduction.
     2. If participant struggles to know where to start, suggest that they aim for a 30 – 60 minute reduction per day by standing at their new workstation.
     3. Note: Coach takes notes of the target participants selected – checks in that they are happy with that target; cautions against too big a reduction in sitting / increase in standing; and emphasises that regular breaks are important too in reaching their goal.
   - There are also many ways you could sit less at work that don’t need to involve the desk. Sitting can be very habitual, so it is good to use strategies that are linked to either certain times of the day or events that happen regularly.
   - Here are some common strategies that people have used to help them to sit less at work [show strategy list]. Do any of these sound like ones you would like to try? Do you have any other ideas that you would like to use to sit less? [If choose lots of strategies] That is great that you have chosen lots of strategies - which ones would you like to focus on for the next week?
     1. Coach records strategies in [**hc1_c_2_strategies]**.
     2. Need to choose at least 1 sit less at work strategy
     3. Ensure where possible that strategies chosen employ the SMART framework: **S**pecific, **M**easurable, **A**chievable, **R**elevant, and **T**ime-based goals. Coach records strategies in [**hc1_c_2_smartgoals]**.
3. Move more – via the fit bit
   - The other element we want to work on is moving more – and the fitbit is going to help you do that.
   - Did you get a chance to watch the short YouTube clips about why moving more is important?
   - [Summarise clips even if they have]: So you may remember that regularly moving those large muscles of the body, particularly the legs is important for helping the body to clear blood glucose from circulation. To put simply, the larger the muscle being used (for example the legs over the arms), the more glucose gets absorbed by that muscle. We need to make sure we are moving regularly, and not just at the start of end of the day, so that we can always clear the glucose from circulation and get the glucose into the muscles.

3a) ‘Active breaks’

- - “What is important here is the regularity. This means to try and move at least every hour, even if it is just for a couple of minutes, as research has shown that even 2-3 minutes of moving around can have many benefits. In this program we are calling these “active breaks”.
  - There are two types of activities that we will encourage you to try: 1) walking/stepping and 2) Simple Resistance Activity (SRA) breaks.

<show/explain SRAs> - also show briefly again at the end of the session when you go back to the desk

- - How does this sound? Do you think you can take at least one active break per hour while you are at work? How many would you like to aim for in the first week? Record activity break [**hc1_c_3a_goal**]
    - Note: The aim is 1 active break per hour, but the participant can do as many as they like.
    - There may be some barriers raised – active breaks can happen at the desk – here encourage to record them (like you are going to show them below).

3b) Download Fitbit app on participant’s phone and connect fitbit to Fitabase

- - The Fitbit that I am preparing for you now has an alert system that will prompt you to take these active breaks. I am also going to show you how to set prompts every hour to help you more move. If you forget how to do this, here is an instruction sheet to show you how [participant can read through the sheet while the fitbit is being set up]
    1. Note: username and password already set up previously. Username optimise+s***tudyID***@gmail.com, Password: optimise***studyID***
    2. Note: important for coach to explain to participant that to achieve a ‘Fitbit break’ they must reach 250 steps within the hour – equivalent to a 2-3 minute light walk. If they haven’t reached this goal, they will receive a reminder at the 50 minute mark (a vibration)’.
    3. Note: Participant to select their own timeframe for when prompt occur (e.g. 7am to 7pm) Record timeframe [**hc1_c_3a_timeframe]**

3c) Strategies for move more at work

- - The Fitbit should be a good prompt to remind you to move more, but there are also other strategies you can use to get more moving in across your work day [SHOW STRATEGY LIST]. Do any of these sound like ones you would like to use? Do you have any other ideas on how you can get more moving into your work day? [If choose lots of strategies] That is great that you have chosen lots of strategies - which ones would you like to focus on for the next week?
    1. Coach records strategies in [**hc_c_3c_strategies]**
    2. Need to choose at least 1 move more at work strategy
    3. Ensure where possible that strategies chosen employ the SMART framework: **S**pecific, **M**easurable, **A**chievable, **R**elevant, and **T**ime-based goals. Coach records strategies in [**hc1_c_3c_smartgoals]**.

# SIT LESS AND MOVE MORE ACROSS THE DAY

1. Overview on whole of day
   - Now that we have strategies specifically for work we are going to discuss strategies for how you can meet your 50/50 target across the whole day.
     - During the time of the assessment, you were currently sitting about ___% of your day, and averaging around ____ steps per day
2. Touch on the heat maps again and point out danger zones
   - Let’s revisit those danger zones in the heat maps to discuss opportunities where you might be able to sit less and move more.
   - Is it feasible here to replace these big red chunks of sitting with an active break or simply standing?
     - Ensure participant selects appropriate timeframes and not during transit (e.g. driving)
   - What strategies do you think you can do to help you sit less when you are in those danger zones?
     - Coach records strategies in [**hc1_d_2_strategies_1]**
     - Discuss possible barriers / strategies to address barriers depending on what they are doing in those time frames
     - Ensure where possible that strategies chosen employ the SMART framework: **S**pecific, **M**easurable, **A**chievable, **R**elevant, and **T**ime-based goals. Coach records strategies in [**hc1_d_2_smartgoals]**.
3. Moving more across the whole day
   - By moving more you should also notice a change to the number of steps you do. It can be really helpful to set a target for how many steps you want to achieve each day remembering it is good to start off gradually!
   - How many steps would you like to reach each day? Coach records step goal [**hc1_d_3_goal]**
     - What are some of the strategies you could do to help you increase your steps? Coach records strategies in [**hc1_d_3_strategies]**
   - Don’t forget, the Fitbit watch will help you to sit less and move more with the use of ‘hourly’ activity prompts, as well as tracking your steps.
     - Note: If the participant has any trouble coming up with a figure or achievable goal recommend: “To start off, we suggest a 10% increase in steps per day. Do you think you could do this for the first week? More? Less?”
     - Note: If necessary for the participant, cue the following: “As an approx. guide, it takes 1 minute to walk 100 steps, so it would take 5 minutes to walk an extra 500 steps, 10 minutes to walk 1,000 steps (and so forth). “
     - Completing regular Fitbit breaks will help you to achieve more steps throughout the week.
     - Note: coach to note to participant that the thigh monitor steps may differ to the Fitbit steps
     - Coach and participant to go through strategies, ensuring participant selects the strategies. As a minimum, encourage participant to select at least 1 Move More strategy for work and non-work times.
     - Ensure where possible that strategies chosen employ the SMART framework: **S**pecific, **M**easurable, **A**chievable, **R**elevant, and **T**ime-based goals. Coach records strategies in [**hc1_d_3_smartgoals]**.

# REVIEW READINESS AND ACTION PLAN

1. Review readiness
   - We’ve covered quite a bit already. What do you think about this focus on sitting less and moving more at work? And across the whole day? [It is very important here to get a sense of the participant’s level of interest and motivation. Are they taking part simply because they have to? Are they actually keen?]
   - On a scale of 1-10 how would you rate your readiness/confidence to change your habits and enact these strategies throughout the day? Is there anything you would change? Coach records this [sliding bar]
     1. Note: Make sure to reflect/ paraphrase what participant answers here. If 5 or less, need to explore why they’re taking part. If 6-7, what is keeping them from feeling more strongly ready? Any particular barriers/negative things they are expecting to happen? They might answer that they are happy to give it a go, but as they have never tried to work at a workstation like this; they simply have no idea how they will go and hence are sceptical. In this case the consultant could normalize, e.g. ‘Yes, that is absolutely normal/understandable, in fact a lot of participants feel like that in the beginning etc’; If 8 – 10, ‘great, sounds like you’re ready to give it a go’. If different in different settings, talk it through with them]
2. Review action plan
   - You have chosen some great strategies to help you sit less and move more across the day. At work you are going to focus on X and X. While across the whole day you are going to focus on X and X. Does that sound okay? Anything else that you would consider here?
     1. Check in that they are happy with those strategies or if they wanted to achieve anything else.
3. Review barriers for intervention uptake
   - Do you foresee anything that would get in the way of the goals you have set for the coming week?
4. Encourage to stick to your goals but listen to your body
   - Over the next week, try to use your strategies to shift regularly between sitting and standing at your workstation, and see how often you can take an active break. But remember that you are about to change a sitting habit you have probably had for quite some time. So listen to your body – if you feel you are getting stiff or feel uncomfortable (a little in the beginning is normal), change your posture or sit back down for a little while.

# BOOKING PHONE CALLS

1. Booking in future phone call appointments
   - In order to monitor your commitment to the action plan we will check in with the progress of your goals with weekly scheduled phone calls. These calls will be about 5- 10 minutes to see how you are going. We will also keep building on the strategies that you have chosen today.
   - So for the next three weeks I will call you every week, then after that I will call you every 3 weeks until our study completes at 21 weeks.
   - Let’s look at your calendar and book in the first few phone calls over the next coming weeks. What day/time would suit for me to call you?
     1. Note: Phone call schedule is: Week 1, 2, 3, 6, 9, (12 is at Baker), 15, 18 and 21.
   - We will also catch up for another face to face session in three months’ time at your next visit to the Baker Clinic which is on the (DATE).

# DEMONSTATIONS AT DESK

1. Review correct ergonomic usage
   - Let’s have a look at your standing posture: right angle of upper to lower arms; even wrists (keyboard flat?); monitor 15-50 degrees below viewing line (head straight and not tilted); body straight but comfortable.
     - Note: show ergonomic section in folder / provide the laminated copy
2. Demonstrate the active breaks at the desk

**ADMIN**

Email summary of goals, strategies selected, and comments made during the coaching session.

- - 1. Note: If participant prefers not to receive emails, organise how they would like to receive it (i.e. via post to their home).
