# Supplementary material for: Sitting less and moving more for improved metabolic and brain health in type 2 diabetes: ‘OPTIMISE your health’ trial protocol
Source: BMC Public Health. 2022 May 10;22:929. doi: 10.1186/s12889-022-13123-x (PMC9086419; doi:10.1186/s12889-022-13123-x)
Supplement: Supplementary file 8 — Additional file 8. COVID-19 Snapshot Questionnaire. [file 12889_2022_13123_MOESM8_ESM.docx]

**COVID19 Snapshot**

*Key: Italics = text that does not appear on the survey ○ Radio / dropdown – single selection □ Radio / dropdown – multiple selections _______ Enter response (numeric, date, text)*

Thank you for taking the time to fill in this survey, which is expected to take about 10-15 minutes to complete. This survey asks questions about your work, sitting, standing and moving to help better understand the situation and needs of desk-based workers during the COVID-19 pandemic.

Privacy and confidentiality

The information you provide in the survey is completely confidential. All survey responses will be stored using the Baker Institute-approved data management tool called Research Electronic Data Capture ('REDCap').

Instructions

If possible, please answer all the questions. After reading the questions carefully, please either select the most suitable option or enter an answer the best you can.

COVID-19 Timelines

This survey asks for a picture of now versus 'before' the COVID-19 pandemic. In responding, please select whatever timeframe for 'before' COVID-19 makes sense to you, such as before:

COVID disrupted your work or domestic situation Social distancing was introduced (21 March in Australia) National restrictions on non-essential gatherings (29 March in Australia)

**YOUR WORK**

How many hours a week did you normally work in your job before COVID-19? ____________

How many hours a week do you currently work? _____________________

*(Asked if current hours = 0)* Please indicate why you normally work 0 hours currently

- On leave at the moment
- I no longer have a job
- Prefer not to disclose

**When the remaining questions ask about working 'currently', please respond about whenever you were last working during the COVID-19 pandemic.**

Which of the following best describes where you work currently?

- Work from home
- Mostly work from home (sometimes at the workplace)
- About equally from home and the workplace
- Mostly at the workplace (sometimes from home)
- At the workplace

Which of the following best describes where you usually worked before COVID-19?

- Work from home
- Mostly work from home (sometimes at the workplace)
- About equally from home and the workplace
- Mostly work at the workplace (sometimes from home)
- At the workplace

**Work-life balance**

Do you currently have flexible work hours (choice in when you work)?

- Yes
- No

How does the flexibility of your work hours compare now versus before COVID-19?

- About the same
- My hours were more flexible BEFORE
- My hours are more flexible NOW

How would you compare your workload now with before COVID-19?

- I have much more work to do now
- I have slightly more work to do now
- I have about the same amount to do as before
- I have slightly less work to do
- I have much less work to do

What are your caring responsibilities like now compared with before COVID-19?

- Much more now
- Slightly more now
- About the same
- Slightly less now
- Much less now
- Not applicable - I do not have any caring responsibilities

Feel free to provide any further comments here about any changes in your work you have experienced (or expect to experience) related to COVID-19. ________________________________________________________________

**YOUR WORK ENVIRONMENT**

**The next questions ask you to think about your main workspace (where you mostly work).**

**If you think a question is not applicable to you because you have not changed where you**

**work, you can select the NA (not applicable) option. Or if some changes have happened**

**around you (such as noise) you can tell us about those.**

In your opinion, what is your main workspace / work environment like now versus before COVID-19?

|  | NA – Environment is unchanged | Much worse now | Slightly worse now | About the same | Slightly better now | Much better now |
| --- | --- | --- | --- | --- | --- | --- |
| Computer set-up | ○ | ○ | ○ | ○ | ○ | ○ |
| Desk | ○ | ○ | ○ | ○ | ○ | ○ |
| Chair | ○ | ○ | ○ | ○ | ○ | ○ |
| Noise | ○ | ○ | ○ | ○ | ○ | ○ |
| General distractions | ○ | ○ | ○ | ○ | ○ | ○ |
| Lighting | ○ | ○ | ○ | ○ | ○ | ○ |
| Your physical comfort | ○ | ○ | ○ | ○ | ○ | ○ |
| Satisfaction with your workspace | ○ | ○ | ○ | ○ | ○ | ○ |
| Satisfaction with your work environment | ○ | ○ | ○ | ○ | ○ | ○ |

Please rate your access to each of these necessary work resources now versus before COVID-19?

|  | NA – Environment is unchanged | Much worse now | Slightly worse now | About the same | Slightly better now | Much better now |
| --- | --- | --- | --- | --- | --- | --- |
| Quality internet | ○ | ○ | ○ | ○ | ○ | ○ |
| Electronic resources (files, software etc.) | ○ | ○ | ○ | ○ | ○ | ○ |
| Physical resources (pens, copying etc.) | ○ | ○ | ○ | ○ | ○ | ○ |

**Sitting and standing at your computer**

My computer set-up at home is suitable for...

(Computer includes any tablet / laptop you might use to work)

- Working either sitting or standing
- Working sitting down
- Working standing up
- Not applicable – no computer at home

My computer set-up at the workplace is suitable for...

(Computer includes any tablet / laptop you might use to work)

- Working either sitting or standing
- Working sitting down
- Working standing up
- Not applicable – no suitable device at the workplace

**Your joints and muscles**

What level of discomfort do you have currently in comparison to before COVID-19?

(Discomfort includes pain, aching, burning, numbness or tingling.)

|  | Never had discomfort | Much worse | Slightly worse | Similar | Slightly better | Much better |
| --- | --- | --- | --- | --- | --- | --- |
| Upper back, neck, shoulders, elbows, wrists or hand | ○ | ○ | ○ | ○ | ○ | ○ |
| Lower back | ○ | ○ | ○ | ○ | ○ | ○ |
| Hips, thighs, buttocks, knees, ankles or feet | ○ | ○ | ○ | ○ | ○ | ○ |

Please feel free to provide below any comments about your workspace / work environment and its relevance to your muscle and joint health.

________________________________________________________________________________

**YOUR SITTING STANDING AND MOVING**

**During work hours** How would you describe your typical workday currently? (During your work hours only, not counting computing or leisure time.) Please answer as a percentage (0-100) without the % sign, and make sure the total adds up to 100%.

? % Sitting: ______

? % Standing: ______

? % Moving: ______

TOTAL % : ______ (Total must add up to 100%)

And, how would you describe your typical workday before COVID-19? (As before, please answer as a percentage 0-100 without the % sign)

? % Sitting: ______

? % Standing: ______

? % Moving: ______

TOTAL % : ______ (Total must add up to 100%)

**Your sitting, standing and moving outside work**

How much of the following activities are you doing outside your work hours now versus before COVID-19?

|  | Much more now | Slightly more now | About the same | Slightly less now | Much less now |
| --- | --- | --- | --- | --- | --- |
| % of time spent moving | ○ | ○ | ○ | ○ | ○ |
| % of time spent standing | ○ | ○ | ○ | ○ | ○ |
| % of time spent sitting |  |  |  |  |  |
| % of sitting occurring in long periods of >30 min | ○ | ○ | ○ | ○ | ○ |
| How much you sit watching TV, movies etc. | ○ | ○ | ○ | ○ | ○ |
| How much you exercise | ○ | ○ | ○ | ○ | ○ |

**Motivation to sit less and move more**

Please rate your levels of motivation to do each of the following behaviours, now versus before COVID-19.

|  | Much more now | Slightly more now | About the same | Slightly less now | Much less now |
| --- | --- | --- | --- | --- | --- |
| Sit less during work hours | ○ | ○ | ○ | ○ | ○ |
| Move more during work hours | ○ | ○ | ○ | ○ | ○ |
| Sit less outside of work | ○ | ○ | ○ | ○ | ○ |
| Move more outside of work | ○ | ○ | ○ | ○ | ○ |
| Exercise | ○ | ○ | ○ | ○ | ○ |

**Physical activity**

Please check the boxes below for all the activities you are now doing more (or started doing)

since COVID-19 and all activities that you are now doing less (or stopped doing). Any activity

you are still doing (or not doing) the same as before, please just leave unchecked**.**

|  | Doing more or started doing | Doing less or stopped doing |
| --- | --- | --- |
| Walking indoors | □ | □ |
| Running / jogging indoors | □ | □ |
| Walking outdoors | □ | □ |
| Bushwalking | □ | □ |
| Running / jogging outdoors | □ | □ |
| Swimming | □ | □ |
| Cycling indoors (e.g., stationary bike) | □ | □ |
| Cycling outdoors | □ | □ |
| Aerobics / fitness class or similar | □ | □ |
| Yoga, pilates or similar | □ | □ |
| Resistance exercise / weights | □ | □ |
| Organised sports (not already mentioned) | □ | □ |
| Other | □ | □ |

*(Asked if selected doing more ‘Other’)* Which other activities are you now doing more (or started doing)? _________________________________________

*(Asked if selected doing less ‘Other’)* Which other activities are you now doing less (or stopped doing)? _________________________________________

**YOU HAVE NOW COMPLETED THE SURVEY**

Thank you very much for completing the COVID-19 survey.

To submit your survey responses, please click the submit button below.
