# Supplementary material for: Sitting less and moving more for improved metabolic and brain health in type 2 diabetes: ‘OPTIMISE your health’ trial protocol
Source: BMC Public Health. 2022 May 10;22:929. doi: 10.1186/s12889-022-13123-x (PMC9086419; doi:10.1186/s12889-022-13123-x)
Supplement: Supplementary file 9 — Additional file 9. Questionnaire measures. [file 12889_2022_13123_MOESM9_ESM.docx]

| Table 4: Questionnaire measures collected in OPTIMSE Study Participants in OPTIMISE Surveys (unless other source indicated) | | | |  |
| --- | --- | --- | --- | --- |
| **Measures** | | | **Instrument / reference** | **Validity & reliability (if applicable)** |
| **Confounders / mediators / moderators / other measures** | Socio-demographics at baseline^a^ | Ethnicity, Household type, Education, Occupation | Adapted from AusDiab (1) |  |
|  |  | Age, Gender **(Eligibility Survey)** |  |  |
|  | CVD history at baseline (Health History Checklist)^a^ | History yes/no of: Angina; Heart attack; Heart bypass operation; Stroke; Angioplasty for peripheral vascular disease, retinal damage and kidney damage due to diabetes, nerve damage, and lower limb amputation | Adapted from AusDiab |  |
|  | Diabetes complications at baseline (Health History Checklist)^a^ | History yes/no of: kidney damage from diabetes; eye or retinal damage from diabetes; nerve damage from diabetes; numbness, burning or tingling in feet; foot ulcer; lower limb amputation | Adapted from AusDiab |  |
|  | Women's health (Menopause status survey)^c^ | Age at menarche (baseline)^a^ |  |  |
|  |  | Diagnosed (yes/no) with: PCOS; Premature ovarian failure, Hypothalamic amenorrhea; Oesterogenic malignancy; Endometriosis |  |  |
|  |  | Menopausal status (pre, peri- and post-menopausal), Time since menopause, Cause of menopause (natural / medical) | Created considering STRAW10+ recommendations | Harlow SD, Mitchell ES, Crawford S, Nan B, Little R, Taffe J, et al. The ReSTAGE Collaboration: defining optimal bleeding criteria for onset of early menopausal transition. Fertil Steril 2008 (2) |
|  |  | Hormonal contraceptive use, hormonal contraceptive type |  |  |
|  |  | Hormonal therapy use |  |  |
|  |  | Menopausal symptoms (3 items) | from Greene Clinimetric scale (3) |  |
|  | Workplace Environment at baseline^a^ | Office layout (open plan / closed plan / mixed) | BeUpstanding adaptation of Checklist of Health Promotion Environments at Worksite (4) |  |
|  |  | Whether and which staff have height-adjustable desks available (2 items) |  |  |
|  |  | Presence of environmental supports: Resource / activity promotion (10 items), Policy / culture (8 items), and Physical Environment (14 items) |  |  |
|  | Work factors | Hours worked & days worked over last 7 days | Developed for this study |  |
|  |  | Work location (home / mostly home / equally workplace & home / mostly workplace / workplace)^b^ |  |  |
|  |  | Perceived workload 1-10 score: work (1 item)^b^ and caring responsibility^b^ (1 item) | Created based on Borg Workload Scale and NASA Task Load Index (5) | Almghairbi DS, Marufu TC, Moppett IK. Anaesthesia workload measurement devices: qualitative systematic review. BMJ Simul Technol Enhanc Learn 2018 (6) |
|  | Dietary Intake | Total intakes of energy, maronutrients & micronutrients, % contribution to energy of core and non-core foods, Australian Recommended Food Score (ARFS) **(Diet Survey)** | University of Newcastle’s Australian Eating Survey | Collins CE, Boggess MM, Watson JF, Guest M, Duncanson K, Pezdirc K, et al. Reproducibility and comparative validity of a food frequency questionnaire for Australian adults. Clin Nutr. 2014 (7) |
|  | Medication Use | Staff verified usage, dose and frequency of all OHAs, prescription and over-the-counter medications taken for blood pressure, lipids, mental health, sleep, and other reasons **(Medication Usage Audit)** | Developed for this study |  |
|  | Receipt of other interventions ^c^ | Use of other (non-OPTIMISE) trackers: used other wearables (yes/no), used smartphone / app (yes/no), duration used; features used (describe); prompt use frequency; prompt usage location (home / work /other) **(Personal Wearables Survey)** | adapted from CL Brakenridge et al. 2018 (8) |  |
|  |  | Number of visits to allied health care worker in last 3 months: Aboriginal Health Worker; Audiologist; Chiropractor; Diabetes Educator; Dietitian; Exercise Physiologist; Mental Health Worker; Occupational Therapist; Osteopath; Physiotherapist; Podiatrist; Speech Pathologist | Developed for this study |  |
|  | PA & sitting behaviours and attitudes | Physical activity frequency & duration: walking; gardening; other moderate; vigorous; strength training (total moderate, vigorous, moderate-vigorous PA) | Active Australia Survey | Australian Institute of Health and Welfare (AIHW) 2003. The Active Australia Survey: a guide and manual for implementation, analysis and reporting. Canberra: AIHW. (9) |
|  |  | Sitting time on weekdays (Monday – Friday) and weekends (Saturday and Sunday) across different domains: during job; for travel; with TV or screen device for recreation or entertainment; with screen device for other recreational purposes (e.g. gaming, social media); screen device for information, reading and responding to emails, pay online bills or complete online chores; other sitting time. (12 items) | Adapted from AusDiab |  |
|  |  | Reported % of workday sitting, standing, walking, heavy labour | OSPAQ | Maes I, Ketels M, Van Dyck D, Clays E. The occupational sitting and physical activity questionnaire (OSPAQ): a validation study with accelerometer-assessed measures. BMC Public Health 2020 |
|  |  | Desired % of workday sitting, standing, moving & gap scores (desired % - actual %) | Adapted from Wallmann-Sperlich et al. 2017 (10) |  |
|  |  | Reported % sitting time spent in prolonged sitting | Adapted from Clark et al. 2021 (11) |  |
|  |  | Transport time to & from work min/week: driving, public transport, walking, cycling | Sedentary, Transport and Activity Questionnaire | Mensah K, Maire A, Oppert JM, Dugas J, Charreire H, Weber C, et al. Assessment of sedentary behaviors and transport-related activities by questionnaire: a validation study. BMC Public Health 2016 (12) |
|  |  | Extent of work sitting (Likert response) due to: computer, phone use, driving, meetings, manual work, other reasons | Adapted from BeUpstanding trial |  |
|  |  | Use (Never-Always + NA) of 15 strategies to sit less | Adapted from BeUpstanding trial |  |
|  |  | Sitting knowledge score (5 items) | Adapted from Hadgraft et al. 2017 (13) |  |
|  |  | Perceived organisational norms regarding sitting /activity score 1-5 (4 items) | Adapted from Hadgraft et al. 2017 |  |
|  |  | Social support scores: exercise & sitting (14 items), exercise (6 items), sitting (4 items), | adapted from Sallis' Social Support for Exercise Score (14) | Sallis JF, Grossman RM, Pinski RB, Patterson TL, Nader PR. The development of scales to measure social support for diet and exercise behaviors. Prev Med 1987 |
|  |  | Barriers to physical activity score (8 items) | Adapted from SUV (15) |  |
|  |  | Barriers to sitting less score (6 items) | Adapted from BeUpstanding trial |  |
|  |  | Physical activity self-regulation (0-36) score (9 items) | Developed for this study |  |
|  |  | Sitting self-regulation (0-36) score (9 items) | Adapted from SUV trial |  |
|  |  | Motivation for Physical Activity (24 items): measuring relative autonomy index; external regulation; introjected regulation; identified regulation; integrated regulation; intrinsic regultion **(Motivation for Physical Activity)** | adapted from Behavioural Regulation in Exercise Questionnaire (BREQ-3) (16) | Wilson PM, Rodgers WM, Loitz CC, Scime G. “It’s Who I Am … Really!’ The Importance of Integrated Regulation in Exercise Contexts1. J Appl Biobehav Res 2006 |
|  |  | Motivation to break up sedentary behaviour (SB Motivation Survey ^b^): relative autonomy index; external regulation; introjected regulation; identified regulation; integrated regulation; intrinsic regultion **(Motivation to Break up Sitting Survey)** | adapted from BREQ-3 | Wilson PM, Rodgers WM, Loitz CC, Scime G. “It’s Who I Am … Really!’ The Importance of Integrated Regulation in Exercise Contexts1. J Appl Biobehav Res 2006 |
|  | COVID-19 | COVID-19 changes to commuting | Developed for this study |  |
|  |  | COVID-19 limitations (3 items): manage diabetes; sit desired amount; move desired amount | Developed for this study |  |
| **Secondary and exploratory outcomes** | Self-report behavioural outcomes | Domain-specific sitting time: collecting for weekdays, weekend days min/week in domains of work, travel, tv, recreational computer, non-recreational computer, other domains | Multi-context sitting questionnaire | Clark BK, Lynch BM, Winkler EA, Gardiner PA, Healy GN, Dunstan DW, et al. Validity of a multi-context sitting questionnaire across demographically diverse population groups: AusDiab3. Int J Behav Nutr Phys 2015 (17) |
|  |  | Perceived changes (5-point likert scale + NA do not do) in 16 domains of sitting over the last 6 months **(6M OPTIMISE Survey & 12M OPTIMISE Survey^d^)** | New measure, domains based on Gomersall et al 2019 (18) |  |
|  | Work outcomes (baseline & followup) | Absenteeism (sick days over last 3 months) | Work Productivity and Activity Impairment Questionnaire | Reilly MC, Zbrozek AS, Dukes EM. The validity and reproducibility of a work productivity and activity impairment instrument. Pharmacoeconomics 1993 (19) |
|  |  | Work satisfaction  (1-7) score (1 item) | Adapted from SMArT Work (20) | Nagy MS. Using a single-item approach to measure facet job satisfaction. J Occup Organ Psychol 2002 (21) |
|  |  | Self-rated work performance (1-7) score (1 item) | Adapted from SMArT Work |  |
|  |  | Work engagement (0-6) score (3 items) | Utrecht Work Engagement Scale (UWES-3) | Schaufeli WB, Shimazu A, Hakanen J, Salanova M, De Witte H. An ultra-short measure for work engagement: The UWES-3 validation across five countries. European Journal of Psychological Assessmen 2019 (22) |
|  |  | Work productivity: caputuring Productivity loss scores, Time Demands, Physical Demands, Mental/Interpersonal Demands, and Output Demands | Work Limitations Questionnaire | Lerner D, Amick III BC, Rogers WH, Malspeis S, Bungay K, Cynn D. The work limitations questionnaire. Med Care. 2001 (23) |
|  | Health & Wellbeing | Depression (7 items), Anxiety (7 items), Depression/Anxiety score (14 items) | Hospital Anxiety and Depression Score | Herrmann C. International experiences with the Hospital Anxiety and Depression Scale--a review of validation data and clinical results. J Psychosom Res 1997 (24) |
|  |  | Diabetes distress score (20 items) | Problem Areas in Diabetes-20 | Hermanns N, Kulzer B, Krichbaum M, Kubiak T, Haak T. How to screen for depression and emotional problems in patients with diabetes: comparison of screening characteristics of depression questionnaires, measurement of diabetes-specific emotional problems and standard clinical assessment. Diabetologia 2006 (25) |
|  |  | Fatigue Severity Score (4 items), Fatigue Interference Score (10 items) | Fatigue Symptiom Inventory | Hann DM, Jacobsen PB, Azzarello LM, Martin SC, Curran SL, Fields KK, et al. Measurement of Fatigue in Cancer Patients: Development and Validation of the Fatigue Symptom Inventory. Qual Life Res 1998 (26) |
|  |  | Musculoskeletal symptoms over last 3 months & pain (0-10 rating) over last 7 days: collecting data re neck, shoulders, elbows/wrists, upper back, lower back, hips, knees, ankles/feet (16 items) | Modified Nordic Questionnaire and scale adapted from Andersen et al. 2012 (27) | Palmer K, Smith G, Kellingray S, Cooper C. Repeatability and validity of an upper limb and neck discomfort questionnaire: the utility of the standardized Nordic questionnaire. Occup Med 1999 (28) |
|  |  | Self-reported sleep duration & quality: collecting data on self-rated sleep quality (1 item), time in bed, sleep duration, latency, and efficiency (from times into bed, lights out, wake up, out-bed, number & duration of awakenings) **(10 consecutive daily recalls in Activity Monitor Log)** | Consensus Sleep Diary | Dietch JR, Taylor DJ. Evaluation of the Consensus Sleep Diary in a community sample: comparison with single-channel electroencephalography, actigraphy, and retrospective questionnaire. J Clin sleep Med JCSM 2021 (29) |
|  | Quality of Life (Quality of Life Survey) | Physical QoL T score & Psychosocial QoL T score | Australian Quality of Life Survey - 8D Multi-Attribute Utility Instrument | Richardson J, Khan MA, Iezzi A, Maxwell A. Measuring the sensitivity and construct validity of 6 utility instruments in 7 disease areas. Medical Decision Making 2015. Richardson J, Sinha K, Iezzi A, Khan MA. Modelling utility weights for the Assessment of Quality of Life (AQoL)-8D. Qual life Res. an Int J Qual life Asp Treat care Rehabil 2014 (30) |
|  | Adverse Events | All, intervention-induced, serious, serious and caused by intervention (researcher coded). Measured from new health problems in last 6 months (yes/no); description; severity; belief of likelihood due to intervention; duration (6 month OPTIMISE questionnaire) plus symptom, date of onset and resolution, intensity of symptom, action taken  (none, added or removed medication or changed dose), study withdrawal, withdrawal from intervention, relationship of event to study, outcome (resolved, resolved with seleque, not resolved). | adapted from Eakin et al. 2010 (31) |  |
| Measures are collected at all assessments  ^a^ Baseline only  ^b^ added partway through trial due to COVID-19  ^c^ added partway through trial for other reasons (e.g. with introduction of the expanded protocol, new substudy included)  ^d^ initially asked of intervention only, extended to delayed intervention controls partway through study | | | | |

References

1. Dunstan DW, Zimmet PZ, Welborn TA, Cameron AJ, Shaw J, de Courten M. The Australian Diabetes, Obesity and Lifestyle Study (AusDiab)-methods and response rates. Diabetes Res Clin Pr. 2002;57:119–29.

2. Harlow SD, Mitchell ES, Crawford S, Nan B, Little R, Taffe J. The ReSTAGE Collaboration: defining optimal bleeding criteria for onset of early menopausal transition. Fertil Steril. 2008 Jan;89(1):129–40.

3. Greene JG. A factor analytic study of climacteric symptoms. J Psychosom Res. 1976;20(5):425–30.

4. Healy GN, Goode AD, Abbott A, Burzic J, Clark BK, Dunstan DW, et al. Supporting Workers to Sit Less and Move More Through the Web-Based BeUpstanding Program: Protocol for a Single-Arm, Repeated Measures Implementation Study. JMIR Res Protoc. 2020 May;9(5):e15756.

5. Hart SG, Staveland LE. Development of NASA-TLX (Task Load Index): Results of Empirical and Theoretical Research. In: Hancock PA, Meshkati NBT-A in P, editors. Human Mental Workload. North-Holland; 1988. p. 139–83.

6. Almghairbi DS, Marufu TC, Moppett IK. Anaesthesia workload measurement devices: qualitative systematic review. BMJ Simul Technol Enhanc Learn. 2018 Jul 1;4(3):112 LP – 116.

7. Collins CE, Boggess MM, Watson JF, Guest M, Duncanson K, Pezdirc K, et al. Reproducibility and comparative validity of a food frequency questionnaire for Australian adults. Clin Nutr. 2014 Oct;33(5):906–14.

8. Brakenridge CL, Fjeldsoe BS, Young DC, Winkler EAH, Dunstan DW, Straker LM, et al. Organizational-Level Strategies With or Without an Activity Tracker to Reduce Office Workers’ Sitting Time: Rationale and Study Design of a Pilot Cluster-Randomized Trial. JMIR Res Protoc. 2016 May;5(2):e73.

9. Australian Institute of Health and Welfare. The Active Australia Survey: A Guide and Manual for Implementation, Analysis and Reporting. Canberra: Australian Institute of Health and Welfare; 2003.

10. Wallmann-Sperlich B, Chau JY, Froboese I. Self-reported actual and desired proportion of sitting, standing, walking and physically demanding tasks of office employees in the workplace setting: do they fit together? BMC Res Notes. 2017;10(1):504.

11. Clark BK, Stephens SK, Goode AD, Healy GN, Winkler EAH. Alternatives for Measuring Sitting Accumulation in Workplace Surveys. J Occup Environ Med. 2021 Dec;63(12):e853–60.

12. Mensah K, Maire A, Oppert JM, Dugas J, Charreire H, Weber C, et al. Assessment of sedentary behaviors and transport-related activities by questionnaire: a validation study. BMC Public Health. 2016/08/11. 2016;16:753.

13. Hadgraft NT Healy GN, Lynch BM, Neuhaus M, Eakin EG, Dunstan DW, Owen N, Fjeldsoe BS. WEAH. Intervening to reduce workplace sitting: mediating role of social-cognitive constructs during a cluster randomised controlled trial. Int J Behav Nutr Phys Act. 2017;14(27).

14. Sallis JF, Grossman RM, Pinski RB, Patterson TL, Nader PR. The development of scales to measure social support for diet and exercise behaviors. Prev Med (Baltim). 1987 Nov;16(6):825–36.

15. Dunstan D, Wiesner G, Eakin E, Neuhaus M, Owen N, LaMontagne A, et al. Reducing office workers’ sitting time: rationale and study design for the Stand Up Victoria cluster randomized trial. BMC Public Health. 2013;13(1):1057.

16. Wilson PM, Rodgers WM, Loitz CC, Scime G. “It’s Who I Am … Really!’ The Importance of Integrated Regulation in Exercise Contexts1. J Appl Biobehav Res. 2006 Apr;11(2):79–104.

17. Clark BK, Lynch BM, Winkler EA, Gardiner PA, Healy GN, Dunstan DW, et al. Validity of a multi-context sitting questionnaire across demographically diverse population groups: AusDiab3. Int J Behav Nutr Phys Act. 2015 Dec;12:148.

18. Gomersall SR, Skinner TL, Winkler E, Healy GN, Eakin E, Fjeldsoe B. Feasibility, acceptability and efficacy of a text message-enhanced clinical exercise rehabilitation intervention for increasing ‘whole-of-day’ activity in people living with and beyond cancer. BMC Public Health. 2019;19(2):542.

19. Reilly MC, Zbrozek AS, Dukes EM. The validity and reproducibility of a work productivity and activity impairment instrument. Pharmacoeconomics. 1993 Nov;4(5):353–65.

20. Edwardson CL, Biddle SJH, Clarke-Cornwell A, Clemes S, Davies MJ, Dunstan DW, et al. A three arm cluster randomised controlled trial to test the effectiveness and cost-effectiveness of the SMART Work & Life intervention for reducing daily sitting time in office workers: study protocol. BMC Public Health. 2018;18(1):1120.

21. Nagy MS. Using a single-item approach to measure facet job satisfaction. J Occup Organ Psychol. 2002 Mar 1;75(1):77–86.

22. Schaufeli WB, Shimazu A, Hakanen J, Salanova M, De Witte H. An ultra-short measure for work engagement: The UWES-3 validation across five countries. Vol. 35, European Journal of Psychological Assessment. Schaufeli, Wilmar B.: Research Unit Occupational & Organizational Psychology and Professional Learning, KU Leuven, PO Box 3725, Leuven, Belgium, 3000, wilmar.schaufeli@kuleuven.be: Hogrefe Publishing; 2019. p. 577–91.

23. Lerner D, Amick III BC, Rogers WH, Malspeis S, Bungay K, Cynn D. The work limitations questionnaire. Med Care. 2001;72–85.

24. Herrmann C. International experiences with the Hospital Anxiety and Depression Scale--a review of validation data and clinical results. J Psychosom Res. 1997 Jan;42(1):17–41.

25. Hermanns N, Kulzer B, Krichbaum M, Kubiak T, Haak T. How to screen for depression and emotional problems in patients with diabetes: comparison of screening characteristics of depression questionnaires, measurement of diabetes-specific emotional problems and standard clinical assessment. Diabetologia. 2006 Mar;49(3):469–77.

26. Hann DM, Jacobsen PB, Azzarello LM, Martin SC, Curran SL, Fields KK, et al. Measurement of Fatigue in Cancer Patients: Development and Validation of the Fatigue Symptom Inventory. Qual Life Res. 1998;7(4):301–10.

27. Andersen LL, Clausen T, Burr H, Holtermann A. Threshold of musculoskeletal pain intensity for increased risk of long-term sickness absence among female healthcare workers in eldercare. PLoS One. 2012;7(7):e41287.

28. Palmer K, Smith G, Kellingray S, Cooper C. Repeatability and validity of an upper limb and neck discomfort questionnaire: the utility of the standardized Nordic questionnaire. Occup Med (Chic Ill). 1999 Apr 1;49(3):171–5.

29. Dietch JR, Taylor DJ. Evaluation of the Consensus Sleep Diary in a community sample: comparison with single-channel electroencephalography, actigraphy, and retrospective questionnaire. J Clin sleep Med JCSM Off Publ Am Acad Sleep Med. 2021 Jul;17(7):1389–99.

30. Richardson J, Iezzi A, Khan MA, Chen G, Maxwell A. Measuring the Sensitivity and Construct Validity of 6 Utility Instruments in 7 Disease Areas. Med Decis Mak an Int J Soc Med Decis Mak. 2016 Feb;36(2):147–59.

31. Eakin EG, Reeves MM, Marshall AL, Dunstan DW, Graves N, Healy GN, et al. Living Well with Diabetes: a randomized controlled trial of a telephone-delivered intervention for maintenance of weight loss, physical activity and glycaemic control in adults with type 2 diabetes. BMC Public Health. 2010/08/04. 2010;10:452.
