# Supplementary material for: Sitting less and moving more for improved metabolic and brain health in type 2 diabetes: ‘OPTIMISE your health’ trial protocol
Source: BMC Public Health. 2022 May 10;22:929. doi: 10.1186/s12889-022-13123-x (PMC9086419; doi:10.1186/s12889-022-13123-x)
Supplement: Supplementary file 10 — Additional file 10. Adverse events report. [file 12889_2022_13123_MOESM10_ESM.docx]

Reporting Adverse Events to the Alfred Hospital HREC for Clinical Trials

***It is the responsibility of investigators to identify and report significant safety information to the Ethics Committee***


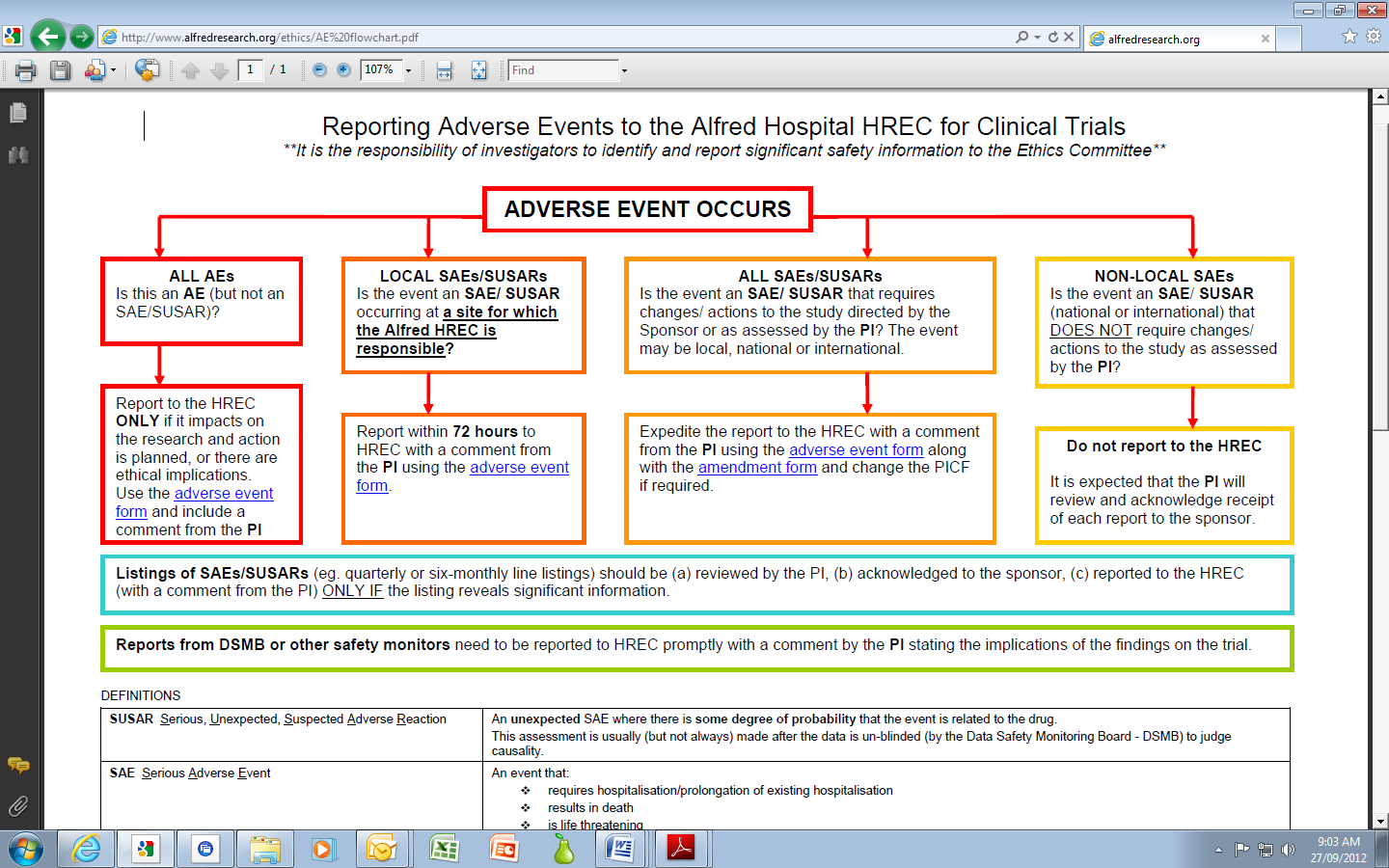


*DEFINITIONS*

| **SUSAR** | Serious, Unexpected, Suspected Adverse Reaction | An **unexpected** SAE where there is **some degree of probability** that the event is related to the drug.  This assessment is usually (but not always) made after the data is un-blinded (by the Data Safety Monitoring Board - DSMB) to judge causality. |
| --- | --- | --- |
| **SAE** | Serious Adverse Event | - requires hospitalisation/prolongation of existing hospitalisation - results in death - is life threatening - results in persistent or significant disability/incapacity - is a congenital anomaly/birth defect; or - is a medically important event or reaction |
| **AE** | Adverse Event | Any untoward event that does not necessarily have a causal relationship with the treatment. These may be expected (defined in the Investigator Brochure) |
| **PI** | Principal Investigator | Principal Investigator or a co-investigator delegated this responsibility |

**ADVERSE EVENT (AE) FORM**

*(All pages must be completed for SAEs and SUSARs)*

| **Adverse Event**  list one sign/symptom per line | **Date of onset** | | | **Date of resolution** | | | **Maximum intensity^1^**  1 = mild  2 = moderate  3 = severe | **Action taken as a result of the AE**  1 = no action taken  2 = CMED added  3 = CMED discontinued  4 = CMED dose change | **Withdrawal**  Did the subject withdraw from study as a result of this event?  Y = Yes  N = No | **Study relationship^2^**  1 = unrelated  2 = possibly  3 = probably  4 = definitely | **Outcome^3^**  1 = resolved  2 = resolved with sequelae  3 = Not resolved |
| --- | --- | --- | --- | --- | --- | --- | --- | --- | --- | --- | --- |
| *Eg. Nausea* | *25* | *01* | *2012* | *27* | *01* | *2012* | *2* | *1* | *N* | *2* | *1* |
| 1. |  |  |  |  |  |  |  |  |  |  |  |
| 2. |  |  |  |  |  |  |  |  |  |  |  |
| 3. |  |  |  |  |  |  |  |  |  |  |  |
| 4. |  |  |  |  |  |  |  |  |  |  |  |
| 5. |  |  |  |  |  |  |  |  |  |  |  |
| 6. |  |  |  |  |  |  |  |  |  |  |  |

Definition: CMED = Concomitant medication; ^1^Record maximum intensity. ^2^Indicate only for specific events if there is a reasonable possibility the event was caused by the study. ^3^All AEs must be followed until the events are resolved, the condition stabilises, the events are otherwise explained, or the subject is lost-to-follow up. If the adverse event is ongoing at study completion or lost-to-follow up, then the outcome is “not resolved”.

1. **Relevant medical conditions:**

| Specify relevant past or current medical disorders, allergies, surgeries that may help explain the AE | Date of onset  Day/Month/Year | Condition present at the time of the AE? Y = Yes, N = No | Date of the last occurrence  Day/Month/Year |
| --- | --- | --- | --- |
| a. |  |  |  |
| b. |  |  |  |
| c. |  |  |  |
| d. |  |  |  |
| e. |  |  |  |

1. **Concomitant medications:**

| Medication | Dose | Frequency | Started >30days prior to AE or SAE | Continuing medication?  Y = Yes, N = No |
| --- | --- | --- | --- | --- |
|  |  |  |  |  |
|  |  |  |  |  |
|  |  |  |  |  |
|  |  |  |  |  |
|  |  |  |  |  |
|  |  |  |  |  |

1. **Other relevant risk factors (eg. Family history, smoking, alcohol, diet, drug abuse, occupational hazard relevant to this event):**

| a. |
| --- |
| b. |
| c. |
| d. |
| e. |

1. **Narrative remarks:**

|  |
| --- |
|  |
|  |
|  |
|  |
|  |
|  |
|  |

Have additional pages been used? Yes No

Has Ethics Committee been notified? Yes No Date HREC was notified: / /

Principal Investigator:_________________________________________________________

Investigator Contact Phone: ________________________

Investigator signature:_____________________________________ Date: / /

**Alfred Ethics Process of Reporting of Adverse Events**

**Reporting to the Ethics Committee**

The NHMRC Australian Health Ethics Committee (AHEC) Position Statement on [**Monitoring and Reporting of Safety for Clinical Trials Involving Therapeutic Products**](http://www.alfredresearch.org/ethics/NHMRC%20Position%20Statement%20AE%20Reporting.pdf) sets out requirements for the reporting of adverse events in accordance with the National Statement on Ethical Conduct in Human Research (2007). The Alfred Health Human Ethics Committee has adopted the requirements set out in the Position Statement with some minor refinements, as detailed in the Alfred Health Human Ethics Committee [**Safety Monitoring and Reporting Requirements**](http://www.alfredresearch.org/ethics/Safety%20Monitoring%20and%20Reporting%20Requirements.pdf).The [**Adverse Event Flow Chart**](http://www.alfredresearch.org/ethics/AE%20flowchart.pdf) provides an easy reference for researchers. ***Please read all three documents.***

An [**Adverse Events Report Form**](http://www.alfredresearch.org/ethics/SAE%20report%20form%20July%202012.doc) must be submitted with each report or group of reports. This form must be completed and emailed or electronically signed by the principal researcher.

Please email the completed form to [**research@alfred.org.au**](mailto:research@alfred.org.au). An acknowledgement of receipt will be sent by return email.

If the Ethics Committee requires subsequent action, researchers will be notified by further email.

**Reporting to the insurers**

If the event is possibly, probably or definitely related to a study drug or procedure, it will need to be referred to the Hospital insurers in case a claim is subsequently made against Alfred Health. The Ethics Office will instruct researchers on how to do this.

**Reporting to sponsors**

Commercial sponsors will instruct researchers on their adverse event reporting requirements.

**Reporting to the TGA**

For researcher-initiated projects, serious adverse events related to drugs or devices are to be reported to the [**Therapeutic Goods Administration (TGA)**](http://www.tga.gov.au/safety/problem.htm)

ALFRED HOSPITAL ETHICS COMMITTEE

Adverse Events Form

***Significant events concerning Alfred Health participant safety***

***must be reported to the Ethics Office within 72 hours.***

- *Please email this form and any attachments to* [*research@alfred.org.au*](mailto:research@alfred.org.au?subject=Protocol%20deviation%20/%20violation) *with the Alfred project number inserted in the subject line.*
- *If the email is not sent from the principal researcher’s email address, the principal researcher’s electronic signature must be inserted*

| **Alfred Project No:** |  |
| --- | --- |
| **Project Title:** |  |
| **Name of Principal Researcher:** |  |
| **Event site:** | Site under the jurisdiction of The Alfred Hospital Ethics Committee  External site outside the jurisdiction of The Alfred Hospital Ethics Committee |
| **CTN:** | Not applicable |

| Participant reference (eg. code number) | Details of Event | Report type (initial or follow-up) | Relationship to study drug/device/intervention (unrelated, possibly, probably, definitely) | Expected or Unexpected |
| --- | --- | --- | --- | --- |
|  |  |  |  |  |

**Recommendation by the Principal Researcher:**

- Change to the protocol Y/N

1. Change to the Participant Information and Consent Form Y/N
2. Previously enrolled participants to be notified Y/N
3. Study to be stopped Y/N
4. No action Y/N

**Principal Researcher’s comments:**

*Please comment on (a) the context and significance of the event, (b) the possible impact on participants, and (c) action taken or recommended (add any relevant attachments)*:

|  |
| --- |

**Principal Researcher’s signature: Date:**

| Contact person’s name: |  |
| --- | --- |
| Position: |  |
| Email: |  |
